# HPV vaccine knowledge and acceptability among Peruvian men who have sex with men and transgender women: A pilot, qualitative study

**DOI:** 10.1371/journal.pone.0172964

**Published:** 2017-02-28

**Authors:** Jerome T. Galea, Emmi Monsour, César R. Nureña, Magaly M. Blas, Brandon Brown

**Affiliations:** 1 Socios En Salud, Lima, Peru; 2 Epicentro Salud, Lima, Peru; 3 Department of Global Health and Social Medicine, Harvard Medical School, Boston, Massachusetts, United States of America; 4 Center for Healthy Communities, School of Medicine, University of California, Riverside, California, United States of America; 5 School of Anthropology, Universidad Nacional Mayor de San Marcos, Lima, Perú; 6 School of Public Health and Administration, Universidad Peruana Cayetano Heredia, Lima, Peru; 7 Center for Healthy Communities, School of Medicine, University of California, Riverside, Riverside, California, United States of America; University of Washington, UNITED STATES

## Abstract

**Purpose:**

Human papillomavirus (HPV) is the most common sexually transmitted infection globally and is responsible for a variety of cancers in men and women. An effective HPV vaccine licensed for use in girls and boys has been indicated for—but is not widely implemented in—men who have sex with men (MSM). Limited data are available for transgender women (TW). We explored the social and behavioral aspects related to HPV vaccine uptake and participation in HPV vaccine studies among Peruvian MSM and TW.

**Methods:**

Focus groups and individual in-depth interviews were conducted to obtain the knowledge, thoughts, and opinions from Peruvian MSM and TW regarding HPV vaccination. Data were analyzed using systematic comparative and descriptive content analysis.

**Results:**

Three focus groups and fifteen individual in-depth interviews were conducted among 36 MSM and TW. Participant mean age was 26 years (range 18–40). Though many participants were unfamiliar with HPV vaccination, most expressed positive attitudes. Participants expressed concerns about the potential for stigma when disclosing HPV vaccination.

**Conclusion:**

Peruvian MSM and TW felt that HPV vaccination would be acceptable to themselves and their peers. Nonetheless, vaccine intake may be impeded by potential stigma. Findings from this study may guide HPV vaccine implementation in similar populations.

## Introduction

Human papillomavirus (HPV) is one of the most common sexually transmitted infections (STIs). Worldwide, an estimated 630 million people are currently infected with another 500,000 new infections annually [1]. In addition to causing genital warts (GW), HPV is also a cause of cervical, penile, anal, and some head and neck cancers [2]. Whereas HPV prevalence in heterosexually identified men is estimated at ≤50% [3], prevalence in men who have sex with men (MSM) is estimated at 61% (HIV negative) and 93% (HIV positive) [4]. Likewise, MSM are disproportionally affected by HPV-related sequela than non-MSM, including higher rates of anogenital and oropharyngeal cancers [5]. Limited data are available for HPV rates among transgender women (TW), but studies are emerging. One study in Peru reported a prevalence of any anogenital HPV infection among 68 TW at 95.6%, noting that half of these women were infected with high risk (i.e. oncogenic) HPV types [6]; a study among 35 TW in Italy reported HPV DNA in 40% of the subjects, with high risk HPV types detected in 93% of TW co-infected with HIV [7].

Available HPV vaccines provide significant protection against types 6 and 11, responsible for 90% of GW, and types 16, 18, 31, 33, 45, 52, and 58, responsible for 70–90% of anogenital cancers [8]. U.S. immunization policies specifically recommend HPV vaccination for MSM up to 26 years of age and, as of December 2016, recommend vaccination for transgender persons to be the same as for all adolescents but extending to age 26 in case of previous inadequate vaccination [9]. This policy is congruent with evidence from a modeling study finding HPV vaccination among MSM in the U.S. likely to be cost effective for GW and anal cancer prevention [10]. Despite the benefits of HPV vaccination, however, uptake among MSM is significantly lower than the general population [11], and uptake among TW is unknown. In a 2011 online household sample of 1457 U.S. MSM aged 18–26 years, just 6.8% had received one HPV vaccine dose; vaccine uptake was associated with recent STI testing, disclosure of being gay or bisexual to a doctor, and higher levels of HPV knowledge [12]. In a 2014 systematic review of 16 studies including 5185 MSM (mainly from North America) on HPV and vaccine-related perceptions, MSM: 1) poorly understood the role of HPV on anogenital and oral cancers; 2) saw themselves at risk for HPV; and 3) had increased willingness to receive vaccination related to HPV awareness [13].

In Peru, routine and free HPV vaccine is administered only to adolescent girls in school settings [14] despite an increasingly-documented high burden of HPV in MSM and TW [6, 15, 16]. Peruvian MSM and TW are often aware of HPV by the presence of GW (which is frequently a source of treatment-related shame, stigma, and discrimination) rather than by cancer manifestations [17]. With the Peruvian HIV epidemic concentrated among MSM and TW (estimated at 12.4% and 20.8%, respectively, compared with 0.40% in the general population [18]), these populations could particularly benefit from HPV vaccination because of the synergistic relationship between HIV and HPV infection [19]. The purpose of this exploratory pilot study was to characterize the knowledge and beliefs of Peruvian MSM and TW regarding HPV vaccination in order to better inform its potential introduction into these populations.

## Methods

### Participants

Between January and September 2011, we recruited both MSM and TW to participate in either a focus group or an individual, in-depth interview. Convenience-based recruitment was conducted by peer outreach workers at *Epicentro Salud*, a community health center serving MSM and TW in Lima, Peru, and at bars, clubs, volleyball courts, saunas, and parks frequented by these populations. Peer outreach workers visited the various venues, approached potential participants, and explained the purpose of the study as a research project to understand the views, opinions and knowledge of MSM and TW on HPV treatment and prevention. To ensure sample diversity, we recruited MSM who self-identified as ‘‘gay” as well as those not self-identifying as gay, and MSM and TW who reported paid sex work as well as those who did not. Interested individuals were referred to the study site where they were screened for eligibility (≥18 years of age, reported sex with a male in the previous 12 months) and assigned to participate in either a focus group or an individual, in-depth interview.

### Procedures

The focus groups and the individual, in-depth interviews took place in a private room; each lasted approximately 60 minutes. Two Peruvian psychologists experienced in qualitative methods and HIV/STI prevention with MSM and TW conducted the sessions that were audio recorded on digital media. At the beginning of focus groups and interviews, participants received a verbal explanation of HPV including information regarding: transmission; prevalence; types; GW; common HPV-related cancers (cervical and anal); Peruvian MSM and TW as special populations disproportionately affected by HPV; and HPV and other STIs including HIV. Photographs of anogenital GW were shown to assure comprehension, and participants were encouraged to ask questions at any time during the introduction or during the focus groups or interviews.

Focus groups and in-depth interviews used a semi-structured guide with questions and probes on HPV vaccination including: vaccine knowledge, acceptability, social and community concerns (including the impact of vaccines on an individual’s social life and sexual practices), and participation in HPV vaccine research. Table 1 shows the main question domains, types, and probes used for both the focus groups and interviews.

**Table 1:** Guide for focus groups and in-depth interviews among Peruvian men who have sex with men and transgender women regarding HPV and HPV vaccine acceptability.

Domain	Question	Probes
**HPV Vaccine**	Do you know if there is a vaccine that prevents HPV infection?	What have you heard?
**Facilitators and Barriers to HPV vaccination**	If there were an HPV vaccine available to you in Peru, would you vaccinate yourself?	Why or why not?
	Do you think your friends would get vaccinated?	Why or why not?
	Do you think your life would change if you were to get vaccinated?	How so?
**Behavioral Issues**	Do you think people would change their sexual behaviors if they were to get vaccinated?	How would they change? To what extent could behaviors change?
	Do you think that condom use by MSM and TW could change after getting vaccinated for HPV?	What about protection against other sexually transmitted infections besides HPV?
**Cultural and Social Issues**	Do you think that men who have sex with men and transgender women in Peru are ready to get the HPV vaccine?	Why or why not?
	Do you think your family or friends would question you or bother you if you were to decide to get vaccinated?	Why or why not?
	If you decided to get vaccinated would you tell your friends or family?	What differences might there be between different groups?
**HPV Vaccine Research**	What do you think about the possibility of an HPV vaccine study being conducted in Peru for MSM and TW?	Would you participate? Why or why not?
	What might convince you to participate?	

All participants provided informed consent prior to study participation and received 15 *Nuevos Soles* (approximately US $5.60 in 2011) for transportation. Ethics approval was obtained from the *Universidad Peruana Cayetano Heredia*.

### Data analysis

Audio recordings were transcribed verbatim. A Peruvian anthropologist experienced in STI research (CRN) performed a systematic, comparative, and descriptive content analysis by coding and grouping the information thematically, extracting similarly coded data into matrices, and identifying recurring issues and differences in the narratives. A second, bilingual reviewer (JTG) confirmed the analysis and discrepancies were mutually resolved. Representative quotes for each theme were translated into English for results reporting. Data are sorted as either from an individual interviewee or focus group and from MSM or TW.

## Results

Three focus groups (two with MSM and one with TW) and fifteen individual, in-depth interviews (10 MSM, 5 TW) were conducted for a total sample size of 36 MSM and TW participants. The mean participant age was 26 (range 18–40). The main emergent themes included: the purpose of vaccines in general and the HPV vaccine in particular; acceptability and motivation for HPV vaccination; HPV vaccination and sexual behavior; social aspects and HPV vaccination; and attitudes towards HPV vaccination research. Table 2 includes additional quotes supplementing the illustrative quotes presented in this section; see S1 Data for the unabridged qualitative data analysis. For the in-depth interviews, the number identifier refers to an individual participant.

**Table 2:** HPV vaccine knowledge and acceptability among Peruvian men who have sex with men and transgender women: Additional quotes supporting each of the identified themes.

Theme	Representative Quotes*
**Purpose of vaccines and the HPV vaccine**	*“There is an…injection that helps control the virus*, *right*?*”* **(Interviewee-10, TW)** *"I would [like to] receive the vaccine to cure me or to keep me from being infected with warts*.*"* **(Interviewee-13, MSM)** *“[A vaccine] controls the [body’s] defenses*, *doesn’t it*?*”* **(Focus Group, MSM)** *“[A vaccine] provides defense against what is going to infect us; we have defenses for that to control it*.*”* **(Focus Group, MSM)**
**Acceptability and motivation for HPV vaccination**	*“I would be a role model example [for others]*. *Better yet*, *I would say*, *‘I already received the HPV vaccine*,*’ and it would benefit me because I would be safe against infection*.* *.* *.*”* **(Interviewee-11, MSM)** *“I would receive the vaccine because I am going to … prevent [warts]*, *right*? *In other words… I’m going to be protected with [the vaccine]*, *right*? *However much intercourse I have*, *I think I will be cured against HPV*.*”* **(Interviewee-6, TW)** *“I have a girlfriend and she doesn’t know everything about me*. *I do not want to infect her*.*”* **(Interviewee-3, MSM)** *“There is not much information about HPV*.*”* **(Focus Group, MSM)** “*MSM are not prepared to receive the vaccine because they are not aware of the issue*, *and some will not do it of their own accord*. *In other words*, *they either don’t know about it or they ignore it*.” **(Interviewee-5, MSM)**
**HPV vaccination and sexual behavior**	*“You get the HIV test*, *you take care*, *but it does not end there because there are other [sexually transmitted infections*].” **(Focus Group, MSM)** *“Yes [I would continue using condoms]*, *because the condom protects you from all diseases*, *while the vaccine only protects for HPV*.*”* **(Interviewee-9, MSM)** *“If I received the vaccine*, *I would be protecting my sexual partner*, *right*? *Sexually speaking*?*”* **(Interviewee-2, MSM)**
**Social aspects and HPV vaccination**	*“I am an independent person and I make my own decisions*. *If I mention it to them [my family]*, *they might be uncomfortable*, *but it is up to me to tell them or not*.*”* **(Interviewee-2, MSM)** *“No [I would not tell my friends]; it’s personal and they don’t have to know*. *This decision is mine*, *and they will mock me if I am vaccinated because they see it as a weakness*. *They will think I like faggots*, *and then the entire neighborhood will know*. *They would make fun of me*, *and I know my brothers and friends would participate [in ridiculing me]*.*”* **(Interviewee-13, MSM)**
**Attitudes towards HPV vaccine research participation**	*"I would get vaccinated as long as there are no abnormal reactions*, *and I didn’t have any problems [getting the vaccine]*.*"* **(Interviewee-10, TW)** *“[The studies] sound good*. *Many things can be avoided*, *I don’t know*, *to avoid warts*, *right*? *Sex workers are more exposed to that*.” **(Interviewee-7, MSM**) *“I think [whether one participates] depends on many things*, *including shame and fear*.*”* **(Focus group, MSM)** *“Like I told you before*, *I will come running to new projects*. *I am always looking for the treatments*.*"* **(Interviewee-8, TW)**

*Numbers for Interviewees are used to distinguish between different participants.

MSM: men who have sex with men; TW: transgender woman

### Purpose of vaccines and the HPV vaccine

Most participants grasped the preventive aspect of vaccines in general, though there was confusion among some whether vaccines cure, treat, or control disease:

“*[A vaccine is] to control or heal a certain disease.*”

(Focus Group, MSM)

"*I think if I get vaccinated against the infection, I am going to ‘nip the infection in the bud,’ … like a treatment. It won’t be overnight, and it will be slow, but it will be a treatment.*"

(Interviewee-3, MSM)

### Acceptability and motivation for HPV vaccination

Some participants had previously heard about the HPV vaccine and the majority expressed interest in receiving it. Reasons mentioned for accepting the vaccine were the prevention of GW, avoiding infecting others with HPV, as a way to get more information about HPV and GW, and providing a sense of security and serving as a role model for others:

"*I would like to protect myself, to not infect others.*"

(Interviewee-2, MSM)

"*My friends, transgender people, would accept vaccination because no one wants warts in the anus.*"

(Interviewee-7, TW)

Vaccine cost was also mentioned as a factor related to vaccine uptake:

"*The truth is that I didn’t know about HPV vaccines. But if they are implemented, and they are free, yes, I would get it now.*"

(Interviewee-4, MSM)

The potential for vaccine acceptance by peers was expressed in different ways. Some thought such vaccines would be well-received by others due to its protective benefits while others believed their friends downplayed the risk of STI, or they would prefer to maintain private matters relating to sex with MSM or TW.

"*Interviewer: Do you think that your friends here in the neighborhood, both the ones you hang out with and the ones that tend to have sex with men, would get vaccinated?*

*Participant: I don’t know; it is the individual’s choice! Here, they are all on the down-low, always denying they have sex with men.*"

(Interviewee-14, MSM)

### HPV vaccination and sexual behavior

Several participants thought receiving the vaccine would encourage or increase condom use and STI information seeking:

“*In my case, I would continue using condoms because I would not be protected from other diseases or AIDS, right?*"

(Interviewee-13, MSM)

“*[Being vaccinated] serves as a warning and makes me more conscious of my behavior.*"

(Interviewee-12, MSM)

When asked if sexual conduct would change once vaccinated for HPV, however, some participants predicted increased sexual risk-taking due to a bolstered sense of safety:

“*They will feel more secure [once vaccinated for HPV], right? Or maybe they will be more irresponsible because they will say, ‘I’ve been vaccinated, and I am not at risk of being infected with that [HPV].’*”

(Interviewee-2, MSM)

### Social aspects and HPV vaccination

Many participants said they would tell their families and friends if they were vaccinated. One reason for this disclosure was perception of vaccination as a “health issue” carrying both disease prevention benefits and potential risk:

“*…if I tell [my family] that I am going to be vaccinated to prevent disease, I think that they would support me.*"

(Interviewee-6, TW)

“*My family should know because vaccines are unpredictable and there are so many side effects; I would want them to be aware if something happens to me.*"

(Interview-14, MSM)

However, predictions of vaccine disclosure acceptance was not universal, especially among participants’ friends:

“*My friends would say, ‘Why are you doing so much to control the disease? We all die from something.’*”

(Interviewee-10, TW)

Stigma was predicted to be a potential outcome when disclosing one’s HPV vaccination to others, but may be managed in different ways depending on the audience. For example, one interviewee said that he could tell his family about his HPV vaccination, and though their reaction could be to confuse vaccination with HPV infection, he could nonetheless correct their misunderstanding. With friends, however, his strategy would be to refrain from disclosing HPV vaccination so as to avoid potential ridicule:  

“*[My family] would think I had the disease, or that I was affected by it. I would tell them that the vaccine is prevention, though. I think, for my sake, they would not say anything. I wouldn’t say anything to my friends until my results came out. They might take it the wrong way, make fun of me, or think I am infected.*"

(Interviewee-5, MSM)

### Attitudes towards HPV vaccine research participation 

Study participants mainly endorsed HPV vaccine research, citing positive health benefits and taking advantage of research in Peru as incentives:

“*They are doing good for the health of the people. As a MSM person, I would receive [the vaccine]; everyone should be concerned about their health.*”

(Interviewee-4, MSM)

“*[Peru] seems perfect to me [for an HPV vaccine study], right? Thank God they provide this opportunity here and not somewhere else. We are a part of the science.*"

(Interviewee-9, TW)

Among the reasons cited for not wanting to participate in HPV vaccine research were potential side effects, or “abnormal reactions” and one TW specifically noted the vaccine injection site as a concern since gender conforming implants could interfere:

“*Some medical providers have learned to ask if transgender women have silicone in their backsides because they cannot use the penicillin shot there and must use the arm instead.*”

(Focus Group, TW)

As for vaccine disclosure, concerns were raised about what others may say about their participation in a HPV-related study, in this case going to a research site that was known for work with MSM:

“*People don’t want to [come to the study site for research] because they are not in the scene. They say: ‘It is just queers, it is just for queers.’*”

(Focus group, MSM)

## Discussion

This is the first study in Peru, and one of very few globally, specifically investigating the acceptability of HPV vaccination in MSM and TW. The chief finding is that the acceptability of a preventive HPV vaccine was widespread but not universal among these populations depending on a range of factors. While HPV vaccination was sometimes incorrectly perceived as curative or therapeutic, rather than preventive, reasons for wanting HPV vaccination centered on not only self-protection but the protection of sexual partners (including female partners), prevention of GW, and to serve as a role model for others.

Chief among the barriers to HPV vaccination, as well as participation in HPV vaccination research, was the potential for physical and social vaccine-induced harm. Physical harm is a concern easily addressed given the vaccines’ excellent safety record and expectation that it will “do no harm” (i.e., be free from side effects). TW’s concerns about the vaccine administration interfering with silicone implants located in the buttocks can be managed by explaining that the vaccine site is the muscle in the upper arm.

Concerns regarding possible social harms, however, will be harder to assuage. MSM in particular worried about the stigma associated with their sexual behaviours, an understandable concern in Peru where 8 assassinations due to homophobia occurred in the past year alone [20]. Similar concerns have been documented in these populations in Peru regarding the acceptability of preexposure prophylaxis (PrEP) for HIV infection [21] and rectal microbicides [22]. Unfortunately, though participants’ concerns regarding stigma were related to their friends and family, the health delivery system presents additional challenges. Access to HPV vaccination (as with other sexual health services) relies on an accurate sexual history which is often hindered by MSM and TW’s fear of discrimination [23, 24]. This issue is compounded by the fact that perceived susceptibility to HPV-related cancers and genital warts is often low among MSM to begin with [25] (cancer prevention was not mentioned in neither the focus groups nor the in-depth interviews as a motivating factor for HPV vaccination) thus patients may not even think to ask their medical provider about these issues. Though to do so would require an increased investment, the only sure path to reaching all MSM and TW would be to implement universal vaccination for all adolescents, girls and boys. Besides increased costs, opponents of this approach cite “herd immunity” which develops after 50% of the female population is vaccinated thereby conferring protection to their unvaccinated partners [26]. Unfortunately, however, not only is the HPV vaccination rate of girls in Peru well below 50% [27], but for MSM and TW, herd immunity based on the vaccination of females would likely provide little or no protection. Universal vaccination could avoid sex and gender inequalities, boost overall vaccination and protection rates regardless of sex, gender and sexual orientation, and preempt potentially stigmatizing situations faced by adult MSM and TW requesting the vaccine.

Risk compensation (or sexual disinhibition) surfaced as a possibility by one participant who felt that peers could be sexually “irresponsible” following HPV vaccination, though this was a minority view. Risk compensation among vaccine-aged adolescent girls and boys is still hotly debated (particularly among parents) despite a lack of evidence that either riskier sexual behaviors or STI rates increase post HPV vaccination [28]. Given that a similar discourse continues in the HIV biomedical prevention field among MSM and TW [29], research is necessary to further understand the potential for sexual behavior changes among MSM and TW after receiving HPV vaccination.

Another factor which was mentioned in our study as affecting HPV vaccine uptake and requires deeper investigation is the effect of vaccine price on vaccine uptake. In the Peruvian HPV vaccination program, given that the program has not been extended to boys, inclusion of MSM and TW in the immediate future seems improbable. Not surprisingly, price had a profound effect on HPV vaccine acceptability in a sample of Hong Kong MSM, with 80% of participants finding a vaccine acceptable when it was free, compared to 30% when it was offered at the current market price [30]. In Peru, product price has also been demonstrated to influence the potential uptake of novel HIV prevention interventions, with a clear preference for low- or no cost alternatives (even in scenarios where other product characteristics such efficacy and side effects lessened) [21, 31, 32]. Unfortunately, these data do not bode well for wide-spread, out-of-pocket HPV vaccination, especially in lower and middle income countries like Peru, where 3 doses of Cervarix^®^ and Gardasil^®^ currently cost $195 and $270 respectively, or approximately 57%-78% of an the average Peruvian monthly salary [33]. Thus, HPV vaccination for MSM and TW will be out of reach except for the few who can pay for it unless either all adolescent boys (regardless of sexual and gender orientation) are included in the National Vaccine Plan or, in lieu of routine vaccination for boys, MSM and TW are strategically targeted for vaccination. Studies exploring the economic benefits of HPV vaccination for MSM and TW (e.g., number of genital wart cases requiring treatment averted; number of preventable HPV-associated cancers averted, etc.) should also be conducted to inform policy changes.

Limitations to our study are related to its small, formative nature that precluded several methodological enhancements necessary in future studies. More in-depth demographic and sexual behavior information from participants should be included in future studies in order to triangulate and further contextualize the findings and thereby strengthen its generalizability. Also, while we strived to recruit a study sample reflective of Peruvian MSM and TW which included MSM who did not identify as gay and both MSM and TW reporting paid sex work, the small overall sample size and thus sub-group sizes led to focus groups of all MSM or all TW, thereby preventing the ability to attribute specific quotes to MSM types. Finally, not all participants were < 26 years of age (the US CDC recommended cut-off age for HPV vaccination in MSM [9]); however, not only was this study conducted before this age recommendation was published, but this does not invalidate participant opinions for a vaccine they may have been eligible to receive. These limitations notwithstanding, this small study still represents the first step in exploring HPV vaccine acceptability among MSM and TW in Peru and to our knowledge in South America. Future studies should also consider expanding beyond the target vaccine populations to include the views and opinions of those who would act as the likely gateway not only to HPV vaccination but for education and treatment of HPV and its sequelae, as well: nurses, doctors and health professionals [17].

As Nadarzynski et al. [13] point out in their systematic review of literature examining HPV vaccine-related perceptions among MSM, in environments where HPV vaccination is sex-specific (as in Peru), theory-driven studies focusing on the specific demographic, behavioral and psychosocial factors related to vaccine uptake are essential. Particularly in young and/or sexually inexperienced MSM and TW, understanding with precision the factors predicting HPV vaccination uptake would lay the foundation for vaccine introduction into these populations, by either public or community-based entities.

## Conclusion

MSM and TW in Peru and elsewhere are disproportionally infected and affected with HPV and related sequelae, and co-infection with HIV greatly increases the rates of anogenital and oral cancers in these populations. Unlike HIV, however, safe and effective HPV vaccines have been available for years now with varied vaccine uptake success dependent on the setting. Therefore, in addition to future research on the individual factors affecting (or which could potentially affect) HPV vaccine uptake, implementation science should also be applied at the policy and healthcare delivery systems levels so that populations in particularly high need can receive the vaccine in a systematic and sustainable way.

## Supporting information

S1 Data Full unabridged qualitative data analysis. (DOCX)

## References

[1] Owsianka B, Ganczak M. Evaluation of human papilloma virus (HPV) vaccination strategies and vaccination coverage in adolescent girls worldwide. Przegl Epidemiol. 2015;69(1):53–8, 151–5 25862448

[2] Giuliano AR, Tortolero-Luna G, Ferrer E, Burchell AN, de Sanjose S, Kjaer SK, et al Epidemiology of human papillomavirus infection in men, cancers other than cervical and benign conditions. Vaccine. 2008;26 Suppl 10:K17–28. 18847554 10.1016/j.vaccine.2008.06.021 PMC4366004

[3] Dunne EF, Nielson CM, Stone KM, Markowitz LE, Giuliano AR. Prevalence of HPV infection among men: A systematic review of the literature. J Infect Dis. 2006;194(8):1044–57. 10.1086/507432 16991079

[4] Palefsky JM, Rubin M. The epidemiology of anal human papillomavirus and related neoplasia. Obstet Gynecol Clin North Am. 2009;36(1):187–200. 10.1016/j.ogc.2009.02.003 19344856

[5] Giuliani M, Vescio MF, Dona MG, Latini A, Frasca M, Colafigli M, et al Perceptions of Human Papillomavirus (HPV) infection and acceptability of HPV vaccine among men attending a sexual health clinic differ according to sexual orientation. Hum Vaccin Immunother. 2016;12(6):1542–50. 10.1080/21645515.2015.1115935 26752151 PMC4964721

[6] Brown B, Galea JT, Byraiah G, Poteat T, Leon SR, Calvo G, et al Anogenital Human Papillomavirus Infection and HIV Infection Outcomes among Peruvian Transgender Women: Results from a Cohort Study. Transgender Health. 2016;1(1):94–8. 29159301 10.1089/trgh.2016.0001 PMC5685267

[7] Loverro G, Di Naro E, Caringella AM, De Robertis AL, Loconsole D, Chironna M. Prevalence of human papillomavirus infection in a clinic sample of transsexuals in Italy. Sex Transm Infect. 2016;92(1):67–9. 10.1136/sextrans-2014-051987 26203116

[8] Petrosky E, Bocchini JA Jr, Hariri S, Chesson H, Curtis CR, Saraiya M, et al Use of 9-valent human papillomavirus (HPV) vaccine: updated HPV vaccination recommendations of the advisory committee on immunization practices. MMWR Morb Mortal Wkly Rep. 2015 3 27;64(11):300–4. 25811679 PMC4584883

[9] Meites E, Kempe A, Markowitz LE. Use of a 2-Dose Schedule for Human Papillomavirus Vaccination—Updated Recommendations of the Advisory Committee on Immunization Practices. MMWR Morb Mortal Wkly Rep. 2016 12 16;65(49):1405–1408. 10.15585/mmwr.mm6549a5 27977643

[10] Kim JJ. Targeted human papillomavirus vaccination of men who have sex with men in the USA: a cost-effectiveness modelling analysis. Lancet Infect Dis. 2010;10(12):845–52. 10.1016/S1473-3099(10)70219-X 21051295 PMC3982926

[11] Reagan-Steiner S, Yankey D, Jeyarajah J, Elam-Evans LD, Curtis CR, MacNeil J, et al National, Regional, State, and Selected Local Area Vaccination Coverage Among Adolescents Aged 13–17 Years—United States, 2015. Mmwr-Morbidity and Mortality Weekly Report. 2016;65(33):850–8 10.15585/mmwr.mm6533a4 27561081

[12] Cummings T, Kasting ML, Rosenberger JG, Rosenthal SL, Zimet GD, Stupiansky NW. Catching Up or Missing Out? Human Papillomavirus Vaccine Acceptability Among 18-to 26-Year-old Men Who Have Sex With Men in a US National Sample. Sex Transm Dis. 2015;42(11):601–6. 10.1097/OLQ.0000000000000358 26462183

[13] Nadarzynski T, Smith H, Richardson D, Jones CJ. Human papillomavirus and vaccine-related perceptions among men who have sex with men: a systematic review. Sex Transm Infect. 2014;90(7):515–U78. 10.1136/sextrans-2013-051357 24787367

[14] Ministerio de Salud del Perú (Peruvian Ministry of Health). Norma técnica de salud que establece el esquema nacional de vacunación (Technical standards establishing the national vaccination program). Ministerial Resolution. Lima, Perú: 2013 August 15, 2013. Contract No.: 510-2013/minsa

[15] Quinn R, Salvatierra J, Solari V, Calderon M, Ton TGN, Zunt JR. Human Papillomavirus Infection in Men Who Have Sex with Men in Lima, Peru. AIDS Res Hum Retroviruses. 2012;28(12):1734–8. 10.1089/AID.2011.0307 22519744 PMC3505064

[16] Blas MM, Brown B, Menacho L, Alva IE, Silva-Santisteban A, Carcamo C. HPV Prevalence in Multiple Anatomical Sites among Men Who Have Sex with Men in Peru. PLoS One. 2015;10(10). 10.1371/journal.pone.0139524 PMC4593601 26437318

[17] Nurena CR, Brown B, Galea JT, Sanchez H, Blas MM. HPV and Genital Warts among Peruvian Men Who Have Sex with Men and Transgender People: Knowledge, Attitudes and Treatment Experiences. PLoS One. 2013;8(3). 10.1371/journal.pone.0058684 PMC3597710 23516536

[18] Ministerio de Salud del Perú (Peruvian Ministry of Health). Informe nacional sobre los progresos realizados en el país (National report on progress made in the country). Lima, Perú: 2014. http://files.unaids.org/en/dataanalysis/knowyourresponse/countryprogressreports/2014countries/PER_narrative_report_2014.pdf

[19] Jin F, Prestage GP, Imrie J, Kippax SC, Donovan B, Templeton DJ, et al Anal sexually transmitted infections and risk of HIV infection in homosexual men. J Acquir Immune Defic Syndr. 2010;53(1):144–9. 10.1097/QAI.0b013e3181b48f33 19734801

[20] Sausa M. Ocho asesinatos por homofobia se registraron en el último año (Eight assinations due to homophobia were registered in the last year). Perú21. May 21, 2016. http://peru21.pe/actualidad/ocho-asesinatos-homofobia-se-registraron-ultimo-ano-2247081

[21] Galea JT, Kinsler JJ, Salazar X, Lee SJ, Giron M, Sayles JN, et al Acceptability of pre-exposure prophylaxis as an HIV prevention strategy: barriers and facilitators to pre-exposure prophylaxis uptake among at-risk Peruvian populations. Int J STD AIDS. 2011;22(5):256–62. 10.1258/ijsa.2009.009255 21571973 PMC3096991

[22] Galea JT, Kinsler JJ, Imrie J, Nurena CR, Ruiz L, Galarza LF, et al Preparing for Rectal Microbicides: Sociocultural Factors Affecting Product Uptake Among Potential South American Users. Am J Public Health. 2014;104(6):E113–E20. 10.2105/AJPH.2013.301731 24825222 PMC4062029

[23] Andrinopoulos K, Hembling J, Guardado ME, Hernandez FD, Nieto AI, Melendez G. Evidence of the Negative Effect of Sexual Minority Stigma on HIV Testing Among MSM and Transgender Women in San Salvador, El Salvador. AIDS Behav. 2015;19(1):60–71. 10.1007/s10461-014-0813-0 24907779

[24] Caceres CF, Aggleton P, Galea JT. Sexual diversity, social inclusion and HIV/AIDS. AIDS. 2008;22:S45–S55. 10.1097/01.aids.0000327436.36161.80 PMC3329729 18641469

[25] Colon-Lopez V, Toro-Mejias LM, Ortiz AP, Tortolero-Luna G, Palefsky JM. HPV Awareness and Willingness to HPV Vaccination among High-Risk Men attending an STI Clinic in Puerto Rico. P R Health Sci J. 2012;31(4):227–31 23844472 PMC3711188

[26] Stanley M., Perspective: Vaccinate boys too. Nature. 488 (2012) S10–S10. 10.1038/488S10a 22932433

[27] Bruni L, Diaz M, Barrionuevo-Rosas L, Herrero R, Bray F, Bosch FX, et al Global estimates of human papillomavirus vaccination coverage by region and income level: a pooled analysis. Lancet Glob Health. 2016 7;4(7):e453–63. 10.1016/S2214-109X(16)30099-7 27340003

[28] Kasting ML, Shapiro GK, Rosberger Z, Kahn JA, Zimet GD. Tempest in a teapot: A systematic review of HPV vaccination and risk compensation research. Hum Vaccin Immunother. 2016;12(6):1435–50. 10.1080/21645515.2016.1141158 26864126 PMC4964724

[29] Cassell MM, Halperin DT, Shelton JD, Stanton D. Risk compensation: the Achilles' heel of innovations in HIV prevention? BMJ. 2006;332(7541):605–7. 10.1136/bmj.332.7541.605 16528088 PMC1397752

[30] Lau JTF, Wang ZX, Kim JH, Lau M, Lai CHY, Mo PKH. Acceptability of HPV Vaccines and Associations with Perceptions Related to HPV and HPV Vaccines Among Men Who Have Sex with Men in Hong Kong. PLoS One. 2013;8(2). 10.1371/journal.pone.0057204 PMC3579800 23451188

[31] Tang EC, Galea JT, Kinsler JJ, Gonzales P, Sobieszczyk ME, Sanchez J, et al Using conjoint analysis to determine the impact of product and user characteristics on acceptability of rectal microbicides for HIV prevention among Peruvian men who have sex with men. Sex Transm Infect. 2016;92(3):200–5. 10.1136/sextrans-2015-052028 26574569

[32] Kinsler JJ, Cunningham WE, Nurena CR, Nadjat-Haiem C, Grinsztejn B, Casapia M, et al Using Conjoint Analysis to Measure the Acceptability of Rectal Microbicides Among Men Who Have Sex with Men in Four South American Cities. AIDS Behav. 2012;16(6):1436–47. 10.1007/s10461-011-0045-5 21959986

[33] Banco Central de Reserva de Peru (Central Reserve Bank of Peru) 2016 [cited 11/21/2016]. http://www.bcrp.gob.pe/.

