# Supplementary material for: HPV vaccine knowledge and acceptability among Peruvian men who have sex with men and transgender women: A pilot, qualitative study
Source: PLoS One. 2017 Feb 28;12(2):e0172964. doi: 10.1371/journal.pone.0172964 (PMC5330512; doi:10.1371/journal.pone.0172964)
Supplement: S1 Data — (DOCX) [file pone.0172964.s001.docx]

Virus del Papiloma Humano y Verrugas Genitales:

Estudio Cualitativo con Hombres que Tienen Sexo con Hombres de Perú

**INFORME FINAL DEL ANÁLISIS DE DATOS**

**(entrevistas en profundidad y grupos focales)**

*CÉSAR R. NUREÑA*

(Consultor)

Lima, diciembre de 2011

**CONTENIDOS**

**Introducción** (p. 2)

1. **Conocimientos sobre el VPH y las verrugas genitales** (p. 3)

Conocimientos y nociones sobre el VPH

Conocimientos y nociones sobre las verrugas genitales

Fuentes de información sobre el VPH y las VG

1. **Experiencias y actitudes relacionadas con las VG** (p. 8)

Experiencia de tener o haber tenido VG

Experiencias de haber visto VG en parejas u otras personas

Sin experiencia de haber tenido o visto VG

Vergüenza, silencio, estigma y discriminación

Preocupaciones ante la posible adquisición de VPH-VG

1. **Manejo de VG y atención de salud** (p. 21)

Prácticas asociadas al manejo de VG

Búsqueda de tratamiento y atención médica

Percepciones sobre la búsqueda de atención y los servicios de salud

1. **Vacunas VPH: conocimientos, aceptabilidad y actitudes** (p. 28)

Conocimiento de la existencia de vacunas contra el VPH

Aceptabilidad de la vacuna contra el VPH

Posibles reacciones del entorno social ante la vacunación

Posible impacto de la vacuna en la vida y las prácticas sexuales

Percepción sobre quiénes deberían recibir la vacuna

1. **Estudio de vacunas VPH: aceptabilidad y reclutamiento** (p. 47)

Aceptabilidad de un estudio de vacunas contra el VPH con hombres

Opiniones y recomendaciones sobre estrategias de reclutamiento

**Conclusiones** (p. 60)

**Recomendaciones** (p. 63)

**INTRODUCCIÓN**

El presente informe contiene los resultados del análisis de las transcripciones de 15 entrevistas en profundidad (ENT) y 3 grupos focales (GF), realizados con hombres de Lima en el contexto de una exploración cualitativa sobre la aceptabilidad de un estudio de vacunas para la prevención de la infección por el virus del papiloma humano (VPH) entre hombres que tienen sexo con otros hombres (HSH).

Los participantes en las entrevistas y grupos focales tenían diferentes características en lo que respecta a sus identidades y conductas sexuales: hombres *gay*, *trans* o *travestis* (Trav), trabajadores sexuales (TS), y jóvenes bisexuales llamados comúnmente *mostaceros* (sin identidad *gay* u homosexual, Most.)

El trabajo consistió en una revisión minuciosa de los datos provenientes de las transcripciones, la identificación de ejes temáticos, la elaboración de descripciones e interpretaciones, y la selección de citas pertienentes para sustentar e ilustrar los temas abordados. El objetivo de esta tarea fue analizar y evaluar, desde las narrativas, experiencias y perspectivas de los participantes:

- los conocimientos sobre el VPH y las VG;
- las actitudes y experiencias con respecto a las VG;
- la presencia de formas de estigmatización y discriminación asociadas a las VG;
- las prácticas de manejo de VG y la búsqueda de atención de salud;
- los conocimientos sobre las vacunas preventivas para el VPH;
- la aceptabilidad de estas vacunas;
- el posible impacto de la vacunación contra el VPH en las vidas, relaciones sociales y prácticas sexuales de los participantes y sus pares;
- la aceptabilidad de un futuro estudio de vacunas preventivas contra el VPH en hombres; y
- las opiniones y sugerencias de los participantes con respecto a las estrategias para reclutar voluntarios para un futuro estudio.

Luego de la presentación de los resultados del análisis, formulamos hacia el final de este informe una serie de conclusiones que sintetizan los hallazgos del estudio, seguidas de recomendaciones que pueden ser evaluadas y tomadas en cuenta en la planificación e implementación de actividades futuras de investigación y promoción de la salud relacionadas con el empleo de vacunas preventivas contra el VPH entre HSH en el Perú.

CAPÍTULO 1

**CONOCIMIENTOS SOBRE EL VPH Y LAS VG**

En general, el conocimiento sobre el VPH fue bastante limitado en todos los grupos. Solo unos pocos tenían alguna información básica o parcial al respecto, a veces relacionada con problemas de salud que afectan a las mujeres. En cuanto a las VG, encontramos que eran algo más conocidas, aunque solo por algunas personas, ya sea por que les parecía haberlas visto alguna vez en sus parejas, o porque habían oido hablar de ellas. No obstante, a algunos de los que creían haber visto VG les costaba diferenciar entre éstas y otras ITS u otros problemas de salud en la zona genital y anal.

**Conocimientos y nociones sobre el VPH**

De entre quienes tenían alguna información sobre el VPH, una persona refería haber oído que se presentaba con síntomas no muy “evidentes” (GF Gay), y alguien más señaló que “no tiene cura”, luego de ilustrar el caso de una prima suya. Otras dos personas señalaron que se podía transmitir por “contacto directo” entre una persona y otra (“piel a piel”), otra más mencionó que esto podía ocurrir aún usando condones, y hubo también quien vinculaba al VPH con la aparición de “brotes” y “ampollas” en las manos:

GF Gay: *Lo poquito que me acuerdo es que eran de “transición por pacto”, más que nada, o sea... a diferencia, por ejemplo, del VIH, los fluidos, el virus del papiloma humano era más por contacto; es lo que recuerdo yo de una charla de hace algún tiempo.* *(E: ¿Tienen idea ustedes de como se transmite?) Yo sabía que era eso, por contacto directo. [Otra persona:]* *Tenía entendido que es una de las enfermedades, perdón… es una de las ITS que podría transmitirse aún utilizando condón.*

GF Gay TS: *[Es un] virus que no tiene curación, es una enfermedad, bueno, tengo entendido que no tiene curación, tratamiento sí se... creo que se presenta a través de brotes en las manos, así como ampollita.*

GF Gay TS: *Tengo una prima que esta con papiloma pero le dan de alta… es como que crece unos bultitos… no sabe si es cáncer o es papiloma, pero la paran operando y eso para brotando... no tiene cura, dicen*.

Fuera de estos casos, había también quienes solo habían escuchado “el nombre” del VPH. Pero la mayoría no sabía de qué se trababa.

Solo dos personas (gay) reconocían que el VPH podía a afectar a hombres y mujeres. Uno señaló que se transmite por las relaciones sexuales “penetrativas”, y lo identificó como el agente causal del cáncer de cuello uterino en las mujeres (originado por “contagio” del hombre hacia la mujer), y de VG en hombres y mujeres:

ENT1 Gay TS: *... es un virus que les da a las mujeres en el útero... y que es malo para los bebitos. Y que le puede dar a los hombres también, aunque a veces hay errores de que solamente le da a las mujeres. (...) salen como verruguitas en… al costado… de los labios [vaginales] de la mujer, y en… y alrededor de la cabeza del pene del hombre. (E: ¿Sabes tú cómo se transmite este virus?) Mediante relaciones sexuales. (E: ¿De qué manera?) Penetrativas: pene – vagina, pene – ano...*

ENT2 Gay: *[Sabe]Que es una enfermedad que no tiene cura y que bueno si... la contrae un hombre y la contagia a una mujer crea un cáncer de cuello uterino... solo sé que aparece una pequeña... protuberancia, como un lunar, una carne en el pene o en algún lugar.*

También solo dos personas asociaron el VPH con las VG, en un caso por haber conocido a alguien que tenía VG:

ENT1 Gay TS: *verrugas son papiloma, lo mismo*.

GF Gay: *Yo tengo un amigo que le pasó, es... más o menos, un amigo cercano, eh… ¿creo que son como verrugas? Pequeñas, pedacitos de piel que salen. Algo así.*

En un grupo focal, una persona mencionó que el VPH sería “más manejable” y menos perjudicial que el VIH:

GF Gay: ... *el VPH creo que no es tan jodido como el VIH, en el sentido de que en verdad te friega el organismo por la misma enfermedad ¿no? Creo que el papiloma es un poquito más manejable.*

**Conocimientos y nociones sobre las verrugas genitales**

Las VG, en cambio, sí eran relativamente familiares, sobre todo para las travestis, y en menor medida para los hombres gay, mientras que para la gran mayoría de hombres sin identidad homosexual y con prácticas bisexuales (llamados comúnmente *mostaceros*) eran desconocidas, salvo por el hecho de que mucha gente puede reconocer a las verrugas en general, no específicamente las genitales (ENT14 Most: “yo lo tengo desde niño [refiriéndose a una verruga en su mano.]”)

Al ser consultados sobre las VG, solo unos pocos participantes tenían cierto conocimiento al respecto, y un par de personas se refirieron a ellas empleando el término “condilomas”. Entre varios otros, las VG eran asociadas frecuentemente con las “hemorroides”, y con la presencia de “granos”, “llagas”, “heridas”, y hasta “secreciones” (en la zona genital y en la boca). Una persona relacionaba la aparición de VG con el estrés, y otras dos con la existencia de “bultos” o la presencia de un “cáncer”:

GF Gay TS: *Son hemorroides…*

GF Gay TS: *Son como unas bolitas de agua, creo que salen a la altura de los genitales.*

GF Gay TS:... *las verrugas genitales es como… parece un hemorroide pero no es un hemorroide, porque el hemorroide este… la gente estreñida, y cuando tiene sexualmente con miembro muy grande también tiene pues una hemorroide, pero la verruga genital no sé… eso es un contagio*.

GF Gay TS: *Bulto tras bulto creo que es*.

GF Gay TS: *Parece una cresta de gallo.*

GF Trav TS: *Que yo sepa, las verrugas genitales son unas cosas que te cuelgan alrededor del pene… del glande, del pene... o del recto... como los condilomas que le llaman.*

ENT4 Gay: *yo tengo entendido que son como esos granos que te salen.*

ENT5 Gay: *le sale como unas llagas, creo*.

ENT9 Gay/Trav: *el papiloma humano... creo que es un virus de enfermedades venéreas ¿no? que… que te puede producir como llagas o secreciones de pus en parte de los genitales y… y anal o hacer el sexo oral puede salir en los labios...*

ENT13 Most: *[las VG] son como heriditas que te salen en el pene o en trasero, o como bultitos de carne*.

GF Trav TS: *... las verrugas creo que son llagas que te salen en contorno, puede ser en el recto, o de pene o la vagina que causa... ¿ardor y comezón?*

GF Trav TS: *... es rojo y produce un ardor, te da fiebre...*

GF Gay TS: *Esto es como un cáncer ¿no?*

[Comentario: Varias personas parecen estar asociando las VG con ITS supurativas o ulcerativas, como el herpes, gonorrea u otras.]

En relación con las formas de transmisión, quienes reportaban tener información sobre VG las reconocían usualmente como “peligrosas”, “muy contagiosas”, y *adquiridas* por contacto sexual. Una travesti, quien había tenido VG, desconocía cómo se transmiten (ENT7 Trav TS), otras señalaron que se transmitían probablemente “por sangre” o por la falta de “higiene” durante las relaciones sexuales, mientras que otras personas mencionaron la posibilidad de transmisión en relaciones sexuales que se realizan sin uso de condones:

ENT5 Gay: *... le sale como unas llagas creo, unas llagas, y si no le tratas a tiempo y no lo curas se puede infectar más*.

ENT9 Gay/trav: *[Se puede transmitir] a través de las relaciones sexuales sin protección, ni uso del condón*.

ENT13 Most: *se contagia cuando no usas condón*.

GF Trav TS: *...de repente no la hacen higiénicamente... pueden hacerlo así, con las manos sucias.*

Solo un entrevistado dijo saber que se trataba de una infección incurable (ENT2 Gay: “se queda en el cuerpo y no sale nunca”); y otro mencionó que podía ser peligrosa “a largo plazo” (ENT1 Gay TS).

En el caso de los llamados *mostaceros*, encontramos que dos de ellos vinculaban a las VG con el funcionamiento del sistema inmune, de dos formas distintas. Uno de ellos veía la presencia del VPH como la *causa* de una subsecuente disminución de las “defensas” del cuerpo (ENT12 Most: “... lo que sé es que se complica, que te baja las defensas”). Otro, en cambio, decía que las VG se pueden presentar como *consecuencia* de que haya decrecido el nivel de “defensas” del organismo (ENT13 Most: “[las VG] te salen cuando tus defensas están bajas”*.*) Este último, además, percibía que las VG afectaban más a los hombres homosexuales y a las mujeres:

ENT13 Most: *(E: ¿Sabes si estas verrugas salen más en hombres o en mujeres?) Eso no sé, yo he sabido que en los mariconcitos les sale, pero no sé en las mujeres. (E: Porqué crees que solo le sale a los “mariconcitos”... o las mujeres?) Ah, es que a veces sus defensas están bajas y se contagian. (E: Crees que a los chicos como tú, que suelen tener sexo con hombres y con mujeres, ¿también les podría aparecer estas verrugas genitales?) La verdad no lo sé, pero supongo que sí.*

Aunque unas pocas personas señalaron que las VG pueden “sangrar” y producir lesiones al “reventarse”, nadie mencionó espontáneamente la posibilidad de que tales lesiones y sangrado pudieran facilitar la transmisión del VIH. Una persona advertía que las VG pueden causar “daño” a futuro, y otra, solo luego de que se le consultara explícitamente sobre los peligros asociados a esas lesiones (incluyendo el VIH), reconoció que podían favorecer la transmisión del VIH:

ENT3 Gay: *... es una verruguita que… se produce alrededor del ano... que cuando se revienta sangra… y daña.*

ENT1 Gay TS: *(E: ¿Sabes si es peligroso infectarse con este virus?) A largo plazo sí puede ser peligroso, porque una persona puede… o sea, si las verrugas llegan a cortarse o a enredarse con un vello púbico... puede cortarse y pueden producirse más enfermedad, pues, infecciosas. (...) Están los dos muy conectados [el VPH y el VIH]... porque las verrugas se pueden… rasgar...*

De entre quienes decían saber algo de las VG, un par de personas pensaban que se curaban solas, sin necesidad de tratamiento (GF Gay TS: “yo creo que se cae solo”.)

Un joven gay que ofrecía servicios sexuales como travesti hizo una distinción que parece ser significativa para la manera en que las VG, y las ITS en general, pueden ser percibidas e interpretadas desde las experiencias y conocimientos locales de la gente. Esta distinción se refiere a un esquema en el que las ITS parecen ser clasificadas diferenciando a las que afectan internamente al cuerpo, “por dentro” [como el VIH, que no necesariamente genera signos exteriores visibles], en contraposición con aquellas ITS que se manifiestan externamente, “por fuera”, con signos reconocibles [como las VG y otras ITS], lo cual tendría implicancias para la forma en que se percibe la capacidad de los condones para ofrecer protección:

ENT9 Gay/trav TS: *Las verrugas genitales... entendí, creo que es una enfermedad también ¿no? de… de prevención sexual, pero solamente que se contagia por medio de… de tener intimidades, pero no (...) no es una enfermedad que se puede contagiar... o sea, por dentro, sino… sino es todo por fuera... por cualquier parte del cuerpo, porque no es una enfermedad que como las enfermedades venéreas que al usar el condón nos sabe proteger. (E: Entonces... el condón no siempre te va ayudar) No es seguro…*

Al respecto, un participante gay que reportaba tener VG hacía también una distinción similar, aunque en su caso él llegó a la conclusión de que esta ITS se podía transmitir “por contacto con la piel”, y que los condones no eran seguros para prevenirla. Él había inferido esto a raíz de la incertidumbre que le causaba el no saber cómo adquirió la infección, al no haber notado VG visibles en sus parejas antes de que le aparecieran a él mismo:

ENT11 Gay: *Sé que se contagian por las relaciones sexuales aunque uses condón, porque es por contacto con la piel, no necesitas incluso que se vean, porque en mi caso yo no recuerdo que ningún pata con el que estuve los haya tenido y de todas maneras me contagié... ¿quién me habrá contagiado...?*

**Fuentes de información sobre el VPH y las VG**

Entre algunos pocos hombres gay, el conocimiento sobre el VPH y las VG se derivaba de información que habían recibido, y en segundo término de situaciones que habían experimentado, ya sea por haber visto VG en sus parejas o amigos, o por haberlas tenido ellos mismos. Entre las travestis, varias reportaron que en sus reuniones sus pares solían referirse a las VG empleando nombres particulares, como “uvas” (el más común), “aretes”, o “mollejas”:

GR Trav TS: *Esos son los “aretitos” que tienen.*

GF Trav TS: *algunos dicen no solamente como verrugas, dicen como “mollejas.”*

GF Trav TS: *Algunos no sabían ¿Qué era eso? [las VG] (E: Así es, a veces los términos oficiales, clínicos, no se conocen...) No lo conocemos por su mismo nombre.*

GF Gay TS: *De travesti me levantan puros activos, y solo un caso, más bien un hombre... pero cuando me toco en la plaza de moderno, más vi las verdaderas “vendimias” que tenían ahí, las verdaderas “uvas.”*

Otras personas que reportaban saber “algo” sobre el VPH por información que habían recibido mencionaron que esta información les había llegado generalmente de manera informal, mediante comentarios de amigos o “rumores”:

ENT5 Gay: *... escuché rumores [sobre VPH] pero no, no sé detalladamente, o sea ¿qué es lo que significa? ¿qué es lo que pudiera causar?*

Quienes sabían sobre las VG por tenerlas o haberlas tenido anteriormente, recibieron información al respecto ya sea a través de “amigos”, luego de buscar en “internet” (ENT11 Gay), o al momento de recibir atención médica. Solo tres personas (ENT3, Gay; ENT10 Trav TS; ENT15 Most) mencionaron haber recibido información sobre las VG de parte de agentes vinculados a instituciones sanitarias, por ejemplo en “charlas” sobre ITS. Un joven bisexual refirió haber oído sobre el “papiloma” por un caso narrado, aparentemente, en algún medio masivo de comunicación:

ENT12 Most: *Lo que he escuchado es sobre un caso que se le presentó a una modelo brasileña que se le infectó generalmente todo el órgano, se le complicó y ese fue el caso que me sorprendió a mi, ahí fue donde conocí sobre ese tema.*

ENT15 Most: *[E: Quizá has oído hablar de las verrugas genitales.] De eso sí, en una charla que diste la vez pasada...*

CAPÍTULO 2

**EXPERIENCIAS Y ACTITUDES RELACIONADAS CON LAS VG**

**Experiencia de tener o haber tenido VG**

Solo cuatro entrevistados reportaron tener VG, o haberlas tenido alguna vez. Dos personas indicaron tener verrugas en otras partes del cuerpo (distintas de los genitales), y una de ellas quiso saber si esas verrugas eran causadas o no por el mismo virus que causa las VG. Por ejemplo, luego de saber (durante la entrevista) que diferentes tipos o subtipos del VPH pueden causar verrugas en distintas partes del cuerpo, una persona (gay TS) empezó a asociar algunas verrugas que él tiene (en el cuello) con la posibilidad de que sean causadas también por el VPH:

ENT1 Gay TS: *¿Entonces es lo que tengo en el cuello también son?, este… ¿hay algunas como colgaditos?... ¿lo que tienen las personas en el cuello? (E: Podría ser un tipo de papiloma, pero no necesariamente genital.) Son gordas y negras, así como… como lunares así colgaditos.*

Esta misma persona señaló que, aunque no le diagnosticaron una infección por VPH, reconoció en él mismo la presencia de VG (dos años atrás) cuando éstas fueron creciendo y se produjeron lesiones sangrantes y dolorosas por la fricción entre las verrugas y la ropa interior. Refiere también que, por el sangrado, en un primer momento asoció el malestar con algún problema en su “próstata”, pero luego de examinar más de cerca sus genitales comprobó que se trataba de VG:

ENT1 Gay TS: *(E: ¿Cómo te diste cuenta de que existían?) Cuando ya es grande, porque crecen. Se salen y… con el roce de la tela, de la ropa interior se… llegan a sangrar (...) Duele un montón. (...) Creí que era una… o sea, me salió en mi pene, ¿no?... pensé que era algo de mi próstata... y que era algo interno pues que sangraba y no me daba cuenta si no me dolía, peor era, pero la cruda realidad... lo vi muy cerca, eran verrugas genitales.*

[Esta persona era la que mostraba tener mayor información y conocimientos sobre el VPH y las VG.]

Una segunda persona (gay) manifestó haber tenido una VG hace aproximadamente un año y medio. Esta VG había aparecido en su glande, y la identificó cuando alcanzó el tamaño de un pequeño “lunar” de carne, con forma de “flor”. Como no tenía información previa acerca del VPH o las VG, su primera reacción fue de extrañeza, ante lo cual buscó ayuda médica:

ENT2 Gay: *Vi la… la verruga, como un… el tamaño de un lunar, una pequeña carne (...) como una florcita, pequeñita, diminuta… a la altura del glande. Inmediatamente fui al médico. (E: ¿Cómo te sentiste cuando la reconociste?) Cuando lo vi, pues me… me extrañé porque nunca había visto ese cuerpo… extraño en mi pene.*

Prefirió mantener en reserva su problema de salud, y no comentarlo con otras personas, fuera del personal de salud. Tampoco quiso comunicarlo a sus parejas sexuales (dos), para evitar sentirse mal o que sus parejas se alejen de él, aunque considera que una de sus parejas podría entenderlo mejor, ya que se trata de un médico:

ENT2 Gay: *No sé por qué, pero en realidad no, no se lo comenté a nadie...* *(...) Porque me siento mal. Me sentiría mal. Y como son personas que aprecio mucho, no quisiera que se alejen de mí por eso; aunque uno de ellos, bueno, no estoy con los dos al mismo tiempo, uno de ellos, el último es… médico, y creo que lo entendería mejor, pero no se ha dado el… el tema.*

La tercera persona que reportó haber tenido VG (travesti TS) señaló que, cuando notó la presencia de éstas, sintió “miedo” e incertidumbre por no saber qué podía haberle pasado. Como en el caso anterior, esto la motivó a buscar asistencia médica:

ENT7 Trav TS: *(E: Cuando te enteraste que tenías estas verrugas ¿Cuál fue tu reacción?) No sé, un poco de miedo ¿no? O qué haya pasado, por eso es lo que me fui al doctor ¿no? para que pase.*

Esta persona brindó también alguna información sobre las implicancias negativas de haber tenido VG, vinculadas principalmente con el aspecto funcional (problemas para defecar), y con lo desagradable (“feo”) e “incómodo” que resulta tener VG, tanto por las dificultades que esto representa al momento de tener relaciones sexuales, como por las consecuencias negativas para su autoimagen:

ENT7 Trav TS: *… [me] sentí incomodo cuando defecaba, me dolía cuando tenía relaciones, también me dolía. (…) Yo siento que es algo feo, para mí, no me gusta ¿no? y es algo incómodo.*

La cuarta y última persona que reportó haber tenido VG (gay) mencionó que se le presentaron en el ano hace un tiempo, que esto le generó un gran temor, y que no supo cómo actuar al respecto. La presencia de VG lo llevó a dejar de tener relaciones sexuales, tanto por la vergüenza como para evitar transmitir las VG a sus parejas:

ENT11 Gay: *[cuando le aparecieron las VG] ... me asusté mucho y no sabía qué hacer. Deje de tener relaciones porque me daba vergüenza y tenía miedo también contagiar a otras personas con eso.*

A diferencia de los casos anteriores, esta persona no buscó atención médica desde un inicio debido al sentimiento de “vergüenza” que le producía el tener VG, y solo recurrió a la ayuda de un amigo cuando las VG crecieron y comenzaron a incomodarle más, físicamente, por el dolor y por lo desagradable que le parecía (“era bien feo y molestoso”). Luego de que, alentada por este amigo, buscara atención médica, y una vez que le informaron que se trataba de VG causadas por el VPH, se alarmó ante la posibilidad de que fuera de una manifestación del Sida. Refiere finalmente que en el hospital le “quemaron” las VG y ya no las tiene más:

ENT11 Gay: *A mí me salieron unas verruguitas atrás y me daba un poco de vergüenza ir a verme, y crecieron mucho, y luego ya cuando me molestaba mucho le conté a un amigo y me dijo para ir a un hospital, y fui al hospital Loayza y ahí me dijeron que era el papiloma y me asusté mucho, porque yo había leído en internet que eso te da cuando tienes el Sida, y me hice mi prueba y menos mal no salió nada y... bueno, me atendieron y me quemaron las verrugas y se cayeron, ahora ya no tengo pero era bien feo y molestoso.*

Luego de recibir tratamiento, le ha quedado una cicatriz desagradable (“fea”) en el ano. Esto le avergüenza (“me da un poco de palta”) y ha afectado su vida sexual, ya que anteriormente le gustaba que le acaricien esa zona, pero ahora evita que se lo hagan luego de una mala experiencia que tuvo, cuando una pareja iba a hacerle sexo oral en el ano (“beso negro”) y notó su cicatriz. Después no volvió a ver a esta persona (implicando que se alejó de él debido a su cicatriz):

ENT11 Gay: *A mí antes me gustaba que jueguen los hombres con esa zona y ahora ya no pueden, uno me hizo sentir mal porque me preguntó “¿qué te ha pasado ahí?”, me iba a hacer “beso negro”, creo, pero luego se desanimó y eso me dio mucha palta... y nunca más lo volví a ver.*

En este caso, apreciamos cómo la presencia de VG puede tener importantes implicancias negativas en la autoimagen y el disfrute del sexo. El haber tenido VG significó para él un cambio significativo en su vida sexual, pues ahora prefiere que no le toquen el ano, e incluso ha cambiado su rol sexual: “hasta activo me volví, ja ja ja, porque no quería que me toquen por atrás.”

**Experiencias de haber visto VG en parejas u otras personas**

Pocas personas mencionaron haber reconocido la presencia de VG en parejas con las que habían tenido relaciones sexuales, o en sus amigos (tres entrevistados y algunos pocos participantes en grupos focales). Esto fue mencionado mayormente por quienes tenían experiencias en el comercio sexual. En la mayoría de los casos, notar o ver las VG les generó una sensación de miedo, incertidumbre y repulsión. Además, la presencia de VG en sus parejas o clientes solía generar situaciones embarazosas. Por un lado, entre las personas que vieron VG la reacción más común era la preocupación por la posibilidad de que les pudieran transmitir alguna enfermedad, seguida de una desconfianza hacia la pareja, sobre todo si se trataba de una pareja ocasional o alguien a quien conocieron recientemente. Por otra parte, mencionaron también la respuesta o reacción proveniente de aquellos que tenían estas VG, para quienes la situación se tornaba aún más embarazosa, por lo que usualmente trataban de negar que se tratara de VG, y ensayaban argumentos como, por ejemplo, el de que se trataba de “lunares” [de carne], o de “hemorroides”, lo cual puede ser visto como una “resistencia” a aceptar que uno tiene VG:

GF Gay TS: *… había un chico que me levantó, y es más, yo le eché por atrás y tenía una verruga ¡bien grande! (RISAS) Te lo juro, una verruga bien grande... “no, ese es lunar”, me dijo; oye, “y ese será el papilón? [pensó en ese momento].*

ENT1 Gay TS: *(E: ¿Cómo éstas personas se dieron cuenta de que eran verrugas?) Creo que no se daban cuenta, o creían que eran algo natural, como hemorroides. (...) Uno confunde eso, hemorroides con verrugas, o no quiere ver la cruda realidad que son este… papilomas ¿no? o verrugas genitales.*

Otros refirieron haber observado alguna vez una presencia anómala (fisiológica), como por ejemplo “carnosidades” o “bultos” en la zona genital de parejas sexuales que habían tenido en algún momento (ENT3 Gay*: “*una vez un punto [pareja ocasional] nomás vi que tenía tipo carnosidad en el ano.”) En estos casos, la reacción más común era la incertidumbre por no saber de qué se trataba, junto a la preocupación por la posibilidad de adquirir alguna enfermedad:

Por lo general, estas personas reconocieron recién en el contexto de la entrevista o grupo focal que lo que habían visto en sus experiencias sexuales podían ser VG. Un gay *deschavado* (fuera del “closet”), por ejemplo, ofreció una descripción detallada de una ocasión en la que pudo haber estado ante una posible VG. Esta persona, luego de revisar nuevamente las imágenes que le mostró el entrevistador, pareció reconocer y recordar un caso en el que tuvo relaciones sexuales con un hombre que presentaba unas “carnosidades” en su pene (antes había dicho que no había visto VG en sus parejas). En ese caso, luego de una reacción inicial de sorpresa, decidió proseguir la interacción erótica mediante la masturbación, evitando “roces” con los genitales de su pareja ocasional por “miedo” a adquirir alguna enfermedad:

ENT8 Gay Desch: *... algunas heridas sí he visto pero esta herida de acá [de una fotografía], sí, en algún muchacho… (E: Carnosidades…) Ya! Ésta!, le he visto varios en esta parte de acá, o sea, cuando le pelas el pene ¿no es cierto? Acá en este borde, ahí le he visto tres, cuatro, que yo agarré y me iba a ir de boca y dije “¡oe, oe!” Porque yo siempre cuando voy a hacer el “güagüis”, el sexo oral, doctor, siempre lo manoseo... lo succiono ¿no?, pero como ese chico era tan aventajado, ¡tan chala!, tan grueso pues, yo agarré y le saqué… y ya me saqué doctor, y con él me empecé a masturbar ¿me entiende?… manosearnos, pero no permití que el chico… me rozara porque tenía miedo doctor.*

Pero más allá de que la presencia de lesiones o VG sea un tema embarazoso en la intimidad, sobre todo con parejas ocasionales, también es posible que el hecho mismo de abordar el asunto pueda conducir a reacciones impredecibles, e incluso violentas, de parte de quien podría estar teniendo VG u otras ITS. La expectativa de posibles reacciones violentas, desagradables, embarazosas o imprevistas serían entonces elementos que limitarían la posibilidad de tratar el tema. En el caso de esta última persona, por ejemplo, vemos que en aquella oportunidad sintió temor. No obstante, prefirió no tratar el tema con esa pareja, debido a una mala experiencia que tuvo anteriormente con otro hombre al que le hizo notar que éste tenía una lesión en sus genitales (aparentemente una ITS). En esa ocasión este hombre reaccionó violentamente cuando se vio increpado por la posible ITS:

ENT8 Gay Desch: *(E: Pero ¿no hablaron acerca de lo que él tenía?) No le hablé, para que no… no le hablé en el momento porque una vez le hablé a un chico [otro], “oye, mira ve, ¿qué es lo que tienes? ¿qué esto?”, y ¡pla! Me dio un tandón, doctor, en el hotel y salí perdiendo yo, me quedé ensangrada, “¡oe, qué tienes!”; yo le dije “esas enfermedades son venéreas”, que es el Sida, o sea, mi ignorancia mía fue hace cinco años, cuando a un chico le dije “tú tienes esto, esto” y ¡pum! Me dio un tandón porque lo señalé, lo acusé que tenía eso... por eso que no converso con él.*

Otras personas (travestis TS), mencionaron haber tenido reacciones de rechazo ante la presencia de posibles VG (“carnosidad blanca”, “condilomas”) en sus clientes. En estos casos no se llegó al nivel de la violencia física, pero sí se produjeron situaciones tensas y de conflicto, motivadas por las actitudes y conductas “sospechosas” de sus clientes, es decir, lo que parecen ser estrategias empleadas por quienes estarían teniendo VG para prevenir que otras personas las noten al momento de tener relaciones sexuales: principalmente, evitar que la pareja observe los genitales, y tener relaciones sexuales a oscuras. Estas estrategias, no obstante, pueden generar una sensación de incertidumbre y desconfianza por las posibles intenciones de la pareja o cliente. Finalmente, tales situaciones pueden llegar a resolverse con la interrupción de las relaciones sexuales:

ENT10 Trav TS: *[Un cliente] se desnudó, abrí el preservativo, dijo: “¡no! sin preservativo”, “no -le digo- yo me tengo que cuidar, yo no me puedo arriesgar a nada.” “Sí, pero ahorita todavía no eh…” –le respondió el cliente-. ¡Pá! Me apagó la luz. Pero después yo le prendo la luz, yo le puse el preservativo, aun ahí no me había dado cuenta de lo que tenía ¿no?... Usted dirá que cuando le puse me pude haber dado cuenta, pero no me di cuenta. Entonces, presentía yo que algo no andaba bien, prendí la luz y él… la persona se había retirado el preservativo. Cuando yo me amargué obviamente, cuando él se retiró yo observé bien, o sea, él estaba echado y yo le vi un… una carnosidad a la altura de los testículos, como una carnosidad blanca, parecía una ampolla, yo no sé si eso será papiloma pero fue algo… una carnosidad blanca, grande; entonces yo le pregunté “¿Qué es lo que tienes ahí?” “No -me dijo-, es una quemadura.” “Eso no es una quemadura -le digo-, una quemadura no se hace así.” Es una carnosidad, es carne lo que se le había formado. Inmediatamente lo boté. Lo boté y me cambié y me salí un poco como que… como que no, no me agradó mucho. Supuse que no era algo normal.*

GF Trav TS: *[En una ocasión] llegó un taxista, ya… y me llevó a una cochera (...) y cuando yo le puse condón, él... no le quería pelar el glande. O sea, esa cosa ¿no?, su cuellito, quería que lo meta así... “Qué raro”, yo decía “¿Por qué?” Porque a todos los hombres le encanta… pelado… (...) Yo le dije “¡ay, prende la luz!”, no quería prender tampoco la luz. “¿Oye, qué te pasa? –le digo– ¿Por qué no?”, “no, no, así no más”, me dice (...) “¿No me das una mamada?”, me dice, “¡no, no, no!”, le digo... “yo no tengo confianza, no veo algo, no me brindas esa confianza para yo hacerte como tú quieres”, le digo (...) Me pongo así en pose... en pose de perro y no sé, eh… siento que hace así, como si estuviera sacándose el condón, ¿me entiendes?, entonces como yo soy más rata prendo la luz y le hago así y le pelo ¡sá! Y tenía condilomas… Y quería que le devuelva sus 20 soles, no le devolví.*

Pero las reacciones no siempre ni necesariamente se orientan al rechazo. Una travesti TS, por ejemplo, luego de recibir información sobre las VG durante la entrevista, manifestó que notó tales VG en el ano de uno de sus clientes, quien deseaba ser penetrado. En esa ocasión, esta travesti había asociado esas verrugas con las “hemorroides”, y no le parecieron un motivo de mayor preocupación en ese momento, en que penetró a su cliente usando un condón:

ENT6 Trav TS: *A veces… como yo trabajo en la calle y a veces nos tocan que son modernos, o a veces son “ollas”, que le dicen: que les gusta que le penetren. Y cuando yo he visto a uno que tiene este… ¿cómo se dice?... papiloma, un chico cuando le penetré tenía pero chiquitita, una, dos, en el ano. Bueno, lo penetré todo con condón ¿no? y no sabía qué es… qué era eso, sino pensaba que era una hemorroides.*

En las ocasiones narradas durante los grupos focales apreciamos igualmente reacciones de rechazo y desagrado, e incluso “asco”, ante la aparente presencia de VG y hacia quienes las estarían teniendo. Estas respuestas venían comúnmente vinculadas al desconocimiento, y notamos también aquí la asociación que varias personas hacen entre las VG y las hemorroides. Por otro lado, la presencia de VG llevó también a algunos a perder el “apetito sexual” en esos momentos:

GF Gay: *Lo he visto en algún ocasional [pareja ocasional]... se puede decir. Sí, y me acuerdo que era como… no sé si sería eso, pero sí he visto que eran como verruguitas en el ano; y ahí dije: “ahí no me acerco.”*

*GF Gay: Yo una vez salí con un chico que al momento de palparlo… o sea, meterle el dedo, noté que habían como que bultitos. Y dije “bueno, de repente es este…”, no, no, ¡no!… cuando se hinchan las venas, “hemorroides”. Dije: “tienes una hemorroide”, ¿por qué?, no sé… dije “de repente es eso.” Ni idea.*

GF Trav TS: *Sí he visto una verruga pero lo he visto como una carnecita... una carnecita en el cuerpo, en la parte que es sensible, que puede ser en la vagina o en el pene en el varón o en la mujer.*

GF Gay TS: *... ahí por la plaza ¿no? me levanté un pata… estaba rico. (...) Mira y mira yo estaba, así, agarrando así y tenía una verruga por acá y agarré... yo no lo miré, lo agarré y sentí, y sentí algo feo. (E: ¿Qué sentiste?) Así como un lunar. (E: Ya, ¿un lunar de carne?) Sí, yo le dije ¿Qué tienes acá?, se avergonzó; no es normal, ¿no? Y... pero estaba así, como un lunar así grueso, un lunar de carne que se levanta… Así estaba, y ya me quitó todas las ganas, y era joven todavía... para aprovechar... (...) A mí me da asco, me dio asco, el pata estaba bien rico, después que le toqué eso ¡Aj! Me dio asco, se me quitó todas las ganas.*

GF Gay TS: *Da mucho temor, sobre todo te quita el apetito sexual.*

**Sin experiencia de haber tenido o visto VG**

Fuera de los pocos casos narrados líneas arriba, la mayoría manifestó no haber visto o reconocido VG en ellos mismos, en sus parejas sexuales, o entre sus amigos, independientemente de si tenían información sobre VPH o VG, o de si no la tenían, siendo esto último lo más común. Al respecto, ninguno de los jóvenes llamados *mostaceros* señaló haber visto alguna vez una VG, mientras que solo algunas personas de los otros grupos refirieron que sabían de casos de terceras personas que tenían VG, ya sea amigos, conocidos, o gente de la que habían oído decir que tenían VG. Se habló también, nuevamente, de cómo las VG son confundidas a veces con las “hemorroides”, y se asoció incluso la aparición de VG con la falta de “aseo”:

GF Gay*: ... un amigo muy cercano a mí me contó que le había salido [VG] en las aletas de las nalgas. Y que le habían como que… quemado, o algo así. Pero no sé si hoy en día él lleva un tipo de tratamiento.*

ENT9 Gay/Trav TS: *Amigas que sí, comentan así ¿no? como hace poco que tenemos, que debido a una reunión ¿no? y así, justamente di mi consulta pero solamente, como digo, son amigas que me comentaron ¿no?, justamente amigas comentan...*

GF Gay TS: *Mucho depende del aseo.*

ENT11 Gay: *... no he visto en nadie, he escuchado pero creo que las confunden con las hemorroides, las uvas, y les he dicho que eso no es.*

ENT13 Most: *(E: ¿Has conocido a alguien con alguna verruga genital?) He escuchado, pero no he visto, una vez un “man” me contó que le vio unas bolitas en el culo a un mariconcito y que no quiso tirárselo, y solo le dejó que le se la chupe. (E: ¿Te dijo que sintió al verla?) Me dijo que le dio asco, pero que no le hizo nada por ahí [no lo penetró por el ano]. (...) Yo tenía un amigo que nos contaba que tenía una heridota en su cabeza del pene, y nos decía que eso era normal porque es como su bautizo por meterse con un “cabrito”.*

En general, a las personas de este grupo que no tenían experiencia de haber tenido o visto VG les resultaba desagradable pensar en la posibilidad de tenerlas en el futuro. Esto resultó aún más marcado entre quienes habían tenido la oportunidad de observar VG en imágenes fotográficas en el contexto de la entrevista, grupo focal, o en charlas informativas previas; y algunos manifestaron abiertamente el miedo que les generaba observar aquellas imágenes, que a varios les parecieron “chocantes”:

ENT 15 Most: *... en una charla que diste la vez pasada me acuerdo que hablaste de las verrugas genitales y las fotos que estaban ahí eran bien malogradas, ja ja ja ja. (...) Chuma! Que feo!, sí se ven bien malandras en esas fotos, yo nunca he tenido eso.*

ENT8 Gay Desch: *... ahorita que yo veo ante… por la pantalla [imágenes de VG] y la verdad que me siento un poquito mal, eh… un poco es incómodo... (...) La verdad, viendo bien la foto, me siento un poco tenso... porque a veces pensamos de que hay muchachos que dicen “yo ajusto”… pero cuando uno a veces va de frente a penetrar a veces el muchacho agarra y dice... yo le digo “a ver, pa’ ver”, y a veces uno quiere, la verdad, irse hasta de lengua y de boca pero no permiten... y me palteo, la verdad no me siento un poco... me asusto al ver esas heridas [en las imágenes], como en las nalgas he visto costras... cuando yo he sabido probar... sobar, he agarrado y he sabido sacar las costras y ha sangrado esas costras y me palteo ¿no?*

GF Gay: *... francamente las fotos que nos mostraron, la verdad que apestan.*

GF Gay: *... para llegar a ese estado [en una fotografía] la persona ha tenido que descuidarse un montón.*

Unas pocas personas reportaron haber reconocido en su cuerpo o en el de otras personas signos de otras ITS. Uno de ellos, por ejemplo, no había percibido en él mismo la aparición de VG, pero al revisarse sí había notado lesiones que, en un principio, atribuía al “calor”, pero que ahora piensa podrían estar asociadas con el herpes:

ENT5 Gay: *... revisé mi cuerpo pero no, no [encontró VG]; lo que sé me llegó a salir son como esas heriditas que Ud. me dice… del Herpes (...) pensaba que era una herida por el calor.*

Y otra persona (gay) manifestó haber identificado otras ITS en sus parejas, por la presencia de “llagas”, “costras”, o heridas sangrantes en la zona anal o en el pene, cuando ha acariciado o tocado los genitales de sus parejas. En esas ocasiones se ha sentido perturbado y temeroso (“palteado”), y ha preferido usar condones o masturbarse:

ENT8 Gay Desch: *... siempre salgo con muchachos (...) en algunas oportunidades sí he podido tocar, cuando he manoseado chicos, he podido tocar heridas en la parte rectal, puede ser como… ha sido también adentro del recto, como afuera en la… en la nalga; he podido notar heridas pero yo siempre me he manoseado y me he palteado, más bien discúlpame la expresión, he podido ver este… como heriditas en la parte rectal, ¿qué le puedo decir? este… como llagas en la nalga, pero en la parte de adentro he visto como chupitos, algo parecido así, ¿me entiendes?... bolitas… y yo le decía: “Oye, ¡qué es esto!”... me dicen: “Ah no, es una herida que me ha salido”, y yo le digo: “ten cuidado, mejor”, y ya, ¿me entiendes? Así yo me pongo el preservativo con ese chico cuando me pedía que yo le haga el sexo, y yo la verdad me palteaba y prefería... la verdad, masturbarme, o decirle “tú mismo eres”, o la verdad me ponía de acuerdo con el chico, le dije: “Los dos nos masturbamos porque la verdad que me palteo.”*

Este tipo de “revisiones” eran más comunes entre las travestis dedicadas al comercio sexual. Una de ellas, por ejemplo, afirmaba que se “palpaba” al momento de realizarse “lavados” antes de salir a ofrecer servicios sexuales:

ENT6 Trav TS: *... cuando tú te haces el lavado para que… sale a trabajar… siempre se tiene que pasar la mano y eso se siente ¿no? [pero no encontró VG]*

**Vergüenza, silencio, estigma y discriminación**

En general, las VG no suelen ser un tema de conversación. Entre quienes han tenido o tienen VG, encontramos que por lo común tampoco se habla de esto en el contexto de las relaciones de pareja, ni tampoco con los clientes que solicitan servicios sexuales. Solo una de las personas que señalaba haber tenido VG mencionó que se lo había comunicado a su pareja (ENT7 Trav TS, aunque no dijo cuál había sido la reacción). Un hombre gay (TS), por ejemplo, mencionó que ninguna de sus parejas sexuales le ha confiado que tiene o ha tenido VG, ni tampoco él le ha mencionado a alguna de sus parejas que él mismo había tenido VG (extirpadas):

ENT1 Gay TS: *(E: ¿Tú les sueles contar a tus parejas sexuales sobre tu infección con el papiloma?) No. (E: No, ¿Por qué?) Porque… no sé, no les cuento pues.*

Para explicar este silencio alrededor del tema de las VG, varias personas emplearon los conceptos de vergüenza y “recelo”:

ENT4 Gay: *Creo que eso nadie lo cuenta*.

GF Gay TS: *Pocas veces te lo comentan porque se avergüenzan.*

ENT3 Gay: *Conozco varios amigos, a varias amigas que le fastidian por eso ¿no? pero nunca le he... le he visto, nunca le he examinado, tampoco le… me han dicho ¿no? por su propio tipo; es un recelo ¿no?*

Siendo que la presencia de VG puede ser un motivo de estigmatización, no sorprende que muchas de las personas hayan enfatizado que no tenían VG o que nunca las habían tenido. Una persona, por ejemplo, al ser consultada sobre si había tenido alguna vez VG, respondió “claro que no” (ENT4 Gay), aparentemente implicando en su respuesta que la misma pregunta sería algo impropia.

En este marco, la “vergüenza” y el “recelo” estarían operando de varias formas. Por un lado, el hecho de que una persona tenga VG puede llevar a que se le estigmatice. Como las personas que tienen o han tenido VG anticipan que pueden ser rechazados, prefieren no hablar del tema o tratan de ocultar sus VG al momento de tener relaciones sexuales. Hemos visto ya cómo algunas personas prefieren tenerlas a oscuras, o tratan de mantener cubierto el glande con la piel del prepucio, o argumentan que lo que tienen no son VG, sino “quemaduras”, “cicatrices”, o “hemorroides”, para evitar ser estigmatizadas.

Por otra parte, estos comportamientos adoptados para ocultar las VG encuentran su contraparte en las actitudes sociales de rechazo, burla u hostigamiento (“le fastidian”), reales o esperadas, hacia quienes tienen o se piensa que tienen VG, lo cual estaría incluso obstaculizando la búsqueda de apoyo o asistencia médica:

ENT11 Gay: *A mí me salieron unas verruguitas atrás y me daba un poco de vergüenza ir a verme [al médico] y crecieron mucho...*

ENT10 Trav TS: ... *él [su cliente] estaba echado y yo le vi … una carnosidad a la altura de los testículos, como una carnosidad blanca, parecía una ampolla, yo no sé si eso será papiloma pero fue algo… una carnosidad blanca, grande; entonces yo le pregunté “¿Qué es lo que tienes ahí?” “No -me dijo-, es una quemadura.” “Eso no es una quemadura -le digo-, una quemadura no se hace así.” (...) Inmediatamente lo boté. Lo boté y me cambié y me salí un poco como que… como que no, no me agradó mucho. Supuse que no era algo normal.*

GF Gay TS: *... ahí por la plaza ¿no? me levanté un pata… estaba rico, está bien (RISAS), mira y mira yo estaba, así, agarrando así y tenía una verruga por acá y agarré, yo no, yo no lo mire, lo agarre y sentí, y sentí algo feo. (E: ¿Qué sentiste?) Así como un lunar. (E: Ya, ¿un lunar de carne?) Sí, yo le dije ¿Qué tienes acá?, se avergonzó; no es normal, ¿no? Y... pero estaba así, como un lunar así grueso, un lunar de carne que se levanta… Así estaba, y ya me quitó todas las ganas, y era joven todavía... para aprovechar...*

GF Gay TS: *La mayoría de las personas se avergüenzan, no hablan de su problema… [para evitar que] se burlen, o muchas veces no van a los hospitales por vergüenza…*

GF Gay TS: *[Quienes tienen VG no hablan de eso] Por rechazo de la gente.*

Sin embargo, las travestis aparecen aquí como la excepción, pues en este grupo se mencionó en algunas oportunidades que sí habían oído hablar acerca de las VG en sus grupos de pares, mayormente cuando viven juntas y tienen ocasiones para conversar sobre sus experiencias con sus clientes o sobre sus propios problemas de salud, o cuando andan desnudas y se pueden observar unas a otras.

ENT6 Trav TS: *Unas cuando ven a un cliente dicen “ah, ese punto tiene una verruga en el pene.”*

ENT9 Gay/Trav TS: *Amigas que sí, comentan así ¿no? como hace poco que tenemos, que debido a una reunión ¿no? y así, justamente dí mi consulta pero solamente, como digo, son amigas que me comentaron ¿no?, justamente, amigas comentan...*

Resulta interesante apreciar cómo, entre las travestis, el tema podía ser tratado con una actitud algo distinta, a veces en un ambiente lúdico, en el que podían referirse a las VG empleando “apodos” (“uvas”, “aretes”). No obstante, también en este entorno encontramos el argumento de las “hemorroides”, empleado para ocultar que se tiene VG:

ENT6 Trav TS: *Entre amigas así que hay algunas que sí... este, como te digo, “hemorroides”, se dicen, porque en sí no las he visto; se dicen “Ay, me salió una uvita, que la hemorroides, que esto, que otro”, y hay algunas que dicen que sangran por el ano ¿no? (E: ¿Cómo te lo contaron? ¿Asustadas, con vergüenza?) No, riéndose… en son de broma, porque como yo vivía con dos o tres amigas en un solo cuarto... entre nosotras nos contábamos y como parábamos desnudas “Y tú tienes una… [risas]”, entre nosotras le poníamos el apodo de “arete”. “Ay, tú tienes un arete ahí”... pero dicen que eran… no me decían si eran verrugas, o tampoco las veía, pero decían que eran “hemorroides”. Que a la hora de defecar, a veces sangran.*

[Comentario/interpretación:] Como vemos, las “hemorroides” aparecen como un tema recurrente en las narrativas de los participantes en el estudio, lo cual merece un comentario adicional. Tenemos, entonces, el problema de cómo se presenta a las personas la distinción entre “hemorroides” y VG. Encontramos, en primer lugar, que hay quienes parecen confundirlas. En efecto, es posible que en ciertos casos la identificación entre hemorroides y VG se deba a confusiones, al desconocimiento sobre el VPH, y a la mayor popularidad de las hemorroides. Como la palabra “hemorroides” está en el imaginario colectivo asociada siempre a problemas de salud en el ano, si una persona nota en el ano de alguien más la presencia de un signo anormal (que podría ser una VG), y si no conoce o nunca ha visto realmente una hemorroides o una VG, entonces sería relativamente fácil que asocie ese signo con las “hemorroides”.

Pero en otros casos, como algunos de los narrados por varios participantes, la idea de la presencia de hemorroides podría ser empleada de manera instrumental por quienes tienen VG para evitar que otras personas vinculen la presencia de tales signos fisiológicos anormales con infecciones. Esto sugiere que, desde las concepciones de la gente, sería muy distinto tener una enfermedad que simplemente aparece sin una voluntad de por medio (como las hemorroides: “me salió una uvita, que la hemorroides...”), y tener una infección que se adquiere (como la infección por el VPH), que puede ocurrir como una consecuencia de las prácticas sexuales, y que sería motivo de vergüenza o estigmatización. En estas situaciones, reconocer que se tiene una infección *adquirida* haría posible que otras personas infieran las posibles causas (asociadas con la conducta sexual), con lo que podrían cuestionar la moralidad o las prácticas sexuales de quien tiene la infección. Pero en el primer caso, de enfermedades que simplemente *aparecen*, sería más difícil que se culpe o se juzgue a quien la padece. De ahí el empleo del argumento de las “hemorroides” como una forma de ocultar que se tiene VG. Por ejemplo, una persona que había tenido VG, al referirse a aquellas otras que decían tener “hemorroides”, señalaba:

ENT1 Gay TS: *Creo que no se daban cuenta, o creían que eran algo natural, como hemorroides. (...) Uno confunde eso, hemorroides con verrugas, o no quiere ver la cruda realidad que son este… papilomas ¿no? o verrugas genitales.*

La misma lógica parece estar operando detrás de los otros argumentos comunes empleados por quienes tienen VG para alegar que no las tienen, como cuando se habla de “cicatrices”, “lunares” o “quemaduras”, que comparten la característica de ser presencias ya sea naturales o causadas por eventos que están fuera de la propia voluntad o que no tendrían relación con los comportamientos sexuales:

ENT10 Trav TS: *... yo observé bien, o sea, él [su cliente] estaba echado y yo le vi un… una carnosidad a la altura de los testículos, como una carnosidad blanca, parecía una ampolla, yo no sé si eso será papiloma pero fue algo… una carnosidad blanca, grande; entonces yo le pregunté “¿Qué es lo que tienes ahí?” “No -me dijo-, es una quemadura.” “Eso no es una quemadura -le digo-, una quemadura no se hace así.”*

Ahora bien, como señalábamos anteriormente, en un contexto de desigualdades de poder entre los miembros de la pareja, el carácter embarazoso de las situaciones en que una persona identifica la presencia de alguna ITS en su pareja puede, en ciertos casos, resolverse en respuestas violentas, ya sea que se trate o no de VG:

ENT8 Gay Desch: *(E: Pero ¿no hablaron acerca de lo que él tenía?) [aparentemente VG] No le hablé, para que no… no le hable en el momento porque una vez le hablé a un chico [otro], “oye, mira ve, ¿qué es lo que tienes? ¿qué esto?”, y ¡pla! Me dio un tandón, doctor, en el hotel y salí perdiendo yo, me quedé ensangrada, “¡oe, qué tienes!”; yo le dije “esas enfermedades son venéreas”, que es el Sida, o sea, mi ignorancia mía fue hace cinco años, cuando a un chico le dije “tú tienes esto, esto” y ¡pum! Me dio un tandón porque lo señalé, lo acusé que tenía eso... por eso que no converso con él, después sí... (...) Usted sabe porque a veces nos sentimos... yo me siento al menos un… no me siento tampoco, tampoco una mujer ¿no? pero me siento una femenina en el momento cuando estoy por momento con mi ropa en la cama, porque se da por ponerme mi calzón, mi… mis cosas ¿me entiendes?*

Por otra parte, encontramos también que, desde la perspectiva de dos jóvenes del grupo conocido como *mostaceros* (ninguno de los cuales afirmó haber observado VG), las VG eran vistas ya sea como algo que le ocurría a los hombres homosexuales, o a las personas que tienen Sida:

ENT13 Most: *(E: ¿Sabes si estas verrugas salen más en hombres o en mujeres?) Eso no sé, yo he sabido que en los mariconcitos les sale, pero no sé en las mujeres. (E: Porqué crees que solo le sale a los “mariconcitos”... o las mujeres?) Ah, es que a veces sus defensas están bajas y se contagian. (E: Crees que a los chicos como tú, que suelen tener sexo con hombres y con mujeres, ¿también les podría aparecer estas verrugas genitales?) La verdad no lo sé, pero supongo que sí.*

ENT15 Most: *Pero eso [las VG] ¿le sale a los que tienen el SIDA? (E: No solo a ellos, aunque es mas probable que cuando una persona tiene bajas defensas a causa del VIH pueda aumentar la aparición de las verrugas) Chuma! Qué feo, sí se ven bien malandras en esas fotos, yo nunca he tenido eso. (E:Pero ¿tú sabes como se transmite?) Por el sexo pes, los “carretillas” las tienen. (E:¿Que son las “carretillas”?) Los cabritos pe!.*

**Preocupaciones ante la posible adquisición de VPH-VG**

Al revisar las actitudes hacia el VPH y las VG, identificamos algunas preocupaciones manifestadas sobre todo por aquellos que no habían tenido VG y/o no manejaban mayor información al respecto. Más allá de las reacciones de rechazo e incertidumbre, el hecho mismo de abordar estos temas generó muchas preguntas de parte de los entrevistados, y principalmente entre los participantes en grupos focales. Las preguntas se referían, por lo general, a temas como: la forma de transmisión del VPH-VG, las poblaciones o grupos más expuestos, cómo reconocer o identificar las VG, de qué formas se manifiesta, cómo se puede tratar, y qué consecuencias para la salud puede acarrear el hecho de adquirir el VPH o tener VG. En la sección final (“Recomendaciones”) hemos desarrollado más en detalle estas preguntas, por lo que nos limitaremos aquí a mencionar estas preocupaciones con referencia a algunos aspectos que fueron apareciendo en las narrativas de los participantes.

Un primer tema se refiere a cómo son percibidos los problemas o probables problemas derivados del hecho de tener VG. Sobre este punto, resaltó la importancia que algunos otorgaban al impacto de las VG en el aspecto funcional del organismo. Al respecto, hubo quienes hablaban, por ejemplo, de cómo y cuánto afectaban las VG a la capacidad para orinar, o defecar (ENT7 Trav TS: “[me] sentí incomodo cuando defecaba, me dolía”). Así, una persona gay con experiencia en el comercio sexual manifestó sus dudas respecto de si podría llegar a tener dificultades para orinar si las VG se presentaban también al interior de la uretra:

ENT1 Gay TS: *Yo me imagino que dentro de la uretra deben crecer más papilomas ¿no? más… más verruguitas, y eso debe dificultar el paso de la orina.*

Esta persona mostró también su preocupación por un asunto que mencionaron también otros, el del impacto de la presencia de VG en el desempeño sexual:

ENT1 Gay TS: *(E: ¿Has encontrado [en] algunas personas verrugas en la zona anal o en el pene?) En algún tipo habré visto o he sentido ¿no?... que no entraba el pene y que era por una verruga… grande.*

ENT7 Trav TS: *... cuando tenía relaciones [sexuales] también me dolía.*

Como observamos entre aquellos entrevistados que habían tenido VG, este tema de la sexualidad estuvo bastante ligado a problemas de bienestar emocional y a la autoimagen, principalmente por el miedo al rechazo, o por haberlo experimentado a causa de las VG:

ENT7 Trav TS: *... yo siento que es algo feo, para mí, no me gusta ¿no? y es algo incómodo.*

Un cuarto e importante ámbito de preocupación se refiere a la posibilidad de transmitir el VPH o las VG a las parejas sexuales:

ENT1 Gay TS: *[Si uno tuviera VG] a la otra persona [la pareja] le puede afectar mucho.*

ENT5 Gay: *... si no le tratas a tiempo y no lo curas se puede infectar más, más y ya después puede ser… puede contagiar ¿no? a otra persona*.

GF Trav TS: *... es peligroso porque... podría haber sangrado y esta sangre que contiene el virus podría contagiarte con otra persona*.

ENT11 Gay: *[cuando le aparecieron las VG] ... me asusté mucho y no sabía qué hacer. Dejé de tener relaciones porque me daba vergüenza y tenía miedo también contagiar a otras personas con eso.*

Finalmente, encontramos que la posibilidad de que se desarrolle un cáncer anal, o que se llegue a adquirir el VIH por tener la infección por el VPH no era concebido como una preocupación visible entre los participantes, salvo en un grupo focal, en el que algunas personas (gay) hicieron preguntas al respecto, aunque no espontáneamente, sino luego de que se les ofreciera una explicación sobre el VPH, incluyendo el tema del cáncer anal:

GF Gay: *Pero básicamente ¿Qué es el riesgo? ¿cáncer anal? O sea, ¿no tendría otra consecuencia, una ITS mucho más grave?* *[Otra persona:] Es básicamente, el peligro del cáncer anal. (E: Sí) Digamos... no es que, por ejemplo, digo cáncer al pene o… otra cosa; básicamente ¿el peligro es el cáncer anal?*

CAPÍTULO 3

**MANEJO DE VG Y ATENCIÓN DE SALUD**

Entre aquellas pocas personas que reportaban tener o haber tenido VG, así como en los casos narrados por quienes tenían referencias de experiencias ajenas, la presencia de tales VG solía ser motivo de respuestas o acciones relacionadas con el manejo del problema y/o la búsqueda de asistencia médica. Podemos abordar este asunto distinguiendo tres aspectos: las prácticas individuales y sociales adoptadas por las personas para manejar la presencia de VG, la búsqueda de apoyo sanitario, y las percepciones y experiencias de las personas (con o sin historia de VG) con respecto a los servicios de salud para ITS.

**Prácticas asociadas al manejo de VG**

Ante la presencia de VG, una reacción común suele ser la extirpación *artesanal*. Para ello, se puede recurrir a diferentes técnicas, que se ponen en práctica generalmente de manera individual, aunque entre las travestis encontramos alguna referencia a amigos que estarían también ayudando en estos procedimientos “caseros” de extracción de VG. Así, una travesti, por ejemplo, mencionó haber oído decir que hay quienes amarran sus VG con vellos púbicos con la finalidad de cortar o extraer sus VG (GF Trav TS.) Otra, por su parte, señaló que ella misma le había cortado lo que parecían ser VG a una amiga, empleando unas tijeras, y hay quienes indicaron también que esto podía hacerse “con la mano”:

GF Trav TS: *... no sé si habrá sido verruga, no sé, yo tenía una niñita [travesti más joven], no sé su nombre pero yo le he cortado con la tijera. No sé si es, bueno, pero de él… estaba prendido en su ano.*

GF Trav TS: *Una parte así, chiquitita no más era, pero se movía como una lombricita. Creo que… creo que él cortó con su mano, creo… más el pene, porque la sangre…*

GF Trav TS: *Igual yo le he cortado pero ha salido bastante sangre, lo he tapado con algodón... yo le he cortado, pero no le he cortado todo pues, si no estaría creciendo, no lo sé.*

No obstante, hay quienes reconocen también los problemas o la posible ineficacia de estas técnicas, tanto por el reconocimiento de que las VG pueden volver a aparecer, como por el riesgo de “infección” en la zona intervenida:

GF Trav TS: *Donde… donde salió la sangre vuelve a crecer nuevamente ahí.*

GF Trav TS: *... Es una vena pues, ¿cómo no se va a infectar? [Otra:] Se ha infectado. [Otra:] Se habrá infectado esa venita.*

Desde luego, la puesta en práctica de procedimientos de este tipo no se circunscribe solo al terreno de la VG, sino que pueden ser incluidos en un rango más amplio de formas de “automedicación” y autocuidado:

ENT5 Gay: *Yo… me he echado una simple crema ¿no?, pensaba que era una herida por el calor. Desconocía del tema.*

GF Trav TS: *[Posibles hemorroides] ... ayer me dice mi amiga: “a mí me salieron unas venitas”, “ah, entonces será hemorroides” –le respondió–, “¿y qué le has hecho, no has tenido problemas? –le preguntó–, “no, agarré un Gillette y lo empecé a cortar”. [Luego le dijo:] “Pero tienes que cortarlo con guantes”. [La amiga le respondió:]”¡No, yo no lo corté!”*

**Búsqueda de tratamiento y atención médica**

En este punto podemos diferenciar dos actitudes. Están, por un lado, quienes buscaron asistencia médica desde el primer momento en que notaron la presencia de VG (aunque el resultado de esta búsqueda no siempre fue satisfactorio). Luego están también, por otro lado, quienes solo buscaron atención médica cuando reconocieron que las VG, luego de crecer o expandirse, significaron un problema serio para su salud y bienestar. En uno y otro caso, el acceso a la atención de salud aparece en ocasiones mediado por pares o “amigos” de confianza a quienes se recurre inicialmente buscando apoyo. Sobre este punto, veremos por ejemplo –más adelante– cómo en uno de los casos narrados la participación de un promotor de salud resultó clave para facilitar este acceso a la asistencia médica y el posterior tratamiento.

Con respecto a aquella primera actitud de reacción inmediata ante las VG, veamos, en primer lugar, el caso de un entrevistado (gay TS), quien acudió a un servicio de salud apenas notó que tenia VG. En su experiencia en ese servicio, percibió que no habían “especialistas”, que los médicos no manejaban bien su caso, y que éstos tenían ideas “erróneas” sobre el VPH; y menciona además que en esa ocasión no recibió tratamiento, el cual le fue prescrito recién cuando visitó un segundo establecimiento de salud, aunque –según señala– este tratamiento le resultaba muy caro y no estaba disponible en las farmacias cuando lo buscó:

ENT1 Gay TS: *(E: ¿Cómo te trató la infección el médico?) El médico sin tocarlo… o sea, no hay, no hay médico especialista en eso... que puedan… o… algunos son erróneos y dice “al hombre no le sale el papiloma.” (E: ¿Te dijeron alguna vez eso?) Sí, algunos médicos sí, dos médicos me dijeron “al hombre no le sale papiloma.” (...) En ese centro había un señor… un doctor que era… ya veterano, ya, y él tenía la cuestión esta, errónea pues “al hombre no le sale papiloma.” (...) [Luego buscó ayuda en un servicio de salud] que está en el Callao, me dio tratamiento que me echará… una crema... pero que no encontraba y era muy cara, cuando preguntaba en las farmacias no existía.*

Luego buscó la ayuda de un amigo, médico, quien reconoció que su caso era de VG y le habló de la posibilidad de “cauterizar” sus verrugas, aunque no llegó a hacerlo porque “no tenía el instrumental”:

ENT1 Gay TS: *Hablé con un pata, que era también doctor, y el sí dijo “esos son… verrugas genitales”, y quiso cauterizar. Quería ayudarme, quería cauterizarlo pero no tenía el instrumental. (E: ¿Qué es lo que sucedió?) No, no hizo nada pues.*

Esta persona señaló en un inicio que “tuvo” VG dos años atrás, pero luego de narrar su experiencia de búsqueda de tratamiento mencionó que aún tenía –al menos– una VG (“es pequeñito”). No obstante, fuera de sus estrategias para buscar ayuda médica, o de la percibida (in)eficacia de los servicios de salud en su caso, en este proceso adquirió cierto conocimiento sobre las VG, el VPH, y varios temas relacionados, incluyendo la relación entre el VPH y el VIH (“porque las verrugas se pueden rasgar”), y la posibilidad de transmitir la infección por VPH a sus parejas sexuales:

ENT1 Gay TS: *(E: ¿Alguna persona te explicó algo más acerca del virus del papiloma y las verrugas?) Sí, que tenga mucho cuidado. Porque es muy contagioso, a la otra persona le puede afectar mucho.*

En un segundo caso, al igual que el anterior, una persona (gay) buscó ayuda médica “inmediatamente” (en un “Hospital de la Solidaridad”), apenas notó que le había aparecido una pequeña VG (“como una florcita, pequeñita, diminuta… a la altura del glande”), lo cual le extrañó mucho porque era algo que nunca antes había tenido. Esta persona refiere que el médico le informó que su VG se podía tratar mediante una “cauterización” con una “pistola caliente” (aunque luego afirma que le dijeron que era “intratable”; al parecer lo entendió en el sentido de que era “incurable”). Le prescribieron también unas “pastillas” (cuyo nombre no recuerda, pero que no llegó a tomar porque “perdió la receta”). Aparentemente, el problema se solucionó con la cauterización:

ENT2 Gay: *Cuando lo vi, pues me extrañé porque nunca había visto ese cuerpo… extraño en mi… miembro, en mi pene y fui al médico para tomar información y el médico me dijo que había que... retirarla a través de una cauterización. (...) solamente lo cauterizaron y ya.*

En este caso, la persona no recibió información sobre la relación entre el VPH y el riesgo de adquirir VIH. Pero sí le informaron que se trataba de una enfermedad que “no tiene cura”, y que puede “complicar a una mujer” (pareja sexual):

ENT2 Gay: *... me dijeron que era una enfermedad que no se cura, no tiene cura, y que tampoco es tratable, que va a estar ahí siempre ¿no?, y que puede complicar a una mujer...*

Entre quienes no habían tenido VG, se mencionó también la búsqueda de atención médica para otras ITS, como en el caso de un entrevistado (ENT10 Trav TS), quien buscó ayuda profesional en una ocasión en que sentía dolor al orinar, ante lo cual el médico que lo atendió le dijo que tenía “principios” de gonorrea y le prescribió un medicamento.

Por otra parte, están aquellos que solo solicitaron apoyo profesional una vez que sus VG se habían desarrollado al punto de causarles molestias significativas, como dolor, limitaciones en las funciones del organismo, o algún impacto de consideración en la vida sexual, la autoimagen o la autoestima:

GF Trav TS: [Una amiga travesti tenía]*… una heridita... tenía eso pero yo no tenía convicción de eso, hasta que llegó un tiempo que sí, bueno, nunca lo vi ¿no? pero sí me comentaba (...) que dice que ya le dolía al momento de sentarse, al caminar rozaba con las nalgas ¿no? Lo lastimaba a ella. (...) Yo lo veía como menstruación que la sangre se bajaba y tenía que ponerse así paños para que pueda caminar ¿no? pero le molestaba ¿no? Y hasta que se enfermó una vez, se enfermó así ya, o sea, se complicó el peso y su edad que tenía; ya se fue al hospital y creo que le recetaron un medicamento que ese que lo tienen ahí, o sea lo atienden en el hospital y tenía que comprar en farmacia ¿no? Y creo que son dos… dos líquidos, algo así, que lo preparan... en las boticas, me dijeron. Entonces… [las VG] caían por partes...*

ENT9 Gay TS: *(E: ¿Te dijeron ellas [amigas trans?] cuando se dieron cuenta de que la tenían?) ... No, solamente la sintieron, lo sintieron que iba creciendo y que se fueron al hospital o clínica o por ahí, algunos centros de salud y ya, que recibieron apoyo pues.*

En casos de este tipo cobra relevancia –quizás más que en las situaciones descritas anteriormente– la presencia de mediadores que intervienen alentando o facilitando el acceso a la atención de salud. En principio, la búsqueda de estas formas de apoyo parecen estar basadas en la “confianza” que se tiene hacia ciertos miembros de esas redes de soporte social. Y por sus resultados (acceso efectivo a la atención y el tratamiento), estas redes resultarían claves en un contexto en el que el tema de las VG viene rodeado de los miedos, prejuicios y desconocimiento que conducen a la estigmatización de quienes tienen o se piensa que tienen VG. Fijémonos por ejemplo en lo ocurrido con una persona (gay), quien accedió a la atención médica para sus VG con la ayuda de un amigo, quien lo alentó a buscar asistencia profesional. Inicialmente él no quería buscar atención de salud debido a la vergüenza que sentía por tener VG. Pero luego, en un hospital, recibió información y le prescribieron un tratamiento que fue eficaz (aunque le quedaron cicatrices):

ENT11 Gay: *A mí me salieron unas verruguitas atrás y me daba un poco de vergüenza ir a verme y crecieron mucho, y luego ya cuando me molestaba mucho le conté a un amigo y me dijo para ir a un hospital y fui al hospital Loayza y ahí me dijeron que era el papiloma y me asusté mucho porque yo había leído en internet que eso te da cuando tienes el Sida y me hice mi prueba, y menos mal no salió nada y, bueno, me atendieron y me quemaron las verrugas y se cayeron; ahora ya no tengo pero era bien feo y molestoso.*

A diferencia del primer caso narrado, esta persona tuvo una buena impresión sobre la calidad del servicio de salud que recibió (“se portaron muy bien en el hospital”).

De manera similar, una travesti TS recurrió también a sus redes de apoyo. Inicialmente, esta persona no identificó su problema de salud como VG, sino como lesiones de origen desconocido, y procedió a automedicarse, sin éxito. Pero luego le comunicó su problema a una “amiga” (posiblemente una trans) que trabajaba para el “PROCETSS” (aparentemente promotor de salud), quien la llevó a un servicio de salud para que le den un tratamiento:

ENT7 Trav TS: *(E:¿Qué hiciste cuando encontraste esas verrugas?) Hacerme tratar, sí, y que las cautericen.* *(...) y yo pensé que era un raspón, un desgarro ¿no? [La trató] solo con una crema de farmacia, y luego no ha pasado nada [no funcionó la automedicación]. Entonces ahí fue que un día consulte a una amiga que trabaja en PROCETS. Y ella fue la que me llevó, pues. (...) Creo que [me] dieron un preparado para comprar, yo lo compré y él [el/la amigo/a?] me lo aplicó. Si, él me aplicó con un hisopo, tres veces creo.*

En este proceso –aunque no le hablaron de la relación entre el VPH y el riesgo de contraer VIH–, un médico le explicó que se trataba de un problema de salud adquirido por contacto sexual, el cual no tenía cura pero sí tratamiento (cauterización), y que las VG podrían reaparecer en el futuro, para lo cual debía volver para recibir tratamiento:

ENT7 Trav TS: *Me explicó el doctor que era por un contagio, sexual, y nada pues, me dijo que la tenía que cauterizar, sino se crecía, me dijo... y era que la cauterice y que… no era… no era curable, solamente se podía tratar. (E: ¿… los brotes futuros…?) Sí, me dijo “sí puede salir de acá a dos, tres años”. Sí, pero siempre, o sea: “si tienes, vuelves y yo la cauterizo”, me dijo.*

Resulta interesante apreciar cómo esta misma travesti intentó luego (aunque sin éxito) actuar como facilitadora para la búsqueda de atención médica ante un aparente caso de VG en uno de sus pares:

ENT7 Trav TS: [Su amiga parecía tener VG] *... entonces yo le comenté pues ¿no? que era ese... que tenía que hacerse ver, pero… no sé hasta ahora no me hace caso...*

**Percepciones sobre la búsqueda de atención y los servicios de salud**

Algunas personas mencionaron conocer o haber oído de personas que tenían VG y que no acudían a los servicios de salud para recibir tratamiento. Una travesti (ENT6 Trav TS), por ejemplo, habló sobre la probable presencia de VG entre sus pares, y agregó que no buscaban atención médica (aunque luego señala que no lo sabe realmente, pues ya no vive con ellas). Algo similar fue mencionado también en un grupo focal (GF Gay TS: “... yo he visto, bueno, mi amigo lo tenía, nunca fue al hospital.”)

En ciertos casos, esta actitud de no buscar tratamiento era atribuida al “descuido” de las propias personas, lo cual estaría siendo motivado por la inexperiencia o “juventud”, la falta de información, y sentimientos de “vergüenza” generados por el hecho mismo de tener VG y por las probables reacciones del entorno:

GF Gay: [Sobre el crecimiento de las VG]*... el descuido es mayormente por vergüenza. [Otra persona:] Sí, me imagino que sí, yo creo*.

ENT7 Trav TS: *Y yo le pregunté [a la amiga]: “Oye ¿qué tienes ahí?”, y me empezó a contar, ahí recién me empezó a contar y así fue. (E: ¿Qué te dijo que era...?) No sé, ni ella misma sabía porque este… chibola pues, 19 años, es chiquilla; entonces yo le comenté pues ¿no? que... que tenía que hacerse ver, pero, no sé, hasta ahora no me hace caso, porque no se ha ido a hacer ver. (E: ¿Tú crees que no se atendió por vergüenza, por miedo?) Creo que por vergüenza.*

No obstante, había también quienes juzgaban que la preocupación por la propia salud debía primar por sobre aquellos sentimientos de “vergüenza”:

ENT9 Gay TS: *Por una parte sí, [existiría] vergüenza, pero como es parte de nuestro cuerpo... y cuando uno se quiere pues, obviamente [hay que buscar atención médica], así nos cueste ¿no?*

Esta persona indica la necesidad de buscar atención médica, aún cuando la vergüenza limite esa búsqueda. Por el contexto, podemos asumir que esta persona, al decir “así nos cueste”, no se refiere al costo económico del acceso al servicio de salud, sino al *costo moral* que supondría para la persona el hecho de revelar a otros (en este caso al personal de salud) que uno tiene VG.

Sin embargo, es preciso tomar en cuenta que la “vergüenza” ante la perspectiva de acudir a los servicios de salud no se vincula única ni necesariamente con la posible presencia de VG o otras ITS, pues tal “vergüenza” bien puede estar relacionada con la idea de preservar el “pudor” frente a la idea del examen clínico del cuerpo y/o los genitales –aún en ausencia de señales visibles de infecciones:

GF Trav TS: *Es que es cierto ¿no? eh… a veces, cuando uno no conoce el tema y por el tema de vergüenza a veces no acude a un centro de salud porque; como dijeron acá, es complicado, porque las chicas van a decir: “no, es que voy a ir a medico para que me examine esa zona y todo” ¿no? [Otra persona:] Un poquito de vergüenza da.*

Cabe señalar que el panorama se presenta heterogéneo en este punto, pues así como hay quienes apelan a la idea de “vergüenza” para explicar por qué algunos no acuden a los servicios de salud, también están quienes adoptan la actitud contraria, de desinhibición frente al examen clínico:

GF Trav TS: *Pero yo digo: “si es para tu bien”, porque te revisa un hombre, te revisa una mujer, es parte de tu cuerpo; no tienes por qué tener vergüenza. Yo no, yo normal, yo me abro, me pelan, todo me hacen [Otra persona:] Ajá.*

Fuera de estos aspectos, existen unos últimos puntos relativos a los servicios de salud que debemos mencionar también. Estos puntos tienen que ver con la percepción que se tiene respecto a tales servicios. Al respecto, solo una persona indicó estar bastante satisfecha con los servicios para ITS, en los que percibe que “no hay discriminación”:

ENT9 Gay TS: *A todo que yo voy, sea acá en el centro, sea o provincia y yo justamente acá en el mismo Lima, no me quejo de las atenciones de los… de los… de los participantes donde yo voy. Siempre dan de todo y… y por lo tanto que sigan adelante, que sigan con la misma manera que no hay discriminación por nadie ¿no?*

Entre las travestis, en cambio, encontramos opiniones divergentes, referidas a la calidad de los servicios, la competencia del personal de salud, y el trato a los usuarios, en el que –según refieren– deberían primar las actitudes respetuosas hacia los pacientes:

GF Trav TS: [Parte del personal de salud] *Nos atiende un poco mejor, creo yo porque… porque ya no nos ven el comportamiento [no les juzgan], no sé, porque hay veces que nos vamos ahí, nos miran así, un poco sorprendidos*.

GF Trav TS: *Porque las que tienen solamente un buen trato en el centro de salud son las señoritas, las… ¿las que atienden los partos?... Las obstetras. Son las únicas que pueden tener mayor… porque el personal en general, no. (...) Digamos... los enfermeros, los vigilantes [no le satisfacen por su trato], en cambio las obstetras es las que nos atienden a nosotras particularmente… ellas son las que nos dan un buen trato a nosotras*.

GF Trav TS: *Hay diferentes casos también porque en clínicas especial digamos son diferentes su atención pero cuando yo me fui a IMPACTA, la que me sacó sangre, era una lindísima persona. (...) [En cambio, en otro lugar, pasaban] horas y horas ¿no? como tres, cuatro horas, no nos atendían.*

GF Trav TS: *[Debería] prevalecer la educación de la persona ¿no? o sea, más allá de todo, si una persona es educada... o sea, tiene que atender a todas las personas por igual, con el mismo trato ¿no?*

GF Trav TS: *Más que todo es la educación y el respeto que cualquier persona, creo.*

GF Trav TS: *Hablando de cosas, de algo personal, de vivencia, el doctor le trata ¿cómo sería?, como un animal al paciente. Era una de mis amigas; estaba internada, estaba mal por medio del silicon, no sé, y… estaba en emergencia, y el doctor ha tenido que tratarle como quería… le había picado un perro. No sabían dónde le picaba, le picaba por acá, le picaba por acá. (E: ¿Y tú hablaste con él?) Sí, ¿cómo vas a poner…? Si eres tú profesional... cómo no vas a saber en dónde [colocar la aguja]... no solamente a mi amiga, a varias personas*.

CAPÍTULO 4

**VACUNA VPH: CONOCIMIENTOS, ACEPTABILIDAD Y ACTITUDES**

**Conocimiento de la existencia de vacunas contra el VPH**

De entre todos los participantes en las entrevistas y los grupos focales, solo tres personas sabían de la existencia de vacunas para prevenir la infección por el VPH, y éstas eran precisamente tres de las cuatro que reportaron haber tenido VG y haber recibido tratamiento médico. Resulta entonces –como sabemos por sus narraciones– que adquirieron este conocimiento en el contexto de la atención de salud o de la búsqueda de información sobre su problema de salud (en internet, por ejemplo).

Estas tres personas manifestaron saber que la vacuna contra el VPH estaba diseñada para ser aplicada a mujeres jóvenes, antes del inicio sexual, para *prevenir* que se vayan a infectar con el VPH. Uno de ellos (gay) supo esto luego de querer saber y preguntar al personal de salud si él podía recibir la vacuna para evitar que le vuelvan a aparecer las VG:

ENT1 Gay TS: *[la vacuna]... solamente se les da a las personas menores de edad a partir… antes de los 12 años ¿no? a las mujeres...*

ENT2 Gay: *Creo que cuando… antes de iniciar su… su vida sexual, una mujer debería vacunarse contra el papiloma humano.*

ENT11 Gay: *Esa vez en el hospital me dijeron que a las mujeres las vacunaban para eso, eso sí lo sabia, pero yo incluso pregunté que si me podían vacunar para que no me dé de nuevo [las VG], y me dijeron que ya lo tenía en mi sangre y que nunca se iba a curar, que de nuevo me iba a aparecer y que tenía que ir cuando vuelva a aparecer.*

Tenemos entonces que el conocimiento sobre la existencia de la vacuna contra el VPH aparece muy asociado a la experiencia de haber tenido VG y haber buscado atención médica, salvo en un caso, el de una travesti (ENT7 Trav TS) que había tenido VG y que dijo luego no saber sobre las vacunas.

Fuera de ellos, una persona más creía haber oído sobre las vacunas contra el VPH, y pensaba que se trataba de una “inyección” que ayudaría a “controlar” el VPH:

ENT10 Trav TS: *[ha oído que] hay una… una inyección que ayuda a controlar… el virus ¿no?*

Así como en este caso, algunas otras personas asociaban también la idea de “vacuna” con la posibilidad de “controlar” el VPH, mientras que en otros casos –al ser tratado este tema durante las entrevistas o los grupos focales– surgieron ideas y preguntas muy diversas entre los participantes. En general, para muchos no queda del todo claro si las vacunas contra el VPH sirven para “prevenir”, “tratar”, “curar” o “controlar” la infección:

GF Gay TS: *(E: ¿Han oído ustedes hablar acerca de esas vacunas?) Sí (E: ¿Para qué se usan las vacunas?) Para controlar. (E: ¿Para controlar qué?) Controlar o sanar determinada enfermedad. [Otra persona:] Eso controla las defensas, ¿no? [Otra:] Refuerzan. [Otra:] Claro, son defensas de lo que va a contagiarnos, nosotros tenemos defensa para que eso lo controle. [Otra:] Ayuda a destruir, ¿no? Ayuda a nuestras defensas, algo así he escuchado. (...) [Otra] Creo que es para evitar una enfermedad pues, para controlarla. (...) (E: ¿Se les aplicaría a los que tienen papiloma o... a los que no tienen?) A los que tienen. [Otra persona:] No, a los que no tienen. [Otra:] No, a ambos. [Otra:] A los que están más expuestos. [Otra:] Tenemos el estudio de hepatitis: yo no tuve hepatitis y me vacunaron, porque si yo tenía hepatitis no me vacunaban, porque me mataba.*

GF Gay TS: *(E: ¿Se les aplicaría [la vacuna] a los que tienen papiloma o a los que no tienen?)* *A los que tienen. [Otra persona:] No, a los que no tienen. [Otra:] No, a ambos. [Otra:] A los que están más expuestos. [Otra:] El que tiene...*

**Aceptabilidad de la vacuna contra el VPH**

En general, existía entre los participantes en el estudio una actitud positiva hacia la vacuna y la posibilidad de recibirla. Solo una persona dijo en un grupo focal que no se vacunaría (GF Gay TS), debido al temor que le producen las agujas (“Yo soy de las personas que tienen pavor a las vacunas, a las agujas.”) Pero si bien los demás manifestaron que, en lo personal, aceptarían recibir la vacuna, no todos creían que sus amigos, pares, o en general la población, tuvieran la misma actitud de aceptación, principalmente por la falta de información al respecto.

Por otra parte, encontramos también algunas diferencias significativas en las respuestas y reacciones ante la pregunta sobre la aceptabilidad de la vacuna, pues diferían tanto las reacciones personales (entusiasmo, preguntas), como las ideas que sostenían las personas con respecto a para qué serviría realmente la vacuna (por ejemplo, si servía para prevenir, curar o controlar la infección por VPH). Pasamos entonces a analizar esto, junto a las motivaciones de la gente para recibir la vacuna.

Tenemos, en primer lugar, a aquellos que habían tenido o tenían VG, quienes pensaron en cierto momento que se podrían beneficiar de la vacuna para prevenir la *reaparición* de las VG, para no infectar a otras personas, para sentirse más “seguros”, e incluso para dar el “ejemplo” a otros. Vemos entonces cómo, incluso para quienes han tenido VG y han recibido información sobre el VPH, no queda del todo claro el hecho de que el VPH se queda en el cuerpo de por vida, o que la vacuna contra el VPH es solo preventiva:

ENT11 Gay:*... daría ejemplo, más bien diría “yo ya me vacuné con el papiloma” y… y me haría bien pues, estaría seguro ya contra algo más…*

ENT2 Gay: [Recibiría la vacuna] *porque quisiera protegerme. (...) (E: ¿Qué motivaría a que te vacunes?) En no contagiar a otras personas.*

ENT3 Gay: [Se vacunaría] *porque, bueno… cortaría de frente… tendría … esa enfermedad, lo cortaría ¿no? con una simple vacuna.*

ENT7 Trav TS: [Se vacunaría] *para evitar esas cosas* [las VG].

Pero luego, de entre estas cuatro personas, solo una reconoció que, aún cuando tenía una actitud positiva hacia la vacuna, personalmente no podría recibirla por tener ya la infección por VPH (aunque después manifestó sus dudas al respecto). Es decir, tenía ya por anticipado la idea de que la vacuna estaba diseñada para *prevenir*, y no para *curar* la infección. Por eso, hizo la precisión de que recibiría la vacuna *si* le informaran que está entre quienes pueden recibirla. De todas formas, le parecía positivo que exista una vacuna para prevenir el VPH, pero precisa que sería necesario determinar primero quién tiene ya la infección y quién no:

ENT11 Gay: *(E: Si estuviera la vacuna disponible en el Perú ¿tú te vacunarías?) Si entrara al rango, sí (...) O sea... si me dijeran “tú estas apto para vacunarte”, sí me vacuno. (...)* *Me parece muy bien [que exista una vacuna], pero tendrían primero saber quién lo tiene y quién no lo tiene, porque los que ya lo tienen ya no pueden ponerse la vacuna porque no les haría ningún efecto, por eso solo se les pondría a los que no la tienen para que no se contagien, como para prevenir, ¿no?* *(...) como ya lo tengo, es decir tengo ya el papiloma, ya a mi no me haría efecto ¿no? O si? No lo sé, si me ayudaría a mí, claro que me lo pongo, y de todas maneras haría que mis amigos se la pongan porque sé lo feo que es y que también es doloroso para la curación, porque te queman y te queda la cicatriz fea, a mí me ha quedado y me da un poco de palta.*

Están, por otro lado, quienes refieren no haber tenido VG. Solo entre algunos de ellos, la motivación para recibir la vacuna era la posibilidad de “prevenir” y sentirse “libres” de una infección futura:

ENT6 Trav TS: *... si me estas vacunando, es para que no te contagies ¿no? Entonces ¿de qué manera podría cambiar mi vida? Porque no me contagie: Evitar la enfermedad.*

ENT5 Gay: *[Si la vacuna estuviera disponible] Sí, claro, me vacunaría. Primero, me vacunaría ¿no? para prevenir ¿no? la enfermedad… Ya pues, después ¿no? para asegurarme también, para estar libre de eso.* *(...) si me están poniendo la ampolla, eh… creo que para una prevención; de repente más adelante la pueda contraer [la infección], pero si estoy con la ampolla, no creo.*

ENT9 Gay/Trav TS:*... es una forma más de vida para todos… ya sin tener la enfermedad vamos adelantando pues, cosa que cuando de repente llega a suceder, nos chocamos con alguna persona que ya esta contagiado, ya pues, ya nos, nos prevenimos de nosotras, ya, y no nos puede suceder con la vacuna que estamos puesta.*

ENT12 Most: *(E: Si estuviera la vacuna disponible en el Perú, ¿te vacunarías?) Sí!... Para sentirme más protegido, más seguro, para prevenir, es bueno prevenir, a veces por miedo o por dudas, uno no se quiere arriesgar y es mejor vacunarse. (E: ¿Miedo a que?) Miedo a ser contagiado, por cualquier circunstancia.*

ENT15 Most: *(E: ¿Qué piensas sobre la posibilidad de que exista alguna vacuna... para prevenir las verrugas?) Me parece muy bien, porque así ya no se infectan muchos y podemos prevenirlo pues*.

Pero fuera de estos casos, debemos destacar que si bien el objetivo de la vacunación contra el VPH sería “prevenir” la adquisición del virus, en las concepciones sobre salud de varias otras personas parece existir una asociación entre la noción de “prevención” y la idea de “estar curado” (o protegido) contra una posible infección futura. Incluso, una persona empleó la palabra “tratamiento” para referirse a la vacuna:

ENT6 Trav TS: *[Me vacunaría] porque voy a… prevenirlas, si, no… o sea… me voy a cuidar con eso ¿no?… por más que tenga relaciones creo que voy a estar curado contra [el VPH].*

ENT13 Most: *[Recibiría la vacuna] Para curarme o para no contagiarme de esas verrugas.*

GF Trav TS: *Es mejor esa ampolla, como una cura*.

ENT10 Trav TS: ... *así de tan fácil, no creo que… sigan ese tratamiento porque no ha habido una previa información.*

Para un joven gay, la vacuna podría ayudarle a controlar alguna posible infección que posiblemente ya tiene (actualmente) pero que no se manifiesta aún (“porque uno no sabe de qué está mal internamente”) [recuérdese la clasificación de ITS: las que ocurren “por dentro”, y las que se manifiestan “por fuera.”] Este joven agregó también que se animaría a recibir la vacuna si ésta fuera gratuita:

ENT4 Gay: *La verdad que no, no, no sabía [de la existencia de las vacunas contra el VPH], pero bueno que si, si se ponen en marcha estos tipos de vacunas y son gratuitas, sí, sí me la haría ahora.*

Como en el caso de aquellos que habían tenido VG, también otros mencionaron que la vacuna podía ayudar a proteger a las parejas sexuales:

ENT13 Most: *(¿Cual seria esta razón [para vacunarse]?) Es que yo tengo mi enamorada y ella no sabe todo sobre mí, y no me gustaría malograrla pes.*

Resultó interesante comprobar que, para varias personas, la posibilidad de recibir la vacuna era apreciado como una oportunidad para recibir información sobre el VPH y las VG. Un entrevistado (gay), por ejemplo, refirió que recibir la vacuna le ayudaría a saber más sobre “enfermedades nuevas que salen”.

[Comentario: La posibilidad de “prevenir” una infección es un beneficio *hipotético*, que apunta a la posibilidad de evitar un evento futuro que podría ocurrir o no. Frente a esto, el recibir información sería un beneficio real y palpable.]

Cabe mencionar que, en el contexto de las entrevistas y los grupos focales, la mayoría de los participantes en el estudio tuvo la oportunidad de observar fotografías de casos reales de VG. Hemos señalado previamente (en el primer capítulo, sobre “Conocimientos...”) que estas imágenes causaron un gran impacto entre quienes las observaron, y sobre todo entre aquellos que no habían tenido ni observado nunca tales VG. Por eso, es preciso tomar en cuenta que la voluntad de estas personas para recibir la vacuna puede estar influenciada, en mayor o menor medida, por la visualización de esas fotografías, como se puede apreciar en estas citas:

ENT8 Gay/Trav: *(E: ¿Tú sabías que existe una vacuna para prevenir la infección del virus del papiloma humano?) Me quiero poner de hecho esa vacuna, porque la verdad... de verdad me quiero poner con eso, porque me siento incómodo [por ver las fotografías de VG]*

ENT13 Most: *(E: ¿Que te motivaría a ti a vacunarte?)* *¡Ja ja ja ja!… con esas fotos que me has enseñado, cómo no voy a querer vacunarme, ¡ja ja ja!, yo no quiero estar así.*

ENT15 Most: *(E: Si estuviera la vacuna disponible en el Perú, te vacunarías?) Si!... para prevenir pues, porque uno no quiere tener eso en el pene ni en ninguna parte del cuerpo [refiriéndose a las imágenes de VG]*.

En ocasiones, el ánimo por querer recibir la vacuna estuvo asociado con una auto-percepción de ser “promiscuo”. En esos casos vemos cómo la auto-percepción del propio riesgo parece estar asociada con una mayor voluntad de querer recibir la vacuna:

ENT8 Gay/Trav [el más entusiasta ante la perspectiva de recibir la vacuna]:*... la verdad yo soy bien franco doctor... yo soy promiscuo, a mí me gusta el sexo, de nantes’ acabo de tener sexo con un muchacho.*

Otro punto a tomar en cuenta es que las iniciativas para introducir vacunas contra el VPH, dirigidas a hombres, se desarrollarían en un medio en el que existen ya antecedentes de intervenciones de prevención y control del VIH y otras ITS, lo cual estaría ofreciendo un contexto favorable para las nuevas vacunas contra el VPH. En las siguientes citas, por ejemplo, vemos que la posible vacuna es vista como una protección adicional (“una forma más de cuidarme”), colocada en el marco de nociones previas sobre prevención del VIH y otras ITS:

ENT9 Gay/Trav TS:*... en primer lugar, claro [se vacunaría] ¿Por qué? Porque es una forma más de cuidarme, de protegerme eh… cómo prevenir las enfermedades, y es igual que… como el VIH, pues, ¿no?*

ENT9 Gay/Trav TS: *Es una enfermedad menos*.

GF Gay: *Todo es VIH, VIH, ya bueno, ya, te haces la prueba del VIH, te cuidas, pero no acaba ahí pues, hay otras cosas y... qué bueno que hay una campaña, porque de todas las ITS en las que te puedes pegar por lo menos ya sabes que puedes encontrar la vacuna… una vez.*

Cabe señalar que algunas personas expresaron ciertas dudas y reparos relacionados con los posibles efectos adversos de la vacuna, o por alguna “contraindicación” (ENT7 Trav TS). Estos reparos podían estar precedidos por alguna experiencia con otras vacunas recibidas anteriormente. No obstante, tales preocupaciones no se contradecían con la actitud de aceptación hacia la vacuna, que – como mostramos en las líneas previas– fue casi generalizada:

ENT10 Trav TS: *[Se vacunaría] Siempre y cuando no tenga reacciones anormales o una reacción que me vaya a afectar, no tendría ningún problema.*

ENT14 Most: *[Se vacunaría] Para prevenir, porque uno nunca sabe... (E: ¿Habría algo que te haría dudar si vacunarte o no?) Saber cuáles son los síntomas, porque sé que algunas vacunas traen síntomas y mucha fiebre, ¿no? (...) Es que yo me vacuné de hepatitis B en mi trabajo y me dio dolor de cabeza como tres días*.

GF Gay TS: *Mientras no tengan efectos secundarios, encantado de la vida [recibiría la vacuna]. [Otra persona:] Claro.*

GF Gay TS: *¿La vacuna sería una sola de golpe o… mensual, o no sé, al año?,* *o sea, ¿qué dosis es?* *[Otra persona:] ¿Cuántas dosis?*

Como mencionábamos al inicio de este apartado, la percepción sobre la posible aceptación de la vacuna por los amigos, pares o la población era percibida de formas distintas. Algunos pensaban que tales vacunas serían bien recibidas por otros, sobre todo porque servirían para proteger la salud:

ENT3 Gay: *Yo creo que sí [se vacunarían sus amigos] porque si es para su salud, mucho mejor.*

ENT6 Trav TS: *Creo que sí [se vacunarían] porque… a nadie le gustaría tener eso y contagiarse de eso...*

ENT7 Trav TS: [Sus amigas travestis aceptarían vacunarse porque] *a nadie le gustaría tener verrugas en el ano*.

ENT9 Gay/Trav TS: *(E: ¿crees que tus amigos se vacunarían?) Creo que sí, si es para el bien de uno, es para el bien de nuestra salud, yo supongo que sí.*

ENT15 Most: *(¿Qué motivaría a que tus amigos se vacunen...?) Saber que es para prevenir y que no se contagiarán.*

Una persona que había tenido VG dijo que animaría a sus amigos para que se vacunen, para que no pasen lo mismo que había pasado él:

ENT11 Gay: *... haría que mis amigos se la pongan porque sé lo feo que es, y que también es doloroso para la curación, porque te queman y te queda la cicatriz fea, a mi me ha quedado y me da un poco de palta.*

Pero había también quienes pensaban que la vacuna no necesariamente sería aceptada por las demás personas, sobre todo debido a la escasez de información al respecto:

ENT2 Gay: *(E: ¿Crees tú que en Perú los gay están preparados para empezar a usar una vacuna como esta?) ¿Preparados? Creo que no. No, no estamos; digo “estamos” porque tampoco estaba enterado de esto, no estamos enterados o informados.*

ENT3 Gay: ... *como todavía no sale la vacuna... dirían… dirían “oe ¿qué es esto?” lo verían algo extraño.*

ENT5 Gay: *[Los “gay” no están preparados para recibir la vacuna] Porque, en primer lugar, algunos este…desconocen el tema, y algunos a veces por intención propia no, no se quieren hacer, o sea desconocen y lo ignoran a un lado.*

ENT10 Trav TS: *[Sus pares] no han tenido la orientación necesaria, la información... sobre este virus. Por eso te digo que así de tan fácil, no creo que… sigan ese tratamiento porque no ha habido una previa información.*

GF Gay: *(E: ¿Qué otras barreras creen ustedes que existirían para que las personas decidieran vacunarse o decidieran no vacunarse contra este virus?) Poca información creo. [Otra persona:] No hay mucha información sobre el VPH. [Otra:] No hay mucha información. [Otra:] Creo que debería haber más información.*

En un grupo focal se mencionó incluso que habría gente que, asumiendo que la vacuna sirve para curar o controlar el virus, no querría ser vacunada debido a que considerarían que la vacuna debiera ser para quienes tienen la infección:

GF Gay TS: *(E: ¿Por qué creen ustedes que la gente no se vacunaría?) A veces por miedo. [Otra persona:] También es por la misma sociedad que es muy compleja también. (E: ¿En qué sentido?) En que… algunos van a decir que no, “yo no lo tengo [la infección], que se vacunen [los] que la tienen.”*

Algunas personas asumían que en sus grupos, en general, habría un cierto “desinterés” por temas de salud como la prevención de ITS:

ENT2 Gay: *Creo que algunos [hombres], sí, o algunos no le… no tomarían tanta… tanto interés. Quizá por su modos de ser ¿no? (E: ¿Cómo sería su modo de ser?) Desinteresados y no prestan atención a las cosas...*

ENT8 Gay/Trav: *... una que otra vienen... disculpando la palabra, vienen con el “Bu” ya de allá... y el “Bu” acá en Perú, nosotros las lo... los mariconcitos le llamamos el Sida, tú sabes... Y la verdad vienen con el “Bu”, y le digo: “Oye, loca, ¿te lo comiste?”... [La responden] “y con todo, maricón, y con cuatro…” (...) O sea, hay mucha gente doctor que no previene... las enfermedades, la verdad.*

Entre los jóvenes llamados *mostaceros* se observó una actitud común que difería notablemente de las de los participantes de otros grupos, en el sentido de que mostraron un mayor escepticismo respecto a si sus pares aceptarían vacunarse. Es así que veían difícil que sus amigos vayan a querer recibir una posible vacunación, y parecía importarles mucho el que se tome en cuenta la autonomía y la individualidad “de cada quién”. La perspectiva casi general entre ellos fue que sus pares le estarían restando importancia a la posibilidad de adquirir ITS, o que preferirían mantener en reserva los asuntos relacionados con el sexo con hombres gay o travestis:

ENT13 Most: *(E: Tú crees que tus amigos, los patas con los que andas y que sabes que también salen con chicas y gays o travestis, ¿se vacunarían?) Eso sí no sé, la mayoría no es de hablar de esas cosas, no creo que les importe mucho. (...) Pero la verdad no sé, eso es de cada quién, a algunos no les importa nada.*

ENT12 Most: *(E: ¿Tú crees que en el Perú los chicos como tú están preparados para recibir una vacuna como esta?) No creo, porque aún falta mucha educación, algunos no le dan importancia a esas cosas.* (...) *(E: ¿Crees que por ejemplo tus amigos, tu entorno con los que paras, se vacunarían?) La verdad, no sé, porque cada uno como que es un tema personal que tienen cada uno, otros se sienten más seguros [previamente], tienen su ego, que “!no, no!”, que “yo para qué necesito”, que sería problema de cada uno.*

ENT14 Most: *(E: ¿Crees tú que tus patas de aquí del barrio con los que paras, y que suelen tener también sexo con algunos chicos “gays”, se vacunarían?) Ahhh, no sé, ¡eso es de cada quien! Aquí todos son caletas [reservados], siempre niegan que se meten con los patas, ¿o no? Aquí siempre te dicen que “no pasó nada”, que solo “relojean”, pero que no hacen nada. (E: ¿A qué te refieres con que “relojean”?) Que dicen que solo se vacilan tomando y juergueando, pero que nunca “tiran” pes, ¡pero no es verdad!*

ENT15 Most: *(E: ¿Crees que en el Perú los chicos como tú están preparados para recibir una vacuna como esta?) No lo sé, a veces creo que sí, pero a veces creo que no porque mis patas no quieren ni hacerse la prueba.*

No obstante, estos jóvenes consideraron también que sus amigos podrían aceptar ser vacunados si se llega a ellos con explicaciones sobre los beneficios que supone la vacunación:

ENT14 Most: *(E: ¿... cómo crees que podríamos convencerlos para que decidan vacunarse contra este virus?) Buena pregunta, fácil diciéndoles que es para su bien y que es para que se prevengan. O decirles que para que cuando saquen su carnet de la municipalidad no les digan nada. Si chocas con el trabajo ahí sí se vacunan contra todo, ja ja ja ja. (E: ¿Crees que en el Perú los chicos como tú están preparados para recibir una vacuna como esta?) Yo creo que sí, la gente ya es mas consciente, aunque a veces hay gente que no entiende con nada.*

ENT12 Most: *(E: ¿Qué haría que tus amigos se vacunen o se sientan cómodos para irse a vacunar?) Decirles qué les puede pasar, y tratarlos bien, porque algunos son muy brutos, ¡je je je!*

ENT15 Most: *(E: ¿Crees que tus amigos, los patas con los que paras en esas fiestas donde conocen gays, se vacunarían?) Sí, supongo que si se les explica como me estás explicando sí lo harían, porque a veces solo falta que te expliquen.*

Varias otras personas ofrecieron también alternativas para abordar el tema de la falta de información. Al respecto, dijeron por ejemplo que se podría “convencer” a la gente brindando información sobre las “causas” (de las VG), el riesgo que representan, y lo que pueden hacer al respecto:

ENT3 Gay: *Los convencería, bueno diciéndole ¿cuáles son las causas?, ¿Cómo podría eso… infectarse más?, ¿Cuál es… cuál es el procedimiento que podría seguir después?, ¿no? Yo creo que haciéndole entender, sí, sí aceptarían.*

Con respecto a las formas de transmitir la información, muchos expresaron su preferencia por las formas interactivas (cara a cara), como “charlas” o reuniones informativas:

ENT2 Gay: *(E: ¿Qué harías tú para que tus amigos se vacunen?) Los invitaría a mi casa, ahí a una reunión y les explicaría acerca del tema, principalmente sobre los riegos. Pa’ que estén… pa’ que tomen en… en cuenta que es peligroso, y si está la vacuna, esa sería la solución.*

ENT4 Gay: *(E: ¿Crees que se vacunarían tus amigos gay?) Si les hablo así como tú me estás hablando, también sí, se vacunarían... porque… es que uno no sabe qué tiene internamente.*

Otra forma de animar a las personas a recibir la vacuna sería mostrarles primero el mismo tipo de imágenes de VG que los participantes observaron, apelando al “miedo”, o a la idea de “riesgo”, para que vean qué es lo que podría ocurrirles si contrajeran la infección (“para que se traumen un poco”):

ENT5 Gay: *(E: ¿Qué los motivaría a que sí acepten vacunarse?) Quizá mostrándoles algunas fotos o videos que en otras personas se dio eso y comprueben; se animarían también.* (...) *La mejor forma seria ¿no? que ellos vendrían un día acá, o de repente nosotros salir, hacer como una propaganda y hacerlo [ver] qué es lo que pasa con unas imágenes, todos así; y la gente ve lo que tiene, lo que no tiene, ya pues ahí la gente se va a dar cuenta y se anima.*

ENT6 Trav TS: *[Para que se animen a vacunarse convendría]... que den una clase de eso que… como… que como tú me has explicado con imágenes y todos eso; con dar una clase y ya van a entender lo que puede pasar si no se vacunan.*

ENT8 Gay/Trav: *(E: Y según tu experiencia, ¿cómo harías tú para que ellas se animen a vacunarse?) La verdad que le contaría lo que he visto en foto y pediría a usted al correo, la página de lo que estoy viendo para tomarle foto a eso y bajar esas fotos y mostrárselo y decirle: “mira maricón lo que he visto en la pagina y esto es lo que puede pasar... sabrás maricón que esto…”, le enseñaría las fotos para que se traumen un poco y les animaría a decirle, ¿Cuándo le están dando de propina para que vengan conmigo? Y se ayudarían.*

ENT11 Gay: *Sí, de hecho [sus amigos se vacunarían]. A todos nos da temor contagiarnos y si nos explican bien pues sí se vacunarían, además a mis mejores amigos les contaría lo que me ha pasado para que se vacunen, no quiero que ellos pasen por lo mismo (...) [para animarlos a que se vacunen] Les contaría que si no se vacunan les saldría y tendrían que volverse activas, jajajajaja. No, mentira, les diría que vean las fotos, porque yo ya no tengo [VG en el cuerpo] para mostrarles, jajajajaja. Con charlas también, y con explicaciones como las que me has hecho, eso les gustaría y los animaría.*

ENT13 Most: *(E: Según tu punto de vista, ¿que crees que haría que ellos [sus amigos] quieran vacunarse?) Ja ja ja ja!, si les muestras las mismas fotos fácil que se vienen a vacunar.*

Afloró también el cuestionamiento sobre si la vacuna sería gratuita o no:

ENT1 Gay TS: *(E: ¿Crees tú que tus amigos, tu entorno de amigos, se vacunarían?) Sí, se vacunarían si fuera… numero uno si fuera gratis pues, y numero dos si, si les dieran una… breve charla, así como la que estoy tomando ¿no?*

ENT14 Most: *(E: ¿Cómo invitarías a chicos como tú para que se vacunen?) No sé, por folletos, charlas, fotos, y decirles que es gratis, ¿sería gratis? (E: Es lo mas probable.) Entonces sí, ni hablar, todos vendrían, todos se vacunarían. (E: Porque crees que al ser gratis se vacunarían?) Ja ja ja ja!, es que todo lo gratis es bienvenido, y si es para prevenir mejor pes.*

**Percepción de posibles reacciones del entorno social ante la vacunación**

La mayoría de los participantes señaló que, de recibir la vacuna, lo comentarían con sus familiares y, en menor medida, con sus amigos. Un joven (ENT2 Gay), por ejemplo, dijo que lo contaría a sus familiares por el “aprecio” que les tiene, mientras que muchos pensaban que sus familias los respaldarían en la decisión de recibir la vacuna, principalmente porque se trababa de un tema de salud. En general, los familiares, más que los amigos, son percibidos como una fuente de apoyo en cuestiones relacionadas con la salud, incluyendo el soporte ante posibles contingencias:

ENT3 Gay: *[Su familia no le cuestionaría si decidiera recibir la vacuna] porque lo estoy haciendo por mi salud.*

ENT5 Gay: *[A sus familiares les contaría sobre su vacunación] Para que... les comentaría ¿no? porque estén al tanto también de mí, por A o B sucediera algo, ya pues ellos están pendientes.*

ENT14 Most: *(E: Dime, ¿le contarías a tu familia o tus amigos que te vacunarías?) Sí, para que estén enterados. (E: ¿Por qué se los contarías?) Bueno, a mi familia porque uno no sabe qué te puede pasar con las vacunas, hay tantas cosas, y para que estén al tanto por si me pasa algo.*

ENT6 Trav TS: *(E:¿Crees que tu familia o tus amigos te dirían algo por que te vacunaras? O ¿se molestarían quizás...?) No, porque si… les explico que… que vean, o sea, me voy a vacunar para... prevenir esa enfermedad, creo que es más, estarían de acuerdo conmigo.*

ENT9 Gay/Trav TS: *[Nadie la cuestionaría si se vacunara] Porque en primer lugar, vivo sola, soy independiente, yo sola tomo decisiones sin consultar con nadie. Y si mis amigas me llegarían a preguntar, no me hiciera problemas, al contrario, ellos… ellos me apoyan en todo lo que yo pueda estar.*

ENT11 Gay: *(E: ¿Crees que te preguntarían, por qué te quieres vacunar?) Sí, pero si no les gusta igual me vacunaría porque es para mi bien.*

En uno de los casos, el de mayor entusiasmo ante la posibilidad de recibir la vacuna, el percibido apoyo familiar se enmarcaba en experiencias previas de haber participado en estudios o intervenciones de salud, y de haber sido respaldado por la familia:

ENT8 Gay/Trav:*... la verdad es que mi madre se siente contenta y feliz de que yo pare con las hojas, con que IMPACTA o que Procets... y me dice: “¿por qué te gusta salir, ah? (...) cuídate hijo, porque... la verdad no te voy a cuidar yo, ...yo te cuido de la puerta pa’ dentro, pero no te cuido de la puerta pa’ fuera”, me dice mi madre...(...) (E: ¿Crees que tu familia o tus amigos se incomodarían o se molestarían si empezaras a usar eh… la vacuna contra el papiloma?) Mire, ni mis amigos, bueno podría ser que mi familia me incomode porque yo vivo en casa de mi mamá, bueno aunque mi mamá (...) ahora a mi mamá le enseñe el consentimiento que tengo, “¡mamá, mira!”, dime: “Ay… tú y tus cosas, pero dame los resultados”; “¡ay mamá! mira mamá”, “¡ay, hijito ven!”, me abraza y besa, ese día lloré con ella porque me siento contento doctor, porque hasta el momento estoy libre de todo mal, pero no me siento seguro de nada porque mañana más tarde, doctor, qué nos puede pasar. (...) (E: Entonces ¿tu familia te apoyaría?) No, no. Mi mamá, mi papá y mis hermanos están de acuerdo, “bueno pues”, ya pueden decir, “te volviste gay, gay eres y terminaras siendo gay”, pero sí me apoyan doctor, porque inclusive mi hermana me llamó anoche... y cuando suena mensajero pues… [le dicen:] (...) “Oye, no te vayas a olvidar mañana tienes cita en IMPACTA, nueve de la mañana, te levantaré a esa hora…” (...) Mis hermanas y mis padres me apoyan bastante doctor, a que yo me cuide.*

Con respecto a los amigos, algunos incluso dijeron que lo comentarían con ellos con la perspectiva de alentarlos a que ellos también reciban la vacuna, que es a veces percibida como un beneficio, o un “apoyo” en sí mismo. Y además, como en un caso narrado previamente, la voluntad para comunicar a otros que se ha recibido la vacuna estaría teniendo relación también con experiencias de haber participado anteriormente en otras iniciativas de prevención de ITS:

ENT9 Gay/Trav TS: *... yo ya tengo una experiencia, voy a tener una experiencia en este caso ¿no?... que yo estoy haciéndome mis controles, ya por tanto voy a prevenir, tanto mis amigas, como mis amigos y personas que… que conozco, por conocer para dar un alcance más ¿no? un alcance más o apoyo más que brinda la empresa también.*

En general, la mayoría diferenciaba la probable actitud de la familia (que tiende a ser positiva o de apoyo) de la reacción prevista de sus amigos o conocidos (que podría ser de escepticismo). Por ejemplo, una travesti (TS), quien dijo que le hablaría a su familia sobre su posible vacunación (esperando una reacción favorable “porque es ver por mi salud”), señaló también que otras personas de su entorno social (conocidos o pares) la verían con sospecha, e incluso la cuestionarían apelando a un discurso fatalista, como aquel según el cual “de algo hay que morirse”:

ENT10 Trav TS: *[Amigos] me dirían: “pues, oye qué tanto haces controlándote si…” La típica respuesta que siempre dicen, que “de algo hay que morirse” ¿no? entonces, yo creo que como que la familia no te… te contestaría así o no te aconsejaría de esa manera.* *(...) creen [sus amigos] que como… pues ¿no? que de todas maneras, en este mundo, pues todos vamos a partir, pero hay que prevenirse porque hay formas ¿no? hay forma y si uno se puede cuidar...*

Unas cuantas personas consideraban que sus allegados o conocidos les respaldarían luego de una reacción inicial de duda o preocupación, ante lo cual tendrían que proceder a ofrecerles una explicación sobre la vacuna y el objetivo de la vacunación:

GF Gay: *Mi familia, digamos, mi Mamá es bastante conservadora, aunque está siempre abierta a escuchar nuevas cosas; estoy seguro que si voy y le digo que me he vacunado contra… lo que me va a decir y “¿por qué?”, “ah, porque es una nueva forma, es una forma de prevenir contra tal…”, empiezo a explicarle un poquito de lo que es el virus y todo eso. “¡Ay, este muchacho!,” me dice ¡pu!... Yo creo, o sea, no habría tanto estigma con la familia porque al final no es una infección exclusiva [de mujeres] ¿no?*

De manera similar, un joven gay (TS) manifestó que si decidiera vacunarse lo comentaría primero con su familia, y luego, “si es necesario”, también con sus amigos, aunque piensa que, en un principio, algunos podrían cuestionarlo o verlo “con malos ojos”, lo cual atribuye a una percibida falta de “conciencia” en su entorno, sobre todo entre los “gay”:

ENT1 Gay TS: *Al principio, sí [lo cuestionarían]... porque lo verían a uno pues con malos ojos ¿no? miran así mismo pues que también son personas este… no conscientes. (...) [los hombres gay] como que se cierran en un mundo en que quieren ser ellos solos, que son perfectos, que son gay, que nunca salen a la calle, no son promiscuos. En realidad, su misma situación es la que se hace todo esto.*

El hecho de que se tome en cuenta o no las probables reacciones negativas del entorno social con respecto a la vacuna estaría asociado, en buena medida, a la percepción sobre la propia independencia y capacidad para tomar decisiones autónomas. Es lo que sugiere una comparación entre las ideas vertidas en las entrevistas con las opiniones brindadas en los grupos focales. En éstos últimos surgió la idea socialmente compartida de que el hecho de recibir la vacunación debía ser comunicado a los familiares, algo más que a los amigos. En ciertas entrevistas, en cambio, algunas personas, principalmente las que se dedicaban al comercio sexual, manifestaron que si bien tomaban en cuenta a su entorno social, procederían según su propio criterio (ENT7 Trav TS: “soy mayor de edad”), ya sea por que no vivían con sus familias, o porque se percibían como personas autónomas en sus decisiones sobre la sexualidad y la salud:

GF Trav TS: *(E: ¿Tendrían problemas con sus familiares si decidieran usar la vacuna...?) A la mayoría de acá, digamos no… [Otra persona:] No vive con familia. [Otra:] Son independientes. [Otra:] Son independientes.*

ENT2 Gay: *(E: ¿Crees que tu familia o tus amigos te cuestionarían o se molestarían si decidieras vacunarte?) No, porque yo… soy una persona totalmente independiente y tomo mis decisiones propias. Si se las comento quizás se incomodarían, pero ya es una cuestión mía de comentárselos o no.*

ENT4 Gay: *[Si decidiera recibir la vacuna, el asunto para él]* *es personal, es mi intimidad (...) [los familiares] no se tienen por qué enterar.*

La idea de no comunicar a otros que se había recibido la vacuna estuvo asociada a una percibida posibilidad de sufrir estigmatización, por la aparente vinculación que otros podrían establecer entre el hecho de recibir la vacuna y la idea de que uno tiene VG u otras ITS. Es el caso de un joven gay, quien al parecer no tenía claro aún que la vacuna debía ser aplicada a personas que no tenían el VPH, y que manifestó que de recibir la vacuna preferiría no comentarlo con sus amigos o familiares por “vergüenza” y para evitar que las personas de su entorno se alejen de él:

ENT3 Gay: *[No comentaría a otros que recibió la vacuna] Por un poco de… de vergüenza. Porque al infectarme yo no quisiera que ellos estén alejados de mí o… o me rechacen por cualquier… por cualquier… puede ser cubiertos o cosas, pensando lo peor.*

De manera similar, otros también aparecen en el escenario hipotético de haber recibido la vacuna manejando selectiva y estratégicamente la información relativa a la vacunación, incluyendo los documentos con resultados de pruebas diagnósticas, para evitar la estigmatización asociada a las ITS. Un entrevistado, por ejemplo, señaló que si él se vacunara y su familia lo supiera, ellos inicialmente podrían asociar la vacunación con la presencia de alguna infección en él. No obstante, él podría también informarles que se trata de un asunto de prevención, y que en ese caso no le cuestionarían. Y por otro lado, con sus amigos, adoptaría una estrategia algo distinta para evitar posibles “burlas” o actitudes de escepticismo:

ENT5 Gay: *(E: ¿Qué crees tú que diría tu familia si es que saben que te has vacunado...?) Creo que pensaría ¿no? si de repente yo lo tengo, o de repente si estoy afectado, pero sí yo le diría... que es una prevención; vayan a pensar un poco ¿no? pero después ya creo ya, si es para mi bien, no… no creo que me digan nada.* *(...) A mis amigos no les comentaría hasta que salgan los resultados. (E: ¿Por qué no les contarías...?) No… porque de repente lo pueden tomar a mal, de repente [se] pueden burlar o de repente [si] llego a decir la verdad, ya no me creerían, pensarían que estoy infectado ¿no?*

Tres de los cuatro jóvenes *mostaceros* entrevistados percibían que serían apoyados por sus familias si decidieran vacunarse:

ENT12 Most: *(E: ¿Tú le contarías a tu familia o tus amigos que te vacunarías?) Sí, si hay el momento sí se lo contaría, para que sepan, estén enterados. (E: ¿Crees que tu familia o tus amigos te dirían algo o se molestarían...?) No, al contrario, me ayudarían como cualquier familia. (E: ¿No crees que te preguntarían, por qué te quieres vacunar?) Eso sí, sí me preguntarían, pero les diría que es una forma de prevenir y estar seguros consigo mismo, y nunca está de más prevenir.*

ENT14 Most: *(E: Dime, ¿le contarías a tu familia o tus amigos que te vacunarías?) Sí, para que estén enterados. (E: ¿Por qué se los contarías?) Bueno, a mi familia porque uno no sabe qué te puede pasar con las vacunas, hay tantas cosas, y para que estén al tanto por si me pasa algo. (E: ¿Crees que tu familia o tus amigos te dirían algo o se molestarían...?) No, al contrario, porque saben que es para mi bien.*

ENT15 Most: *(E: ¿Tú le contarías a tu familia o tus amigos que te vacunarías?)* *Sí, ¿por qué no? ¿acaso es algo malo? (E: No, claro que no, pero, ¿tu familia o tus amigos te dirían algo o se molestarían...?) No, no creo, ellos me apoyarían. (E: ¿No crees que te preguntarían, por qué te quieres vacunar?) Sí, pero les diría que es para mi bien, que nadie esta libre de nada.*

Pero el cuarto, en cambio, tuvo una opinión contrastante, pues percibía que la decisión de vacunarse o no sería en su caso algo muy personal, que mantendría en privado, por su expectativa de que si lo revelara a otros podría ser blanco de burlas relativas a su “debilidad” (refiriéndose al sexo con hombres):

ENT13 Most: *(E: ¿... le contarías a tu familia o a tus amigos que te vacunarías?) No, eso es algo mío, ellos no tienen por qué saberlo. (E: ¿Por qué no deberían saberlo?) Porque son cosas mías, y después se burlan porque se enteran que uno tiene sus debilidades pes. (E: ¿A que debilidades te refieres?) Ja ja ja ja... que a uno le gusta la “mostaza” pes, y luego se entera todo el barrio. (E: ¿Crees que tu familia o tus amigos te dirían algo o se molestarían...?) Sí pes, se burlarían, yo conozco a mis hermanos y a mis patas, son jodidos.*

**Posible impacto de la vacuna en la vida y las prácticas sexuales**

En general, la mayoría de los participantes no percibía que recibir la vacuna contra el VPH vaya a significar un cambio en sus prácticas sexuales, en el sentido de una posible “desinhibición” sexual. Por el contrario, varias personas consideraron que la vacunación contra el VPH podría tener un impacto positivo en sus vidas, en el sentido de que recibirían y manejarían información sobre el tema (un tema ampliamente desconocido, como se ha mostrado previamente), información que les ayudaría a “protegerse” más para prevenir posibles infecciones, o les llevaría a pensar más en los posibles riesgos a los que se exponen al momento de tener relaciones sexuales:

ENT1 Gay TS: *(E: ¿Crees tú que la conducta sexual de la gente cambiaría si se vacunan contra el virus del papiloma humano?) Cambiaría en algunas personas, ¿no? y cambiaria un poco pues... porque en cada... charla motivacional que da... estos centros como IMPACTA, esto motiva a que una persona se cuide más.*

ENT4 Gay: *[Quienes reciban la vacuna] Se protegerían más porque saben que ya existen estos tipos de virus.*

ENT3 Gay:*... va a ser un cambio, pues ¿no?, o sea con vacuna ya creo que uno… va a pensar con quién se mete, creo.*

ENT12 Most: *(E: ¿Crees que tu conducta sexual cambiaría si te vacunaras? porque a veces la gente dice, “bueno ya me vacune ahora no uso condón”) No, al contario, ya es como una advertencia, ahora te estamos vacunando, con mayores charlas, al contrario, ya como que tomaría conciencia.*

[Comentario e interpretación:] Por lo manifestado por estas personas, conviene que nos detengamos un momento en este punto, pues guardaría relación con algo que habíamos notado ya previamente, cuando señalábamos que la información recibida en el contexto de la vacunación podría estar siendo percibida, en sí misma, como un beneficio palpable, mientras que la “protección” ofrecida por la vacuna sería más bien un *beneficio hipotético* referido a un probable evento futuro que se busca evitar (la posible infección por el VPH o la aparición de VG). Esto puede ser ilustrado de mejor manera con el caso siguiente, el de una travesti trabajadora sexual, quien consideraba también que recibir la vacuna podría afectar la conducta sexual de la gente en un sentido positivo, ya que –según ella– las personas tendrían más en mente la existencia de la infección luego de recibir información al respecto (mediante “clases”, con “imágenes”). Esta información ayudaría incluso a que quienes aceptan tener sexo sin condón por dinero se detengan a pensar en el riesgo de infección antes de aceptar ese tipo de propuestas:

ENT6 Trav TS: *(E: ¿Crees que tu conducta sexual... [o la] de la gente en general cambiaría si se vacunara?) Yo creo que sí porque ya tendrían más cuidado... ya se pondrían a pensar. Pero hay algunas que por la plata le dicen: “sin preservativo te voy a dar más, más, más”, y la plata es bien tentadora, y creo que cuando… si como tú dices, si le das unas clases de eso, con imágenes y todo eso, o sea, van a poder pensar antes de hacerlo, por más que le aumenten dinero.*

Si bien se supone que la vacuna ofrecería protección contra la posibilidad de adquirir el VPH al tener relaciones sexuales, esta persona consideraba que recibir la vacuna haría también que sus pares reflexionen antes de aceptar propuestas de sexo sin condón a cambio de más dinero. Es decir, piensa que se protegerían más, empleando condones. Esto, en principio, parece contradecir la idea de que la vacuna propiciaría una “desinhibición” sexual, pues en el caso de esta travesti, como también en las citas previas, la vacuna parece estar teniendo en la conducta sexual un efecto contrario al de la “desinhibición”. No obstante, al parecer, este efecto contrario (de mayor cuidado) no se debería precisamente a la vacunación, ya que si tomamos en cuenta que para estas personas el hecho de recibir la vacuna significaría también *recibir información* sobre el VPH y sus posibles consecuencias, resulta entonces que la percibida motivación para tener sexo más seguro estaría no en el hecho aislado de recibir la vacuna, y menos aún en la idea de estar protegido contra el VPH (el beneficio hipotético), sino que tal motivación para el sexo seguro tendría su origen en la información que acompaña a la vacunación. Y si ampliamos un poco el panorama veremos que la *expectativa* de recibir información en el *contexto* de la vacunación (el beneficio real: recibir consejería, “charlas”, etc.) sería un factor importante, no solo como posible motivador de prácticas sexuales más seguras, sino también como un elemento que las personas estarían tomando en cuenta al momento de decidir recibir la vacuna (como sugieren los datos presentados en otras secciones de este informe). Veamos por ejemplo este comentario surgido en un grupo focal:

GF Gay: *... cuando yo me hice el examen de VIH, o sea, tenía en la cabeza toda la información fresca, y dije “bueno, yo quiero seguir siendo negativo”, y no sé, yo salí más concientizado de hacerme el examen; entonces yo creo que, de hecho, para una persona que nunca se haya hecho un examen o nunca se haya puesto una vacuna va a recibir un montón de información, va a ser parte del proceso y va salir concientizado... cambias bastante tu pensamiento.*

La opinión que mostramos a continuación, ofrecida en un grupo focal, ilustra también cómo la información recibida en el contexto de la vacunación podría tener un impacto en las prácticas sexuales:

GF Gay: *Yo creo que de hecho asusta un poco porque... han dicho todo el tiempo que “ya, usa condón…”, por el caso del VIH, y ahora te dicen “bueno, no basta que uses condón, porque está este nuevo virus que te presentamos.” [que se transmitiría] ... solo porque rozaste y ni siquiera te vas a dar cuenta... [Otra:] Adiós previos, adiós todo eso [juegos eróticos].*

Para otras personas, el efecto de “desinhibición” en las conductas sexuales de otras personas era improbable, pues consideraban que mientras la vacuna sería solo contra el VPH, el condón podía proteger contra un rango más amplio de ITS:

ENT13 Most: *(E: ... algunas personas al vacunarse podrían pensar que ya no tienen por qué usar condón, ¿como crees que tú o tus amigos actuarían?) No pes, ¿la vacuna es para todas las enfermedades o solo para las verruguitas? (E: Es solo para las verrugas) En mi caso yo seguiría usando condón, porque la vacuna no me cuida de otras enfermedades ni del Sida, por ejemplo ¿o si?*

ENT15 Most: *(E: ¿Crees que tu conducta sexual cambiaría si te vacunaras?, porque a veces la gente dice, “bueno, ya me vacuné, ahora no uso condón”)* *Ah, ¿sí?, sí creo que pueda pasar en algunas personas, pero yo no, yo estoy seguro que si me vacuno igual seguiría usando condón porque hay otras enfermedades como el Sida.*

ENT9 Gay/Trav TS: *(E: ¿tú crees que algunas personas dejarían de usar el condón si se vacunan?) No. No, porque el condón te protege de todas las enfermedades. En cambio eh… esa vacuna solamente es para el papiloma. Que no tienes que… que no tiene nada que ver con el condón.*

ENT6 Trav TS: *... el hecho de que te vacunes tampoco no va a hacer [tener sexo] sin preservativo, porque entre todo eso hay una enfermedad que está el VIH.*

ENT8 Gay/Trav:*... de repente me pongo la vacuna y como otros maricones locas que vienen y se lo guardan [son penetrados] sin condón, el otro va a eyacular, doctor, pero obviamente va a perjudicar parte de mi… parte dentro de mi recto ¿no? (E: Ok ¿crees que tú usando la vacuna modificarías tu conducta sexual?) Doctor eh… de que yo puedo modificar, claro a mí me dijeron del sífilis, doctor (...) Me sentí tan mal (...) me cuidé tanto, doctor, de que ahora me cuido totalmente (...) porque nadie está seguro de nada en la vida, doctor, porque mañana más tarde… yo te coloco ahorita la vacuna y salgo afuera y me encuentro con un cuerazo le pongo el talco y le pongo el pecho, la comida y la bajada; de repente me voy a la discoteca, baila conmigo de repente se va al baño solo, ¡pla! le hacen un “güagüis”, le ponen algo, ¡pla! Le pasa algo, se lo guardan y me vengo a mi casa y ¿me siento seguro? No, doctor, no estamos seguro de nada y de nadie. (E: Eso quiere decir que no dejarías de usar condón a pesar de la vacuna.) ¡Ay doctor! Pero si mi mamá me trae los condones (...) no me siento seguro, doctor.*

GF Gay: *... también deben utilizarlo [el condón] porque no solamente se pueden infectar del VIH sino de varias cosas más.*

GF Trav TS: *No solamente hay esa enfermedad [infección por el VPH], hay varias. [Otra persona:] Esa vacuna es para una enfermedad específica, no… [Otra:] No para todos. [Otra] Sería tonto [dejar de usar condones].*

No obstante, unos pocos percibían que sí era posible que la vacunación produjera un cierto efecto de desinhibición, pero no en ellos mismos, sino en otras personas (ENT15 Most: “... sí creo que pueda pasar en algunas personas [que dejen de usar condones], pero yo no”). Es lo que pensaba, por ejemplo, una persona (gay), quien marcó una diferencia entre su propia conducta probable luego de recibir la vacuna, y lo que percibía como el posible comportamiento de otros. Por un lado, en lo personal, consideraba que al recibir la vacuna estaría protegiendo también a sus parejas sexuales, y que la vacuna no haría que deje de cuidarse debido a que existen también otras infecciones:

ENT2 Gay: *[Si recibiera la vacuna] estaría protegiendo a otra persona con la cual voy a estar ¿no?... sexualmente hablando*. (...) *Si yo me vacunaría, pues igual, me tendría que seguir cuidando. (...) Porque no solamente corro el riesgo de… contraer esa enfermedad, sino otras también.*

Sin embargo, esta misma persona opinó que la conducta sexual de otros, dependiendo del “criterio” de cada quién, podría verse influenciada por la vacunación en el sentido de que se sentirían protegidos (“más seguros”), y esto haría posible una mayor “irresponsabilidad” derivada de esa misma percepción de seguridad:

ENT2 Gay: *(E: ¿Y tú crees que la conducta sexual de la gente cambiaria si se vacunara con el papiloma virus?) Quizás... se van a sentir como más seguros ¿no? O también van a optar por hacer… un poco mas irresponsables porque ya… van a decir “bueno, estoy vacunado, entonces ya no voy a tener riesgo de contagiarme de eso...”*

Otra persona, por su parte, manifestaba también que su comportamiento sexual no se modificaría con la vacuna, pero percibía además que las prácticas sexuales de sus pares ya eran bastante riesgosas, y que esto no cambiaría mucho si recibieran la vacuna, que podría ayudar solo a que tomen un poco más de “conciencia” sobre los riesgos:

ENT10 Trav TS: *Porque como están vacunado creen que están ya prevenidos. Creen que ya no… con esa vacuna no les va a pasar nada y van a seguir con la misma conducta. (E: Es decir ¿no se va a proteger? ¿a eso te refieres?) De repente se descuidarían ¿no? con… o sea, tomarían un poco más de conciencia pero siempre va a haber un descuido.*

Al hablar sobre el posible impacto de una vacuna contra el VPH en la vida o la conducta sexual, no todos relacionaban a la vacuna con la prevención. Por ejemplo, para dos personas, la sensación de protección que ofrecería una posible vacuna se refería no a una percepción de seguridad ante la posibilidad de adquirir el VPH por las relaciones sexuales, sino a la idea de que la vacuna (o “medicamento”) ayudaría a “controlar” una infección que podría estar desarrollándose ya en el cuerpo de manera imperceptible, o “cortar de raíz” tal infección. Es decir, no asociaban la vacuna con la prevención, sino con la noción de tratamiento temprano:

ENT3 Gay: *(E: ¿Crees que tu vida se vería afectada si te la pones [la vacuna]?) Si es que lo tuviera [la infección], sí... porque es como una gripe, así una gripe, como una infección que le está creciendo encima ¿no?, Yo creo que si yo me vacuno contra la infección, voy a cortar de raíz la infección... un tratamiento. No va a ser de la noche a la mañana pero va a ser un tratamiento despacio, pero se va a tratar.*

ENT5 Gay: *(E: ¿Crees que eh… tu vida se vería afectada si te vacunaras?) No, no creo, no creo que se vería afectada [mi vida], porque si es un medicamento que va a controlar la enfermedad, no creo que pudiera afectar; al contrario, sería… sería prevenir, yo creo.*

**Percepción sobre quiénes deberían recibir la vacuna**

Al analizar las respuestas ofrecidas por los participantes al respecto de si la vacuna debía ser aplicada a quienes tienen un rol sexual “activo” o “pasivo” (la preferencia por penetrar o ser penetrado), encontramos que este asunto de los roles sexuales fue totalmente irrelevante. En realidad, estas personas percibían que el mayor riesgo reside no en asumir determinado rol sexual, sino en el hecho mismo de tener relaciones sexuales. De ahí que la respuesta más común ante esta pregunta haya sido que “todos” (activos y pasivos) deberían recibir la vacuna:

ENT2 Gay: *todos tienen sexo.*

ENT4 Gay: ... *los dos [activos y pasivos] están expuestos a… a estos tipos… corren el mismo riesgo*.

ENT5 Gay: N*adie está libre que digamos y [la infección] puede atacar a cualquiera.*

ENT7 Trav TS: *[Todos deberían recibir la vacuna] porque todos estamos expuestas a eso.*

ENT9 Gay/Trav TS: *Todos corremos el mismo riesgo*.

ENT13 Most: *Creo que todos [deberían recibir la vacuna], porque todos podemos contagiarnos ¿no?*

Una persona (gay TS), incluso, señaló que en las prácticas sexuales concretas de sus pares “no existe” tal distinción (activo/pasivo), y que “todos” deberían recibir la vacuna, pero en especial los hombres gay y quienes tienen comportamientos sexuales muy liberales o “promiscuos”:

ENT1 Gay TS: *Debería colocarse en todos. Porque no es definido, en el Perú no es definido una persona… en ningún, ningún lugar del mundo (...) Definido en el sentido que es “activo”, “pasivo”, no existe eso.* *(...) Creo que todos deben vacunarse, todos los que son gay deben vacunarse ¿no? porque la población gay, pues, es una población que es muy abierta en sus… en sus actos, en sus pensamientos y creen que... relajándose o haciendo diversas cosas que no deben hacer, creen que está bien, pues, y por lo tanto tiene que prevenir ¿no?... tomar conciencia. (...) Es urgente que lo utilicen y deberían vacunarse... porque la población es muy… crudamente, muy promiscuo ¿no? y… muy inconsciente, y yo me sumo a eso.*

También con respecto a este punto de quiénes deberían recibir la vacuna apareció la preocupación por las parejas sexuales, e incluso por las parejas regulares (mujeres) de aquellos hombres que tienen sexo ocasional con los hombres gay y las travestis. Es así que algunos planteaban que la vacunación debería extenderse a las personas heterosexuales, hombres y mujeres:

ENT6 Trav TS: *(E: ¿Piensas que la vacuna debería eh… colocarse principalmente en los gay, en las trans, en los pasivos, activos, en los mostaceros, en todos?) En general creo. Porque nadie... ¿quién no ha pasado por una relación que haya sido con una pasiva o una activo...? o hay parejas que son mujer y hombre y sus maridos buscan a las travestis y no… o sea, es un mundo ¿no? … que nadie puede salir, es una cadena... tienen sus mujeres y buscan a travestis y están con sus mujeres a la vez.*

ENT8 Gay/Trav:*... no solamente son los hombres y los gay, doctor, sino también en las mujeres porque usted sabe, el chico viene y me lo guarda a mí, me lo penetra a mí y se va por ahí y agarra a la enamorada y lo guarda; póngase que Dios no quiera yo tengo un… un malestar, una enfermedad y de repente se rompió el condón, el hombre contento, y yo por mí contento y feliz de sentir placer y no le digo nada, yo tengo y el hombre se va donde la mujer, como se siente seguro y confiado, como son pareja se lo guarda a la mujer [la penetra] y después…*

ENT11 Gay: [Debería aplicarse la vacuna] *En todos, porque los que la contagian son los hombres que la cogen de una mujer que la tiene posiblemente, porque a mí me dijeron que las mujeres eran quienes más las tenían.*

GF Trav TS: *A mi opinión no solamente a las travestis y a los gay [deberían aplicarles la vacuna], sino también a las otras [mujeres TS] porque hay cosas que utilizan el… [Otra persona:] El ano. (...) [Otra:] Porque también hay mujeres que también trabajan con el ano. [Otra:] Porque no necesariamente nosotras las travestis eh… corremos el riesgo, porque casi los modernos trabajan de hombres y… ellas son mas pasivas que nosotras. Tienen también el riesgo de contraer esa enfermedad.*

GF Gay TS: *[Deberían recibir la vacuna] Todos los modernos. [Otra persona:] Todos, creo. [Otra:] Los modernos más... el moderno porque cumple dos funciones... o las dos cosas a la misma vez, creo que es el que más riesgo tiene. [Otra:] Mira, un comentario así voy a darle ¿ya? Yo soy transformista, yo conozco de la plaza (...) obviamente de travesti me levantan puros activos, y solo un caso, más bien un hombre... pero cuando me toco en la plaza de moderno, más vi las verdaderas “vendimias” que tenían ahí, las verdaderas “uvas” [VG], más vi... en los modernos, en los hombres solamente vi un caso nada más.*

[Comentario: Como se puede apreciar en estas citas, las nociones sobre riesgos, poblaciones, redes sexuales y comportamientos que sostienen estas personas parecen ser más amplias y complejas que aquellas delimitaciones y clasificaciones que forman parte de los esquemas más comúnmente empleados en los estudios e intervenciones con HSH en Perú (activo, pasivo, gay, TS, etc.) Por ejemplo, algunos TS mencionaron los riesgos que estarían corriendo sus clientes y las parejas femeninas de éstos últimos. Varios hombres gay hablan también de algunas de sus parejas ocasionales (“activos” o *mostaceros*), y del riesgo al que se exponen ellos y sus novias o *enamoradas*. Sin embargo, los hombres bisexuales sin identidad *gay* u homosexual, que son parejas ocasionales de hombres *gay,* o clientes de TS *gay* y travestis, han recibido menos atención en los estudios o programas de prevención.]

CAPÍTULO 5

**ESTUDIO DE VACUNAS VPH: ACEPTABILIDAD Y RECLUTAMIENTO**

**Aceptabilidad de un estudio de vacunas contra el VPH con hombres**

Casi todas los participantes a quienes se les consultó si ellos mismos se involucrarían en un estudio de vacunas contra el VPH respondieron afirmativamente. Sin embargo, encontramos varias diferencias relativas a puntos de vista particulares sobre cómo son percibidos los objetivos del estudio, las motivaciones para participar en el proyecto, las expectativas de beneficios esperados, y las visiones sobre la aceptabilidad del estudio entre otras personas de la población.

En general, pocas personas manifestaron explícitamente que, según sus puntos de vista, el estudio serviría para “prevenir” o evitar nuevas infecciones:

ENT3 Gay: *Sería bueno [que se realice el estudio], como para… para no seguir infectando.*

ENT6 Trav TS: *Que está muy bien porque es… ¿cómo te digo?: es mejor que prevenir, que antes que lamentar ¿no? y si aún no… esto todavía no corre mucho, entonces hay muchas, muchas posibilidades que algunos se puedan cuidar, que lo puedan... contraer antes ¿no?*

La persona que tenía más información sobre el tema (por haber tenido VG y haber recibido tratamiento) opinó que la realización de un estudio de este tipo ayudaría a evitar no solo nuevas infecciones por el VPH, sino también el VIH:

ENT1 Gay TS: ... *así [realizando un estudio] se evitaría que las personas tengan más infecciones o que… evitarían que tengan VIH-SIDA.*

[Comentario: Mientras que para los especialistas y autoridades de salud puede haber una distinción clara entre un estudio y una intervención de salud orientada a la prevención, desde la perspectiva de la población objetivo no necesariamente se percibe tan claramente esa demarcación, por lo que un “estudio” a veces parece ser interpretado, a la vez, como una intervención de prevención. ]

Pero entre algunos otros persistían las dudas sobre si el estudio serviría para “prevenir” la adquisición del VPH y la aparición futura de VG, o para “curar” a quienes tienen ya la infección o las verrugas. Cabe señalar que estas dudas persistieron aún incluso luego de haber recibido una breve explicación sobre los objetivos del posible estudio:

GF Trav TS: [Hacia el final del grupo focal] *... ese proyecto que van a hacer, me imagino que van a hacer con personas que tienen la enfermedad, no con una persona sana ¿no? [Otra persona:] ¡No!, son para prevenir. (E: La vacuna es para prevenir.) Para prevenir no van a hacer eso, es para persona sana. [Otra:] Porque para entrar nosotros en el proyecto de herpes, tú tenías que tener esa [infección].*

Con respecto a los beneficios percibidos, podemos diferenciar dos actitudes: mientras que algunos resaltaban las ventajas que obtendrían en lo personal, ellos mismos (p. ej: sentimiento de seguridad, desarrollo de capacidades, saberse partícipe de los avances científicos), otros destacaban más los beneficios que el estudio brindaría a la población en general, o a determinado grupo en particular:

ENT4 Gay: *(E: ¿Qué piensas tú sobre... llevar a cabo esta investigación sobre vacuna en el Perú?) Están haciendo un bien para la salud de la gente... para nosotros los gay, entonces sí, sí, sí la tomaría, todo el mundo estaría preocupado por su salud...*

ENT9 Gay/Trav TS: *... a mí me parece perfecto ¿no? [que se realice un estudio], que alguna parte hay que agradecer a Dios que nos brinden esta habilidad ¿no? y que seamos parte también de la ciencia.*

En el capítulo anterior (cap. 4, apartado “Posible impacto de la vacuna...”), mostramos cómo el hecho de recibir la vacuna significaba para muchos la posibilidad de recibir información sobre el tema en el contexto de la vacunación, lo cual estaría siendo percibido como un beneficio real y tangible derivado de esa probable intervención de salud (vacunación), y como una motivación para recibir la vacuna. Pues bien, en el marco de las conversaciones sobre cuán aceptable sería un estudio de vacunas, volvió a aparecer la expectativa del acceso a información sobre el VPH y las VG. Esto era apreciado principalmente de dos formas: como la posibilidad de beneficiarse de información que recibirían individualmente, y como una forma de divulgar información hacia la comunidad.

(1) Por un lado, la perspectiva de que la participación en un estudio les permitiría a ellos, personalmente, recibir información sobre el tema, se vincula con la idea de que la información en sí misma es valorada como un beneficio.

[Comentario: El hecho de que este punto haya aparecido reiteradamente merece un examen especial, pues aún cuando desde la perspectiva de los investigadores este acceso a información podría ser visto como un beneficio secundario o *derivado* de la participación en el proyecto, para muchos de los participantes parece ser un asunto de la mayor importancia, que estaría funcionando como uno de los principales motivadores para tomar la decisión de involucrarse en el estudio, en ocasiones incluso más que la idea de prevenir una hipotética infección en el futuro:]

ENT5 Gay: *Para mí me parecería una idea que está bien, porque así de una y otra manera uno también ya tiene información y así se previene.*

ENT10 Trav TS: *Creo que es muy beneficiante [el estudio]. Para todos, porque nos va a informar, nos va a saber… nos va a informar y nos va a dar una prevención, una… una forma cómo prevenirnos y cuidarnos.*

GF Gay: *... supongo que la información dará un beneficio de estar todos en conjunto como comunidad para aprobar este tipo de vacunas.*

GF Gay: *Me parece bueno [que se haga un estudio]. (E: ¿Por qué?) Con todo lo que nos has informado (RISAS), aparte de asustarnos, creo que va a ser más conciencia para la comunidad del HPV.*

ENT2 Gay: *Pienso que es una iniciativa buena [un probable estudio sobre la vacuna], que es algo que va a ayudar no solamente a… a las personas que tengan esta información, sino a todos pues ¿no?*

GF Trav TS: *(E: ¿Cuál es la opinión que ustedes tienen acerca de realizar un estudio de papiloma virus acá en el Perú?) Me parece bien... te da buena información.*

[Comentario:] Siendo que no conocemos los antecedentes de los participantes, no podemos saber si las expectativas de estas personas tienen que ver o no con el hecho de haber participado previamente en estudios o intervenciones de prevención. En el grupo focal con travestis TS la mayoría indicó en cierto momento que había participado ya sea en estudios o en proyectos de prevención de VIH e ITS, por lo cual las opiniones o actitudes de estas personas con respecto a un nuevo estudio podrían no ser las mismas de quienes nunca han participado en proyectos anteriores. El caso que mostramos a continuación, por ejemplo, nos permite sugerir que las experiencias positivas de participación en investigaciones y programas de prevención y control de ITS estarían vinculadas a una mayor voluntad para participar en un nuevo estudio, como el de la vacuna contra el VPH:

ENT8 Gay/Trav: *[Habiéndose mencionado un estudio de vacunas] (E: Si estuviera disponible la vacuna en Perú, ¿tú te vacunarías?) Así como que han dicho en Bosé [¿discoteca Vocé?]... me dijeron: “hay un proyecto nuevo”, porque hace muchos años dijeron... “Oye, la gente, la 139...”, me vine corriendo, fui el cuarto participante y ahora me dijeron de nuevo, ahora me han dicho el Bosé, “Oye, hay una campaña contra el sífilis...”, [Un amigo le dijo:] “loca, anda tú que paras atrás de los condones, tú que paras atrás de las curaciones…”, me he venido corriendo, creo que he sido el décimo, no sé... Y la verdad que quisiera preguntarle a usted ¿cuándo empieza?, o voy a estar molestándole... (...) Tengo cuarenta; ya viví cuarenta y quiero unos años más doctor, no me siento seguro de la vida.*

Esta persona comentó además que ha apoyado anteriormente campañas contra el Sida, y que tiene una opinión favorable de instituciones como Impacta, que “ayuda” a la gente:

ENT8 Gay/Trav: *[Siempre ha apoyado...] la campaña contra el Sida, me encantaría la verdad, por favor pedirle... porque lo voy a buscar para contarle esto y felicitaría bastante a la institución y le pediría a [su amigo] que felicite también a la ONG, porque nunca dicen IMPACTA, toda la vida “Ministerio de Salud”, “el gobierno”, pero nunca dicen IMPACTA, “IMPACTA ayuda, IMPACTA atiende”, nunca dicen, pero sí me encantaría buscarlo para decirle que en IMPACTA están haciendo muchas cosas buenas que nos van a ayudar.*

En el capítulo previo (cap. 4) sugeríamos también que, en ciertos casos, la auto-percepción de estar en gran riesgo frente a las ITS tendría alguna relación con una mayor disposición para recibir la vacuna. Esta posible relación volvió a aparecer cuando se abordó el tema de la voluntad para participar en un estudio, por ejemplo en el caso de algunas travestis (TS):

ENT7 Trav TS: *Me parece bien ¿no? [que se realice un estudio], porque así se evitan muchas… no sé… para evitar esas cosas, las verrugas ¿no? porque las trabajadoras sexuales están más expuestas a eso.*

GF Trav TS: *No creo que las chicas así, nosotras que estamos esperando vamos a negar esa posibilidad ¿no? [de aceptar participar en el estudio], porque mayormente nosotras trabajamos con… con esa parte de nuestro cuerpo que es el ano, y como para que no salgan llagas u otras verrugas..*.

(2) Por otro lado, se percibía también la necesidad de difundir información sobre el VPH y las VG entre la población, pues se reconocía que estos temas eran ampliamente desconocidos. Si bien la gran mayoría de los participantes en entrevistas y grupos focales mostró su voluntad de participar en un probable estudio de vacunas, expresaron también una opinión mayoritaria respecto de que otras personas de la población aceptarían participar solo si se les ofrecía información, dirigida no solo a invitar a los posibles voluntarios, sino también a combatir los miedos y prejuicios que podría tener la gente. En torno a este punto, surgió nuevamente la preferencia por las formas interactivas y personalizadas de transmisión de información (consejería, “charlas”, “hablarles”), además de “campañas” informativas de mayor alcance:

GF Gay: *Creo que básicamente es dar información, educarlas. Creo que con eso... sí, la gente estaría preparada [para participar en un estudio].*

GF Gay TS: *(E: ¿Cuál crees que sería la mayor dificultad para que [otras personas] participen [en el estudio]?) Lo que pasa es que... la mayoría no conoce de ese tema... [Otra persona:] Claro. [Otra:] Habría que hacer una campaña [informativa].*

GF Gay TS: *... hay muchas personas más que también desconocen de este tema. [Otra persona:] Yo pienso que primero debería ser es la información ¿no?, estar informados y que la gente sepa a qué se atiene o qué puede producir eso ¿no?, hasta dónde puede llegar.*

Dos personas en un grupo focal (gay TS) pensaban que otros no querrían participar en el estudio porque percibían que muchos no tienen interés en cuestiones de salud (“no les interesa nada”), y que habría mucha “dejadez” de parte de la gente. Otra persona agregó que la aceptación del estudio por parte de la población no se daría de primera instancia por el solo hecho de invitarles (ENT10 Trav TS: “hablar de golpe tampoco aceptan”), lo cual sugiere que tal aceptación sería más bien el *resultado* de un proceso informativo. Así como esta persona, otras compartieron también la idea de que algunos hombres no querrían recibir la vacuna (en el contexto de un estudio), no solo por la falta de información, sino también por “miedo”, o por no querer enterarse de que probablemente tienen el VPH [quizás durante las pruebas de tamizaje que se realizarían para determinar quiénes pueden participar en el estudio]. En el caso de algunos TS, esa referencia al “miedo” se derivaba de experiencias previas con clientes, relacionadas con el temor a la prueba de VIH, o a la posible presencia de una infección en el/la TS o en el mismo cliente:

GF Gay TS:... *yo tengo muchos amigos por mi barrio... que tienen miedo a hacerse la prueba del Sida... le digo, “yo tengo un amigo, se saca la prueba, le dan condones…” (...) “Si salen con esa enfermedad, ellos te dan... tratamiento y todo”, yo les hablo; me dijo: “no, tengo miedo, yo tengo miedo.”*

ENT10 Trav TS: *(E: ¿Habría alguna razón por la cual no quisieran vacunarse? [hablando del estudio]) Por el miedo... a que de repente, no sé, inconscientemente lo puedan tener… O algunos sabiendo que de repente lo tienen ¿no? Porque a veces de repente no… no se han informado bien pero se han hecho pruebas. Entonces, de repente… tienen un riesgo y podemos... no sé, podrían hacer o no querrían hacérselo ¿no?*

GF Gay TS: *... yo creo la gente podría acceder o no, también de repente por vergüenza, temor... pero obviamente eso depende del tipo de medio de información que se maneje en cuanto a ese tema.*

GF Gay TS: *[Sobre las barreras para que otras travestis TS acepten participar en el estudio] El miedo. (E: ¿Miedo a que?) Miedo a que de repente por... el tipo de vida que llevan, eh… se sabe que a veces no se usa protección, porque no me va a decir a mí un chico que hace servicio sexual que siempre lo hace con condón, porque nos topamos... digo “nos topamos” porque yo también he sido de ese campo y me topo con cada persona que por dinero a veces uno se somete a eso y a mucho más... por eso... (...) El miedo es ese de saber que has cometido... has tenido un desliz, y de saber lo que puedes tener... yo creo que por miedo [es] por lo que no lo harían...*

GF Gay TS: *[Sobre si sus clientes aceptarían recibir la vacuna o participar en el estudio] No, creo que no. [Otra persona:] Los clientes no van a venir, te van a decir... tú le dices “¡vamos para que te hagas un examen!”, te van a decir… [Otra:] Ya, si tuviera confianza… nos llevaríamos bien, yo creo que sí... [Otra:] Mayormente te metes con un cliente que viene y sale y ya…[Otra:] En realidad la enfermedad los clientes no saben… porque ya te toman como que tú eres una persona que esta delicada… [Otra:] Como que tú le has contagiado. [Otra:] Hace poco yo le dije a un cliente “¿hay que hacernos la prueba?”, y me dijo “¿Por qué?, piensas que… ¿tú estás con esa infección?”, “no”, le dije, porque “siempre es bueno hacérsela”, y me dijo “ya, la hacemos”, y yo tengo las pruebas así en la mano.*

Algunos participantes en grupos focales manifestaron que podría haber alguna resistencia hacia el estudio de parte de personas externas al “ambiente” gay si se llegara a establecer la idea de que el VPH es una infección que afecta a esta población, o que el estudio está dirigido solo a hombres homosexuales:

GF Gay: *Yo creo que... cuando se llegue a dar una especie de taller o informativo a la comunidad creo que los heterosexuales van a oponerse de un modo a ese tipo de vacunas, porque de repente van a adjudicar nuevamente “el VPH es de la comunidad…[gay].” (...) hay gente que va a salir con este tipo de rollos, también creo que, como dices, educarlos… [Otra persona:] O sea, creo que [habría que] educarlas... darle información, charlas, llevarlos a diferentes sitios…*

GF Gay TS: *Pero otros no, no, no quieren venir, o sea, porque no están en el ambiente “es puro cabros, es para cabros”… [Otra persona:] Ah… eso es lo que dicen siempre, eso es lo que dicen. [E: ¿Qué cosa?] Eso es para los cabros…*

Con respecto a la decisión sobre participar o no en el estudio, la mayor parte de las personas mostraron su voluntad para participar, sin presentar mayores reparos al respecto. No obstante, unos pocos, aún cuando indicaron que en principio sí desearían participar, manifestaron también que necesitarían ser informados sobre cuestiones como los riesgos de recibir la vacuna, o sus posibles efectos adversos:

ENT2 Gay: [Participaría] *... claro, este… tomando en cuenta todos los… los beneficios, digámoslo así, que yo no corra ningún riesgo ¿no? creo.*

GF Gay: ... *otra de las barreras puede ser también... el temor de las… los efectos secundarios que pueda tener [la vacuna].*

GF Trav TS: *(E: ¿Ustedes participarían en este estudio de papiloma virus?) Obvio. (E: ¿Sí? ¿Todos?) [Otra persona:] Claro. (E: Sus amigos, conocidos ¿participarían de este estudio?) [Otra:] Quizás... explicándoles. [Otra:] Explicándoles.*

Algunas preguntas específicas sobre el estudio fueron: cuándo se iniciaría; si representaría algún costo para los participantes (GF Gay: “la gente pensaría si es barato o si es caro”); y qué institución (o “empresa”) realizaría el estudio. Solo en el grupo focal con travestis TS se expresaron ciertas dudas que fueron expresadas desde las nociones “riesgo” asociado al estudio, de “experimentación” con algo que no se sabe si funcionará, y la “utilización” de los voluntarios (para ver si funciona la vacuna), aunque durante la misma discusión surgieron también opiniones en el sentido de confiar en la buena voluntad de los investigadores:

GF Trav TS: *(E: Posiblemente... estemos iniciando un estudio de vacuna.) O sea, ¿recién lo van a hacer? [Otra persona:] Utilizan a las mujeres para cáncer del cuello uterino, ¿van a utilizar con unas chicas trans…? [Otra:] ¿Van a experimentar o que? ¿Para que vean la reacción? [Otra:] Claro, así como el herpes ¿no? aciclovir… [Otra:] O sea, recibirá una persona pero estaría bien segura si es que resulta la ampolla ¿no? porque a veces hacen experimentos. (...) [Otra:] Más… más esperaría pruebas, esperaría si resulta o no. [Otra:] Mucho riesgo. [Otra:] Pero no creo que… digamos, los investigadores o los científicos al probar, al entrar los voluntarios a probar eso, no creo que se haría correr riesgo a uno.*

También en este mismo grupo focal con travestis TS se expresó una preocupación por la forma y el lugar de aplicación de la vacuna, debido a que quienes tienen implantes de siliconas no podrían recibir inyecciones en las nalgas:

GF Trav TS: *Hay una pregunta que quiero hacer ¿la vacuna donde va a ser puesta, en el brazo o en la nalga? Porque si es en la nalga no nos podemos poner en las nalgas. [Otra persona:] No nos podemos poner [aludiendo a las siliconas]. [Otra:] ... por ejemplo casos de chicas que tienen sífilis y que se les tiene que aplicar las dosis de penicilina se les pregunta si tienen silicona en el trasero, porque si tiene silicona no se le puede aplicar ahí la penicilina, y se le aplica en el brazo ¿no?*

Solo en el grupo focal con hombres gay TS se mencionó el tema de las reacciones del entorno social frente a la participación en el estudio. Al respecto, varios opinaron que sus familiares los apoyarían en su decisión de entrar al proyecto, principalmente porque percibían que sus familias se preocupaban por su salud:

GF Gay TS: *(E: ¿Creen ustedes que... tendrían dificultades o algún cuestionamiento por parte de sus amigos, [o] familia para participar de un estudio de este tipo?) No. [Otra persona:] Por mí, no. [Otra:] Mi Mamá siempre dijo “mientras te cuides y hagas lo correcto y lo debido…” [Otra:] Igual mi hermana me dijo “hagas lo que hagas, usa condón.” [Otra:] Mi familia me apoya en toda esas cosas…*

Hubo quienes mencionaron que el personal del estudio debería brindarles un trato adecuado y respetuoso. Este tema fue planteado por algunas personas con referencia a experiencias negativas previas en servicios de salud, en los que no les agradó verse cuestionados por sus prácticas sexuales, o que les trataran distinto por su aspecto físico o su identidad sexual. Al parecer, más que una expectativa por recibir un trato especial, lo que les interesa es recibir un trato respetuoso, en el que no se les estereotipe o se les juzgue por sus conductas o identidades sexuales:

GF Gay: *... no se tiene confianza alguna [en el centro de salud] porque los médicos son unas mierdas, los enfermeros también, o sea, te van a atender como cualquier cosa y te vas a ir y te van a mirar mal*.

GF Gay: *Me hice un chequeo hace tres años y este… (...) el médico me dijo que tenía el intestino demasiado largo… y que tenía que tener una dieta... para evitar que me pueda dar a futuro cáncer de colon, y yo por cuando escuché “cáncer de colon”, le dije... no sé, tenía de hecho vergüenza decirle “¿sabes qué?, a mí me dan a veces [le penetran] y quiero saber qué riesgos tengo”, y como que el doctor entendió a lo que iba la pregunta y me dijo que no, que eso no se debía hacer, que era maltratar mi cuerpo, y que jamás y que por nada del mundo, y me dio una charla enorme de físicamente por qué no era apropiado... tener sexo anal.*

GF Gay: *... en el año 2008, ya, me fui a hacer una prueba rápida... de VIH en el Peruano Japonés, y me tocó un chinito. El chinito me dijo así: “¿Por qué se hace la prueba?”, le digo “porque quiero saber mi diagnóstico, porque he tenido riesgo”, “ah ya, ¿tú eres mujer o hombre?”, y yo: “mmm ¿mujer o hombre? ¿no entiendo?”, o sea… me dijo: “¿Cómo que no entiendes?”, y yo: “mmm”, como que… hubieron muchos, como que no entendía… o sea, sí entendía pero me hacia el loco… “¿A qué se refiere?”, “Ah no, yo pensaba que eras este… homosexual”, y yo le dije: “¿Y qué tiene de malo que sea homosexual o no sea homosexual?” O sea, obviamente que yo sí estaba molesto, me molestó la pregunta.*

GF Trav TS: *Más el trato también, digamos, acercarse a la persona con buena educación y demostrar que tenemos confianza entre ellos y apoyarlas, porque algunas personas [personal de salud] no saben lo que es el sexo anal.*

GF Trav TS: *(E: ¿Creen que el personal encargado de colocar la vacuna a las trans es importante para determinar el éxito de la vacunación?...) Pienso de que esa pregunta está como que de más, porque supuestamente si ya se va a vacunar la persona de alguna manera cree que es necesaria, yo pienso de que como en cualquier lugar necesita un buen trato...*

GF Gay TS: [Algunos profesionales de salud]... *por ver cómo somos, se confunden, o creen que andamos con un letrero puesto... no es así. (...) Cuando recién entré a IMPACTA por primera vez, el doctor me revisó, me dice: “pasa”, o sea, su amabilidad me gustó ¿no?, porque obviamente uno se siente bien, pero cuando toco revisarme ya… no me gustó nada la forma como llegaba hacia a mí. Yo dije: “doctor, ahí no más lo dejamos”; “no, pero si eres gay”, me dijo. ¿Por qué en ese examen, por qué ese comentario? “¿Cómo? Yo puedo ser gay pero yo no ando con un letrero puesto, que necesito 24 horas del día, no se confunda”, le dije... Dije, “bueno, lo dejo que pase”, pero preferiría... [que] me atienda una doctora.*

GF Gay TS: *... cuando fui la primera vez [a un servicio de salud], me atendieron rápido; fui la segunda vez, se demoraron como cuatro horas para darme la atención. (E: Ok, eso tiene que ver con la cantidad de personas que se atienden en un lugar.) Yo fui, pero había una persona, otra persona, así como… puras prostitutas, caseritas eran en el hospital, y le decían “Hola ...”, y se entraban así y me tenían en la cola, en la última y yo había llegado primero. Y se metían, estas conchuditas le decían, tenían que esperar.*

GF Gay TS: *... el personal [de salud] está capacitado para que te vayan a atender, no para que se vaya a saber tu vida sexual.*

Cuando se abordó este tema, una persona mencionó cuán distinta había sido su experiencia en servicios ofrecidos por algunas organizaciones trabajan con población “gay”, en las que percibió un mejor trato, que le era brindado por personas también “gay”; y alguien más en otro grupo focal señaló también que le agradaba el trato que había recibido en otro estudio:

GF Gay: *Un gay [parte del personal de salud] como que… dice “ya, bueno”, habla con la persona, como que te entiende ¿no? Por ejemplo, yo... enfermera, por ejemplo las de IMPACTA, esos son bien tratables, o sea te hablan bien, te conversan bien... por ejemplo los consejeros de Epicentro te conversan bien, te hablan bien; los consejeros voluntarios te conversan porque son obviamente personas gay.*

GF Gay TS: *Yo participo en IPREX. [Otra persona:] Me gusta cómo me tratan porque ya me conocen, inclusive me dicen “China...”*

**Opiniones y recomendaciones sobre estrategias de reclutamiento**

La mayoría de los participantes brindó sus puntos de vista sobre lo que les parecía serían las mejores formas de convocar a voluntarios para un posible estudio de vacunas. El punto principal fue, nuevamente, el de la difusión de información sobre el VPH y las VG, dado el gran desconocimiento que existe entre la población sobre estos temas. Debido a esto, varias personas opinaron que la información a ser divulgada desde el proyecto debería estar enfocada no solo en el estudio o la convocatoria a los posibles voluntarios, sino que debería contemplar también una suerte de campaña informativa de mayor alcance, dirigida a sectores más amplios, para promover un mayor conocimiento sobre el VPH y las VG, y para establecer varias ideas: que el VPH y las VG son un importante problema de salud; que afectan también a hombres, y no solo a mujeres; y que esta infección puede propiciar una mayor diseminación del VIH. Algunos, incluso, sugirieron que esta información más amplia debería ser divulgada antes de implementar las acciones de reclutamiento:

ENT1 Gay TS: *(E: ¿Cuál crees tú que sería la forma de motivar o alentar a la gente a participar en este estudio?) Creo que la forma sería... sacándoles las vendas de los ojos, diciéndole que [no] solamente a las mujeres les dan el papiloma, pues, y que es un factor para que… es una causa el papiloma para que te dé el VIH pues ¿no?… sin que nadie se dé cuenta pues, porque te puedes herir el pene o puedes tener este… heridas en el ano…*

Como señalábamos previamente, encontramos una preferencia por las formas personalizadas e interactivas de comunicación (cara a cara: “consejerías”, “hablarles”, “orientarles”) para las estrategias de aproximación a la gente con miras a invitarles a participar en el estudio:

ENT2 Gay: *... si tú le hablas de esta forma, como tú me estás diciendo, claro que van a aceptarlo porque primero van a conocer de que es el papilo… el virus del papiloma...*

ENT6 Trav TS: *[La gente aceptaría participar si se les explica el estudio] en el modo que tú me has explicado.*

Con respecto a la información sobre el VPH y las VG que habría de ser dirigida a sectores más amplios de la población, algunos sugirieron la implementación de “campañas informativas” y el empleo de medios masivos, como la televisión y la prensa escrita. Algunos entrevistados propusieron además el uso de medios electrónicos, como el internet y las redes sociales (como *Facebook* y *Hi5*).

ENT8 Gay/Trav: *[Si él estuviera a cargo de la promoción del estudio...] Para empezar, lo pondría como un spot en el canal y después este… a la prensa escrita porque no solamente yo voy a ir a una esquina, a un campeonato de vóley y a decir “oe, maricón, hay una campaña”, la verdad que iría a la prensa, al canal, a los periódicos y le diría: “ayúdanos a hacer esta campaña para que todo el pueblo sepa.”*

En relación con los lugares que deberían ser visitados por los reclutadores del estudio, la mayoría mencionó lugares en que se realizan encuentros homoeróticos y en los que ocurre sexo ocasional (“un encuentro y nada más”). Una persona (ENT1 Gay TS) mencionó que esto puede suceder, por ejemplo, en algunas cabinas de internet “exclusivamente gay” (ubicadas en el Centro de Lima y en SMP, cerca de la UNI), que él considera “el principal foco de infección”. Esta misma persona refirió que, precisamente porque en esos lugares el sexo es ocasional y rápido, es menos probable que las personas se detengan a examinar si su pareja tiene VG en el pene o en el ano:

(ENT1 Gay TS) *... las personas se van ahí, tienen un encuentro y nada más pues, pero muy poco son los que revisan el órgano de la otra persona ¿no? de tu pareja sexual activa. O sea, muy pocos son los que te agarran el pene, cuando está erecto y ves si tienes una verruga genital o te revisan el ano cuando están a punto de penetrar a la persona.*

En el grupo focal con jóvenes gay TS se indicó que el reclutamiento se podría hacer también en las zonas de comercio sexual que ellos frecuentan, incluyendo los “cines porno” y los locales que tienen “cuartos oscuros”, mencionando también que en estos lugares la obscuridad misma impide tomar medidas de precaución ante posibles riesgos (y ver si una pareja ocasional tiene o no VG):

GF Gay TS: *(E: ¿Qué otros lugares? Ustedes que andan más en el tema, por ejemplo ¿Dónde?)* *¡Los cines!, ¡los cines! [Otro:]* *El “Cine Tauro”…* *¿quién no para en el “Cine Tauro”, no se ve cómo entran? (...) (E: ¿Dónde radica el mayor problema para que los chicos que se dedican al trabajo sexual no se protejan?) En los cines, porque son oscuros. [Otro:] Si, en los clubes donde también hay cuartos oscuros. [Otro:] En los cines me parece, me parece más riesgo para contagiar, es un lugar oscuro.*

Varias otras personas mencionaron que se podría invitar a las personas, empleando material informativo impreso (como “folletos” y “volantes”), en los “videos porno”, bares, discotecas y “clubes de ambiente” ubicados en diversos puntos de la ciudad. Algunos volvieron a plantear la conveniencia de emplear imágenes de VG en los materiales promocionales, para utilizar el “temor” como una forma de generar “conciencia” entre los posibles voluntarios, motivándoles a que piensen en lo que les podría ocurrir si llegaran a tener la infección:

ENT6 Trav TS: *yo veo las imágenes, cualquiera se asusta...*

ENT13 Most: *(E: Si... te dijéramos que nos digas cómo invitar a chicos como tú, ¿cómo lo harías?)* *Por charlas, afiches con fotos como esas y decirles que es para prevenir.*

GF Gay: *(E: Ustedes dicen de que han visto fotos y les ha impresionado, pero ¿creen que sería una forma de incentivar a la gente...?) Creo que por demás… es útil. [Otro:] Sí. [Otro:] El temor es útil. [A otro, un promotor le había dicho que sería bueno emplear fotos para] que se traumen las locas, que eso es lo que van a tener si es que no se cuidan. [Otra:] Tiene que haber polémica... [Otro:] Para que la gente también tome conciencia.*

[Comentario:] Es muy posible que las ideas propuestas tengan relación con experiencias previas de haber sido contactados por promotores de intervenciones o estudios anteriores. Una persona, por ejemplo, recomendó emplear un esquema en el que los promotores debían emplear materiales informativos para primero contactar a los posibles voluntarios en los lugares de socialización, y alentarles a que visiten “clínicas” para que los “consejeros” les brinden más información sobre el VPH y las VG, antes de invitarles a participar en el estudio:

ENT3 Gay: *Primero, si tendría el material correspondiente, lo que son folletos o revistas grandes; cómo poder causar… o cómo poder acabar esta enfermedad, y segundo ¿no? invitándoles, no de frente a vacunarse ¿no?, invitándoles a que vayan a la clínica para que el consejero o consejera le hable acerca de la vacuna y acerca de esta enfermedad; de repente yo no voy a poder tener todo el conocimiento que el consejero o la consejera tiene.*

Esto último guarda cierta relación con la opinión de otra persona, quien consideraba que la gente normalmente no aceptaría participar de primera instancia, desde el momento inicial de contacto (ENT10 Trav TS: “hablar de golpe tampoco aceptan”), sino que la aceptación sería resultado de un proceso de transmisión de información.

Una persona mencionó, además, la posibilidad de contactar a la gente en los centros de salud:

ENT2 Gay: *... se mueven… por ejemplo eh… en discotecas ¿no? Pero en los mismos centros de salud de VIH también, las personas que se van a atender... les hablaría sobre este tema.*

Se señaló también que a los posibles voluntarios se les debería hablar sobre el carácter gratuito del estudio desde un inicio, lo cual podría motivar a la gente a participar, sobre todo si perciben a la vacuna en sí misma como un beneficio que irían a recibir.

Solo una persona recomendó promocionar el estudio como una forma de ayudar a la gente a saber si tienen o no el VPH, y si están a tiempo de prevenirlo (ENT2 Gay: “sobre todo te vas a dar cuenta si es que estas infectado o no”).

Los datos sugieren también la conveniencia de evaluar posibles estrategias diferenciadas para subpoblaciones como la de las travestis que se dedican al comercio sexual. Esto lo percibían, por ejemplo, algunos de los participantes gay en un grupo focal:

GF Gay: *[Sobre las “trans”] Hay que tratarlas bien. [Otra persona:] Es una población que es bien… un poquito difícil de llegar, porque son como que medias… que toman a la defensiva las cosas. [Otra:] Media arisca.*

Al respecto, varias travestis TS manifestaron que se les podría contactar en “discotecas” y en los lugares de comercio sexual. Aunque una de ellas reconoció que esto podría interferir con sus actividades, otra señaló que siempre tienen momentos libres en que conversan entre ellas, y sugirió que, en esos casos, la estrategia más eficaz sería lograr que ellas mismas divulguen entre sus pares la información sobre el tema, o sobre el estudio, antes que intentar contactarlas directamente una por una:

ENT7 Trav TS: *[en las calles]... siempre hay un grupito, que… que… que tiene un tiempo libre ¿no? que se dedica a jugar... y entre nosotras nos pasamos la voz, nos pasamos la voz y así porque en realidad, dudo que te hagan caso porque todas piensan en trabajar.*

Otras travestis mencionaron que se les podría invitar también en los “hoteles” o casas en que viven (con la autorización de los dueños).

Una travesti, quien había tenido VG, lanzó otra idea interesante. Dijo que las personas como ella, que conocían del asunto y se sentían libres de poder hablar del tema con otros, podrían contribuir con el estudio explicando el tema a sus pares y conversando con ellos para que “tomen conciencia” y acepten recibir la vacuna:

ENT7 Trav TS: *Explicarles, por ejemplo yo… yo les cuento a mis amigas lo que yo tuve ¿no? y por eso les comento a ella que se haga ver porque eso es serio. Y eso va creciendo ¿no? Y no sé, eso sería una de las maneras de que ellas tomen conciencia y se hagan… si existe la vacuna se la pongan.*

[Comentario: Esta propuesta podría funcionar incluso si no implica que los pares promotores tengan que revelar que tienen o han tenido VG, para que no se expongan a posibles episodios de estigmatización.]

En relación con esta última cita, surgió también entre los hombres gay TS una voluntad por colaborar con el estudio difundiendo información entre sus pares, e invitándolos a participar:

GF Gay TS: *(E: ¿Cómo le dirían a sus amigos para que participen en un estudio de papiloma virus?) ... así como comentarios, como conversando le diría “oye, ¿sabes qué?, mira, yo participo en tal estudio eh… trata de las siguientes enfermedades y mira es así es asa y… te conviene, ya, porque ¿quién no quiere cuidarse y prevenir sobre todo?” [Otro:]… que venga a participar de la prueba, que lo van atender. (E: ¿Cómo lo convencerías?) En realidad, no convencería. [Otro:] Ya eso depende de él, si acepta o no, también.*

Una travesti consideraba que debían ser las personas más “experimentadas” de la misma comunidad quienes se encarguen del reclutamiento entre sus pares, pues para otras personas (externas a la comunidad) esto podría resultar algo más “complicado”:

ENT9 Gay/Trav TS:*... es un poco complicado para uno, yo de decir, le pudiera decir [a sus pares] pero, o sea, que ellas decirlos animar, es un poco duro, es un poco difícil si para ellas, pero sería para personas experimentadas que tienen más clara sus temas.*

Para otra travesti, la aceptación del estudio por parte de sus pares pasaría por un esquema de reciprocidad: si la institución que realiza el estudio les ofrece algo valorado para ellas (p. ej. capacitación en algún tema que les interese), esto haría más factible que la población acepte participar, lo cual puede ser visto como una forma de “colaborar” con el proyecto, retribuyendo el beneficio recibido. Se mencionó, por ejemplo, la implementación de “talleres” de capacitación en labores productivas, espacios que servirían al mismo tiempo para transmitir información sobre salud y sobre el estudio:

ENT9 Gay/Trav TS:*... sería un taller, porque un taller se encuentra en formación, se encuentra manualidades. Se encuentran más preparadas, eh… y poco a poco llegar a ellos.*

[Comentario: Este esquema de reciprocidad podría funcionar principalmente con aquellos grupos que parecen ser los más marginalizados, como las trans, y tiene que ver también con la percepción sobre los beneficios palpables que recibirían por su participación (información, capacitación, trabajo, dinero, regalos, prestigio, etc.)]

A diferencia de otros grupos, que se inclinaban por formas personalizadas de comunicación (como la “consejería” individual, por ejemplo), entre las travestis notamos una preferencia por estrategias interactivas grupales, como el ofrecimiento de “charlas informativas” en determinados locales de reunión, ofrecidas por especialistas que puedan absolver sus dudas y que sean sensibles a su identidad sexual, empleando videos, y compartiendo refrigerios con las travestis asistentes [siendo esto último, nuevamente, un elemento del esquema de reciprocidad]:

ENT10 Trav TS: *... los reuniría, les pondría videos, depende, alquilaría un local, no sé, haría un “compartir”, de alguna de esa manera para capturarlo ¿no? o capturar su atención y llamarlos a que vengan.*

GF Trav TS: *(E: ¿Qué convencería a la gente para que participe en un estudio como este?) Explicándole detalladamente… [Otra:] Lo riesgoso que es ¿no? [Otra:] Podría ser en un taller... con personas que realmente son preparadas, que puede ser un doctor, un psicólogo… para que puedan tener más conocimiento...*

GF Trav TS: ... *más que todo que te traten como mujer.*

Finalmente, en el grupo focal con travestis TS se habló sobre una posible barrera para que la gente se anime a participar, barrera relacionada con probables resultados inesperados en los análisis de sangre, y también (aparentemente) con el miedo a ser estigmatizados por otras personas:

GF Trav TS: *(E: ¿Cuáles creen [serían] las razones por la cuales no participarían?)* *Por temor…[Otra:] A veces de repente no quieren hacerse análisis de sangre por temor también. [Otra:] ... por ejemplo, digamos ya no quiero participar... ¿a qué me voy a ir? Es que el doctor, el chico va a darse cuenta (...) Mayormente, siempre… van a revisar ¿no? El pene, el este, el otro… le va a mirar o que de repente salgo con eso [VPH-VG] y las chicas van a pensar que estoy con eso (...) A veces los maricones no quieren y no quieren por eso.*

**CONCLUSIONES**

- El virus del papiloma humano (VPH) era ampliamente desconocido entre los participantes en el estudio. Las pocas y únicas personas que tenían algún conocimiento sobre este virus eran aquellas que reportaron tener o haber tenido verrugas genitales (VG), aunque este conocimiento por lo general no era muy profundo.
- Las VG, en cambio, sí eran conocidas por varias personas (pero no por muchas), ya sea por que las tenían o habían tenido ellos mismos, porque las habían visto alguna vez en sus parejas (o clientes, para los TS) al momento de tener relaciones sexuales, o porque habían oído hablar de ellas. En relación con esto, fueron las personas que se dedicaban al comercio sexual quienes señalaron más frecuentemente haber visto VG o haber oído comentarios sobre ellas (aunque a veces no quedaba claro si se trataba de VG o a lesiones causadas por otras ITS); mientras que los jóvenes bisexuales (*mostaceros*) tenían menos referencias al respecto. En general, era muy limitada la información de la que disponían sobre las causas de las VG, sus formas de transmisión, o su tratamiento.
- Entre quienes tenían o habían tenido VG, los sentimientos predominantes asociados al descubrimiento de VG en sus genitales fueron la extrañeza, el miedo y la incertidumbre, derivados principalmente del desconocimiento sobre las posibles causas de esta anormalidad en sus cuerpos, que producían perturbaciones en el bienestar emocional, molestias físicas, e impactos negativos en el ámbito de la sexualidad (p. ej. “vergüenza”, situaciones embarazosas, o miedo a transmitir la infección a otros). Las demás personas (que no habían tenido VG), por su parte, mostraron sobre todo actitudes de perturbación y rechazo hacia las VG, a excepción de las travestis, quienes parecían verlas con algo menos de rechazo, pero sí con preocupación y extrañeza.
- El tema de las VG no suele ser tocado entre pares o en las relaciones de pareja. Las actitudes de rechazo hacia las VG y hacia quienes las tenían definen formas de estigmatización que llevan a las personas con VG a desarrollar mecanismos o estrategias para ocultarlas (p. ej. teniendo sexo a oscuras), o para justificar su presencia apelando a argumentos en los que presentan a las VG no como el resultado de una infección adquirida (sexualmente), sino como una aparición espontánea o derivada de eventos y situaciones que escapan a la voluntad de las personas (p. ej. diciendo que son “hemorroides”, quemaduras, cicatrices, lunares, etc.)
- Tanto el desconocimiento sobre el VPH como la vergüenza y el estigma asociados a las VG dificultan el acceso a información y servicios de salud. En varios casos, las VG son extirpadas de manera artesanal. En otros casos, algunas personas llegan a acceder a tratamientos médicos para sus VG cuando logran vencer sus sentimientos de vergüenza y por lo general con el apoyo de algún miembro de sus redes sociales de apoyo (amigos, promotores de salud).
- Solo algunas de las pocas personas que habían tenido VG sabían de la existencia de vacunas contra el VPH que son empleadas con mujeres. En general, el concepto de “vacuna” tenía varios significados: mientras que unos pocos lo asociaban con la “prevención”, para muchos otros las ideas de “vacuna” o “vacunación” se referían ya sea a “curar”, “tratar” o “controlar” una enfermedad, o a prevenir que se manifieste físicamente una infección que posiblemente ya se tiene y que aún no es visible.
- Notamos entre los participantes en el estudio una actitud positiva hacia la vacuna contra el VPH. Casi todos dijeron que aceptarían recibirla ellos mismos. No obstante, no todos creían que sus amigos, pares, o en general la población, tuvieran la misma actitud de aceptación, principalmente por el desconocimiento y la escasez de información sobre el VPH y las VG.
- La actitud positiva hacia la vacuna tenía relación principalmente con la expectativa de poder recibir información sobre el VPH y las VG en el contexto de la vacunación, y en menor medida con la posibilidad de prevenir (o “curar”) una futura infección. Para algunas personas, la mayor voluntad por recibir la vacuna parecía estar asociada con la *percepción del propio riesgo* frente a las ITS. Muy pocas personas mostraron dudas o reparos por posibles riesgos para la salud vinculados a la vacuna. En relación con sus pares u otras personas de la población, muchos consideraron que estas personas podrían aceptar recibir la vacuna solo luego de ser informados apropiadamente sobre estos temas, ya que el desconocimiento o el miedo podrían conducir a reacciones de rechazo hacia la vacuna. Al respecto, los jóvenes *mostaceros* fueron los más escépticos respecto de si sus pares aceptarían vacunarse. Se destacó el uso de imágenes o fotografías como una forma de motivar a la gente a “tomar conciencia” de lo que les podría ocurrir en el futuro si se infectaban con el VPH.
- Sobre las reacciones de sus entornos sociales si ellos se vacunaran, encontramos entre los participantes una voluntad por compartir el tema con los familiares, más que con los amigos. Varios percibían a sus redes familiares más cercanas como un ámbito de seguridad y protección, y no tanto de cuestionamientos, aunque unos pocos manifestaron sentirse independientes y autónomos en sus decisiones sobre su salud, por lo que le otorgaban poca importancia a las probables reacciones de sus familiares o amigos. Muy pocas personas pensaban que podrían ser estigmatizadas por recibir la vacuna.
- La mayoría de los participantes no percibía que recibir la vacuna contra el VPH vaya a significar un cambio en sus prácticas sexuales, en el sentido de una posible “desinhibición”. Por el contrario, la mayoría consideraba que la vacunación contra el VPH podría tener un impacto positivo en sus vidas, ya sea porque recibirían y manejarían información sobre el tema (lo cual les ayudaría a “protegerse” más para prevenir posibles infecciones), porque les llevaría a pensar más en los posibles riesgos a los que se exponen al momento de tener relaciones sexuales, o porque existen varias otras ITS de las que tendrían que seguir cuidándose.
- Sobre el tema de si la vacuna debería ser aplicada preferentemente a quienes desarrollan el rol sexual pasivo o activo, encontramos que el asunto es totalmente irrelevante, ya que el riesgo de adquirir la infección por el VPH es asociado con el hecho mismo de tener relaciones sexuales, y no con determinado rol sexual.
- Desde la perspectiva de los participantes en el estudio, un proyecto de investigación de vacunas contra el VPH en HSH sería altamente aceptable. Casi todos señalaron que ellos mismos se animarían a participar, y algunos mostraron incluso su disposición para colaborar con el estudio motivando a sus pares a entrar al estudio. No obstante, consideraron que la posibilidad de que también otras personas se involucren pasaba primero por una adecuada difusión de información, y por la implementación de estrategias apropiadas de reclutamiento. Al respecto, muchos expresaron sus preferencias por formas personalizadas e interactivas de comunicación (“cara a cara”, p. ej. “consejería”), aunque las travestis se mostraron más a favor de estrategias grupales (como “charlas”) que contemplen formas de reciprocidad y beneficios concretos para ellas. Sobre este punto, en general, se percibía que la misma perspectiva de recibir información especializada sobre el VPH y las VG era vista como un beneficio real, el cual a veces era incluso más valorado que la misma posibilidad de prevenir una hipotética infección que podría ocurrir en el futuro. Los lugares más mencionados para la aproximación a los posibles voluntarios fueron aquellos en que se producían contactos que derivaban en sexo ocasional.

**RECOMENDACIONES**

- Dado que el VPH y las VG son asuntos largamente desconocidos para la mayoría de HSH, las estrategias de información y comunicación cobran enorme relevancia para la adecuada marcha de investigaciones o intervenciones futuras en estos temas, tal y como lo perciben y expresan explícitamente los participantes en este estudio. Es por eso que hemos seleccionado una serie de interrogantes que surgieron a lo largo de las entrevistas y grupos focales, para que sean tomadas en cuenta al momento de diseñar mensajes, actividades informativas, entrenamiento de promotores, modelos de consejería y estrategias de comunicación, para anticipar las preguntas que podrían surgir desde los posibles voluntarios:
  - Forma de transmisión: Si las verrugas se pueden transmitir de una persona a otra; cómo puede ocurrir esto; si puede ocurrir por medio de “roces” o la “saliva”; y si el VPH se puede transmitir aún cuando las VG no son visibles o no están presentes.
  - Poblaciones o grupos específicos más expuestos: Si el VPH o las VG afectan tanto a los hombres gay como a los heterosexuales; o si las VG afectan solo a los homosexuales, o a las mujeres, o solo a quienes tienen Sida.
  - Identificación: Cómo reconocer las VG tempranamente, y luego de cuánto tiempo después de la infección por VPH comienzan a aparecer las VG; cómo diferenciar entre las VG y otras ITS; entre las VG y las hemorroides; entre las VG y otras verrugas, o entre verrugas causadas por el VPH y verrugas originadas por otras causas; si es posible inferir la presencia del VPH en una persona solo por su apariencia externa; cómo manejar situaciones en las que uno se encuentra con alguien que tiene VG, o cuando la pareja descubre que uno mismo las tiene.
  - Manifestaciones: Cómo y en qué partes del cuerpo o de los genitales se presentan las VG, si pueden aparecer en la boca o en las manos; o si pueden aparecer a consecuencia del “estrés”; si existe un mayor peligro en tener VG más desarrolladas, o si el peligro es el mismo que si las VG fueran pequeñas; si puede una persona tener VPH y ser “asintomático”.
  - Tratamiento y prevención: Si existe tratamiento, si el tratamiento (la cauterización, por ejemplo) elimina el virus del VPH, o no, y si las VG pueden volver a aparecer luego de recibir un tratamiento; qué tan eficaces son los tratamientos existentes; qué debe hacer la persona que tiene VG para cuidar su salud, y para evitar transmitir el VPH a sus parejas; si la aparición de VG tiene que ver o no con el “aseo” o la higiene; si el condón es eficaz para prevenir la transmisión del VPH, o qué tan eficaz puede ser; si los servicios de salud públicos cuentan con capacidades y personal especializado en el manejo de VG.
  - Consecuencias y relación con el VIH: Cuáles pueden ser las consecuencias para la vida, la salud y la sexualidad de tener VG; qué malestares físicos producen las VG; si se podría producir cáncer anal, cómo o porqué, y si el cáncer se podría presentar también en el pene; si las VG hacen que uno sea más propenso a adquirir el VIH u otras ITS.
  - Vacunas: qué ocurre si se le aplica la vacuna preventiva a una persona que ya tiene el VPH, si esto afectará su salud o no; si tendría la vacuna algún efecto sobre las VG; si la vacuna ha sido aprobada para su uso en hombres; si existen rangos de edades apropiadas para el uso de la vacuna; si se aplica en una sola dosis, o en varias, y si fueran varias dosis, con qué periodicidad se aplicaría; cuáles son los riesgos, los efectos adversos; cómo y en qué lugar del cuerpo se aplicaría la vacuna.
  - Estudio de vacunas: cuándo se iniciaría el estudio; si representaría algún costo para los participantes; y qué institución realizaría el estudio.
- Por la misma escasez de información sobre VPH-VG, suena razonable la sugerencia de realizar actividades de difusión de información (“campañas informativas”) previas a las actividades de reclutamiento, en la perspectiva de anticipar posibles reacciones imprevistas vinculadas con los prejuicios y el estigma asociados a las VG.
- Cierto énfasis en el mensaje de que el VPH afecta también a los hombres, y no solo a las mujeres, puede contribuir a una mejor recepción del estudio entre las redes familiares de los posibles voluntarios, siendo que el “papiloma” es posiblemente más conocido entre las mujeres (p. ej. madres y hermanas de los voluntarios), y esto podría favorecer actitudes de apoyo, sobre todo desde los miembros femeninos del entorno familiar.
- El empleo de imágenes explícitas de VG parece ser más recomendable para actividades como consejería individual y actividades educativas grupales conducidas por especialistas o personal bien entrenado. Su uso en actividades extramurales de reclutamiento (en calles o locales de diversión) podría generar impresiones o impactos con resultados imprevistos si los reclutadores no tuvieran durante el trabajo de campo el tiempo y espacio suficientes para absolver dudas o manejar reacciones causadas por la visualización de tales imágenes. Por el estigma asociado a las VG, la sola difusión de imágenes de VG, sin la adecuada información al respecto, podría contribuir a mayores actitudes de rechazo o estigmatización hacia quienes tienen o se piensa que tienen VG.
- Sería recomendable explorar más a fondo las expectativas de los posibles voluntarios con respecto a cómo perciben los beneficios que recibirán por su participación en un proyecto, para tener una idea más clara de aquellos elementos que estarían funcionando como motivadores de una mayor voluntad para participar. Los hallazgos de este estudio indican que la idea de “recibir información” sería un motivador importante. Por eso, al plantear a la gente que se vacunen o que participen en un estudio, más allá de apelar a los beneficios asociados con la salud (protección frente a una ITS), se podría incidir también en el hecho de que la vacunación misma o la participación en el estudio significarán una oportunidad para recibir un tipo de información que mucha gente no maneja o desconoce.
- Otro aspecto que apareció reiteradamente en el estudio es la preocupación por las parejas sexuales (temor a transmitirles alguna infección, interés por su salud, expectativas ante sus opiniones o reacciones, etc.) Los mensajes de comunicación y los modelos de consejería podrían recoger este aspecto, según el cual la salud y la sexualidad no siempre son asuntos meramente individuales, sino también relacionales, que involucran a las parejas. Los mensajes podrían enfatizar, por ejemplo, que la vacuna les ayudará no solo a prevenir una infección en ellos mismos, sino que les ayudará también a proteger la salud de sus parejas.
- Siendo que para muchos la idea de “vacuna” no necesariamente se refiere a la “prevención”, y que para muchos “vacuna” parece tener el mismo sentido que “cura” o “tratamiento”, conviene poner énfasis en que la vacuna contra el VPH sirve para prevenir la infección en quienes no la tienen aún. Se puede incluso hablar de “vacuna preventiva” en las estrategias de comunicación (evitando emplear el término “vacuna” de manera aislada.)
- Es preciso tomar en cuenta en las estrategias de comunicación que hay una posibilidad latente de que algunos sectores sociedad asocien las VG con la población HSH o con la comunidad *gay*, lo cual podría resultar en actitudes de estigmatización hacia quienes integran estas poblaciones o hacia los voluntarios en el estudio. Algunos jóvenes *mostaceros*, por ejemplo, asociaban las VG con los *mariconcitos*, o con una “baja de defensas” en el sistema inmune (vinculando las VG con el Sida).
- En relación con el punto anterior, y por el silencio, la vergüenza y el estigma que rodean a las VG, conviene diseñar estrategias de comunicación y mensajes enfocados específicamente en estos asuntos, que se presentan hasta ahora como la mayor barrera para un posible estudio.
- Aunque para los participantes era improbable la “desinhibición” en las prácticas sexuales como resultado de haber recibido la vacuna, no está de más proponer mensajes de comunicación y consejería que incidan en la idea de los múltiples riesgos por la existencia de diversas ITS, y en la noción de que la vacuna protegería solo contra una de ellas.
- Los datos de este estudio sugieren que una mayor auto-percepción del riesgo frente a las ITS podría ser un buen indicador (o predictor) de una mayor voluntad para participar en estudios o intervenciones de prevención. Otro indicador sería el haber participado en proyectos previos, y el haber tenido experiencias positivas en tales proyectos.
- Las diferencias entre las travestis y otros grupos sugieren también la conveniencia de ensayar estrategias de reclutamiento diferenciadas para las distintas subpoblaciones. Los hombres gay preferían formas personalizadas de contacto en lugares de diversión en que se propician encuentros de sexo ocasional. Para los jóvenes *mostaceros* la confidencialidad parece ser un aspecto muy importante. Las travestis se inclinaban más por las “charlas” grupales en lugares privados [lo cual puede tener relación con el hecho de que, por la alta estigmatización que sufren las travestis de Lima, muchas prefieren no salir solas de sus domicilios, o no salir durante el día]. Y la misma marginación social hacia ellas puede tener relación con la idea de que su participación en un estudio habría de ser el resultado de un esquema de reciprocidad, en el que ellas perciban que reciben beneficios concretos (se mencionó, por ejemplo, que las “charlas” informativas debían incluir refrigerios, o que los organizadores del estudio podrían ofrecerles servicios adicionales, como capacitación, con lo que la participación en el estudio sería percibida como una forma de *colaboración mutua*.)
- Si la participación en un estudio coloca a algunas personas en posición de recibir beneficios (información, servicios de salud, la vacuna misma, etc.), es posible que quienes no puedan participar (por tener el VPH, por ejemplo) lleguen a sentirse marginados de tales beneficios. Sin embargo, una persona que había tenido VG sugirió que podría colaborar con el estudio motivando a sus pares a participar “para que no les pase lo mismo”. Conviene evaluar entonces las posibles formas de involucrar a aquellas personas que por tener el VPH o por otras razones no puedan entrar al proyecto, pero que aún así muestren una voluntad de contribuir.
